# Intratumoral androstene-3,17-dione defines breast cancer subtype and prognosis

**DOI:** 10.1038/s44321-026-00530-5

**Published:** 2026-10-01

**Authors:** Sarah Asemota, Hyo Young Choi, Wendy Effah, Jeremiah Holt, Kirsten Young, Linnea Cripe, Keely McNamara, Dong Jun Byun, Hae Jin Seo, Suriyan Ponnusamy, Daniel Johnson, Jin-Young Lee, Yekta Khosrosereshki, Brandy Grimes, Martin D Fleming, Frances E Pritchard, Ashley Hendrix, Michael Berry, Richard Fine, Richard Gilmore, Farhan Khan, Liza Makowski, Balaji Santhanam, D Neil Hayes, Hironobu Sasano, Man Ho Choi, Ramesh Narayanan

**Affiliations:** 1 Department of Medicine, College of Medicine, University of Tennessee Health Science Center, Memphis, TN USA; 2 Department of Preventive Medicine, College of Medicine, University of Tennessee Health Science Center, Memphis, TN USA; 3 UTHSC Center for Cancer Research, University of Tennessee Health Science Center, Memphis, TN USA; 4 Department of Surgery, College of Medicine, University of Tennessee Health Science Center, Memphis, TN USA; 5 Department of Pathology, Tohoku University Graduate School of Medicine, Sendai, Japan; 6 Center for Advanced Biomolecular Recognition, Korea Institute of Science and Technology, Seoul, Republic of Korea; 7 Molecular Bioinformatics Core, University of Tennessee Health Science Center, Memphis, TN USA; 8 West Cancer Center and Research Institute, Memphis, TN USA; 9 Department of Pathology, Methodist Hospital, Memphis, TN USA; 10 Center of Excellence for Data Driven Discovery and Department of Structural Biology, St. Jude Children’s Research Hospital, Memphis, TN USA

**Keywords:** Cancer, Metabolism

## Abstract

Androgens inhibit breast cancer (BC) growth in estrogen receptor (ER)-positive BC and promote growth in ER-negative BC. Correlating intratumoral androgens, instead of circulating androgens, and their downstream transcriptome, a measure of free unbound androgen function, may offer a direct assessment of androgen action in BC. Intratumoral androgen androstenedione (A4) levels in BC specimens correlated with 1388 genes, which classified patients into low, intermediate, and high A4 levels. This A4-high patients’ transcriptome was associated with lower tumor cell proliferation, earlier stage and grade, and longer survival, while A4-low patients’ transcriptome was associated with the triple-negative subtype. We developed a 90-gene A4-associated transcriptional classifier that captures intratumoral A4-associated biology. The signature requires independent validation. snRNAseq analysis indicated that high A4 specimens were enriched for favorable cell lineage, while low A4 specimens were enriched for aggressive cell lineage and cancer hallmarks. Supporting in vitro evidence suggests that A4 can inhibit ER-positive AR-positive BC cells. Collectively, these data show that intratumoral A4 levels are associated with a transcriptional state linked to lower proliferation, more luminal features, and better BC outcome.

## Synopsis

Preclinical studies and circulating androgen levels suggest that androgens have distinct effects in breast cancer, inhibiting tumor growth in estrogen receptor (ER)-positive breast cancer while promoting growth in triple-negative breast cancer (TNBC).

- An intratumoral androstenedione (A4)-dependent signature correlates with favorable outcomes in patients with breast cancer.
- The A4-dependent signature inversely correlates with Ki67, tumor stage, and grade, and positively correlates with patient survival.
- The association of the signature with outcome is subtype-dependent, with a strong correlation in ER-positive breast cancer and little to no correlation in TNBC.
- The effects of A4 in breast cancer are mediated by its receptor, the androgen receptor (AR).

## Abstract (web summary)

Preclinical studies and circulating androgen levels suggest that androgens have distinct effects in breast cancer, inhibiting tumor growth in estrogen receptor (ER)-positive breast cancer while promoting growth in triple-negative breast cancer (TNBC).

## Graphical abstract

**Figure:**
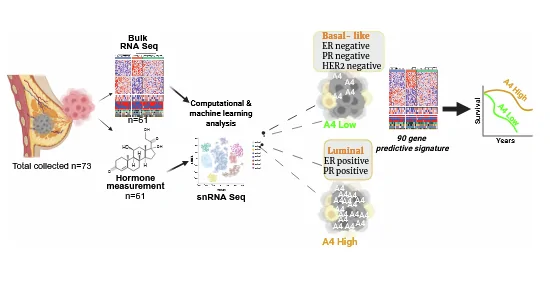

The paper explained **Problem**

Breast cancer (BC) growth is dependent on the levels of hormones such as estrogens, progesterone, androgens, and corticosteroids in the tumor cells. Preclinical studies have demonstrated that androgens and their receptor, the androgen receptor, are growth inhibitors in estrogen receptor-positive BC and growth promoters in triple-negative BC. These preclinical studies require clinical validation to confirm the role of androgens in BC. Circulating hormones are converted intratumorally by steroidogenic enzymes that eventually play a role in determining the growth and aggressiveness of the cancer. Since measuring circulating hormone levels does not account for the level of hormones in the tumor tissues, we quantified androgens intratumorally and correlated the androgen-driven gene expression signature with disease outcomes such as tumor proliferation, stage, grade, and survival.

**Results**

Measurement of intratumoral androgens from BC patients’ specimens suggested that an intratumoral androstenedione (A4)-correlated transcriptome was associated with lower tumor cell proliferation, stage, and grade, and longer survival, particularly in estrogen receptor-positive patients. A 90-gene A4-driven predictive signature was identified, which correlated with A4 levels and predicted clinical properties. Single-nucleotide RNA sequencing validated the correlation obtained with the bulk RNA sequencing data.

**Impact**

The study defined an A4-dependent signature in BC that is capable of predicting clinical outcomes of BC. The A4-dependent signature may also provide reliable prognostic and diagnostic markers for breast cancer.

## Introduction

Breast cancer (BC), like other hormonal cancers, viz. prostate, ovarian, and endometrial cancers, is dependent on intratumoral hormonal milieu for growth (Garrido et al, 2023; Tsankof and Tziomalos, 2022). Preclinical and clinical studies have extensively evaluated the role of various hormonal classes (estrogens, androgens, corticosteroids, and progestogens) in the initiation, progression, and metastasis of BC (Africander and Storbeck, 2018; Endogenous et al, 2013; Kohama et al, 1997; Mohammed et al, 2015; Schneider et al, 1990). While studies with estrogens and estrogen receptor (ER), the most evaluated hormone and the respective receptor in BC, have provided evidence for their growth-promoting role in BC, androgens and their receptor, androgen receptor (AR), have been shown to have context-dependent role, with AR playing a tumor suppressor function in ER+ve BC and an oncogenic role in triple-negative BC (TNBC) (Asemota et al, 2024; Asemota et al, 2023; Beatson, 1896; Clemons and Goss, 2001; Cochrane et al, 2014; Hickey et al, 2021; Ponnusamy et al, 2019a). A recent clinical trial with a selective AR modulator (SARM), an AR agonist, provided evidence for the therapeutic benefits of AR ligands in ER+ve BC (Palmieri et al, 2024).

Clinical studies to determine the role of estrogens and androgens in BC failed to take into consideration the local conversion by steroidogenic enzymes (Hong and Chen, 2011; Pasqualini and Chetrite, 2005; Song et al, 2006) and expression of free serum hormone binding globulin (SHBG)-unbound hormone, all of which have resulted in confounding results (Albers et al, 2024; Brennan et al, 1973; Bulbrook et al, 1971; Dorgan et al, 1996; Secreto et al, 1984; Wang et al, 1975). Circulating 17β-estradiol (E2), the active estrogen, is in the range of <10–400 pg/ml (Burger et al, 2008; Burger et al, 2007; Davison et al, 2005; Richardson et al, 1987) in pre- and post-menopausal women, while testosterone, the active circulating androgen, is in the range of 150–700 pg/ml (Davison et al, 2005; Zumoff et al, 1995). Androgens other than testosterone found in healthy women are androstenedione (A4; 0.5-3 ng/ml) and dehydroepiandrosterone (DHEA; 50–200 µg/ml)(Davison et al, 2005; Klinga et al, 1982), all demonstrating the abundance of androgens in circulation in women. Androgens and estrogens are synthesized in women by the ovaries, adrenal glands, and peripheral tissues (Auchus and Rainey, 2004; Burger, 2002; Nelson et al, 1999) and in local tissue and cells by what is termed intracrinology (Labrie et al, 2001). These circulating androgens enter local tissues and cells and elicit their action or are converted to more active androgen (Labrie, 2010), dihydrotestosterone (DHT) or estrogen E2, depending on the local expression of steroidogenic enzymes.

**Figure 1:**
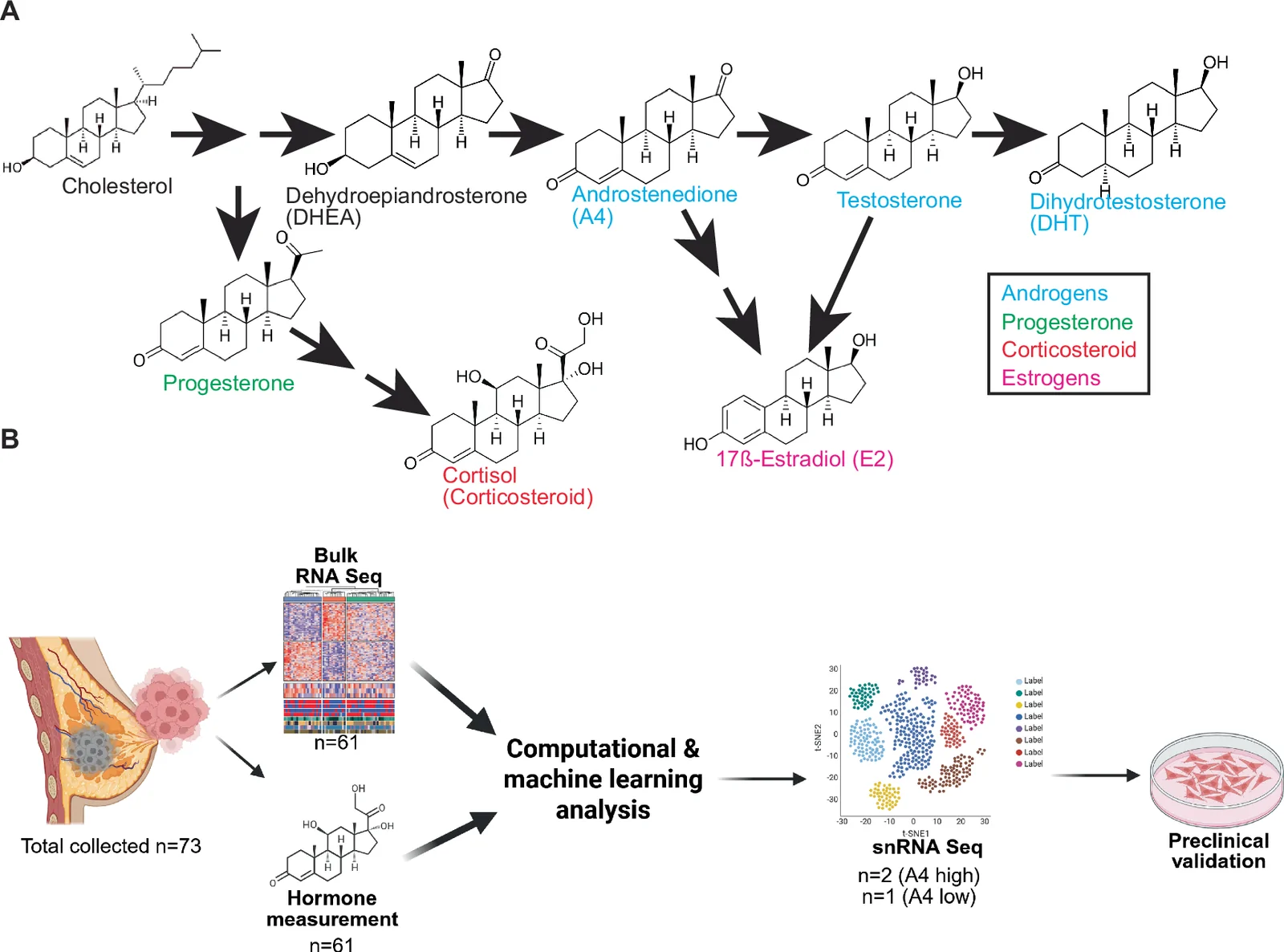
Experimental outline. (**A**) Steroidogenic pathways. (**B**) Graphical representation of methodology (Biorender). Fresh-frozen and formalin-fixed paraffin-embedded breast cancer (BC) tissues (*n* = 73) were used to measure intratumoral hormone levels and transcriptome (*n* = 61). Using computational methods and machine learning, an androgen-dependent signature was obtained. The signature and androgen-dependent clinical characteristics were validated by single-nuclear RNA seq (*n* = 3) and preclinical models (T47D, ZR-75-1 BC cell lines).

A4 is classified as an anabolic steroid due to its effects on androgenic and anabolic tissues mediated by its binding to the AR (Labrie et al, 2001; Labrie et al, 2006). While A4 activates AR starting at 10 nM (Yazawa et al, 2020), comparable to testosterone, it can also be converted to various active androgens and estrogens (Fig. 1A). A4 exhibits BC subtype-dependent properties stimulating DMBA-induced mammary carcinoma in rats but inhibiting ZR-75-1 cell proliferation with an IC_50_ of 15 nM (Dauvois and Labrie, 1989; Poulin et al, 1988). There is also the risk of A4 being metabolized to estrogens that may promote the growth of BC. This data does not provide conclusive evidence for the role of androgens in BC in the clinical setting. The activity of androgens is also dependent on the expression of and interaction with various cofactors and receptors, and expression of SHBG in the local tissues (Drummond et al, 2022; Miller et al, 2013).

Considering the complexity of intracrinology, robust steroidogenic pathways, and differences in the expression of ER, PR, HER2, AR, SHBG, and cofactors we embarked on a unique approach to measure intratumoral total androgens and transcriptome to secure conclusive evidence for the role of androgens on tumor growth and other clinical variables in BC (Fig. 1B). Since only free androgens (a form that is difficult to measure clinically) are active, androgen function can be determined from its correlative gene signature. The data suggest that intratumoral A4 clustered patients into groups with distinct transcriptomes. The high-A4 group’s transcriptome was associated with lower BC cell proliferation, earlier stage and grade, and better survival. The data also provided an A4-correlative 90 gene signature.

## Results

### Clinical and tumor characteristics

To pursue our strategy to measure intratumoral hormone levels followed by omics analysis, BC specimens that are devoid of adjacent fatty tissues were collected from the surgical suite and snap-frozen or formalin-fixed (Fig. 1B). Part of the tissue was used for hormone measurement (focused on DHEA, A4, testosterone, and DHT). Although DHT was measured, its levels were lower than the lower limit of quantification (LLOQ) in many specimens and hence were not taken into further analyses. DHEA and A4 (Fig. 1A) are considered androgen precursors, though they have direct AR agonistic activity, with A4 activating AR at ~10–30 nM (Yazawa et al, 2020).

The 73 patient specimens for which hormone measurements are available represent an equal number of women belonging to the African American (AA) and Caucasian American (CA) races, the typical demographic distribution of the Memphis metropolitan area (Table EV1). Age cutoff for low and high was taken at 50 years. More than 50% of the specimens are ER+ve and HER2-ve, and about 70% of the specimens are AR+ve. Treatment response and survival were also recorded.

### Correlation between intratumoral hormone and histological characteristics

**Figure 2:**
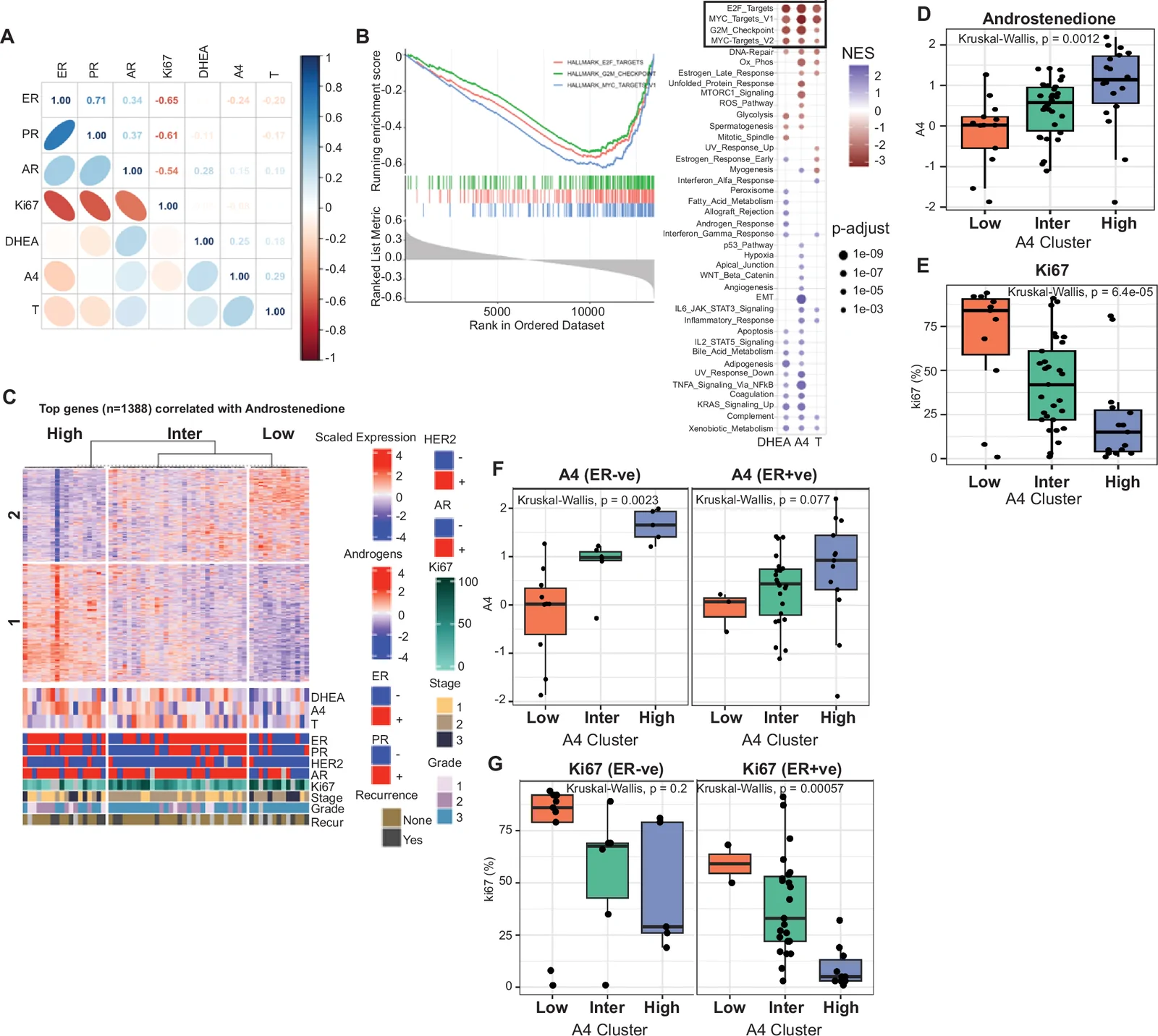
Androstenedione-dependent gene expression signature correlates with better breast cancer (BC) clinical outcome. Androstenedione (A4), dehydroepiandrosterone (DHEA), and testosterone (T) were measured by gas chromatography mass spectrometry (GC-MS) method in fresh-frozen tumors (*n* = 73). Clinical and immunohistochemical properties were determined. RNA sequencing was performed in FFPE tissues (*n* = 73) (*n* = 61 had usable data). Immunohistochemical markers such as ER, PR, AR, HER-2, and hormone levels were correlated (**A**). (**B**) Gene set enrichment analysis (GSEA) of the transcriptome and intratumoral A4 levels (*n* = 61). Hallmark pathways significantly associated with A4, DHEA, and testosterone (T) are shown. Gene sets were ranked according to the Pearson correlation between gene expression and hormone levels, and enrichment significance was assessed using the GSEA algorithm. Normalized enrichment scores (NES) and false discovery rate (FDR)-adjusted *P* values are displayed. (**C**) A4 levels and transcriptome were measured in BC specimens (*n* = 61). Unsupervised k-means clustering was performed to determine the genes that correlated with A4. The 1388 genes that correlated with A4 (q < 0.1) are provided in the heatmap. The heatmap also shows the various measured variables. The correlation classified the samples into low, inter, and high based on A4 levels and into groups 1 and 2 based on gene expression pattern. (**D**, **E**) A4 levels (**D**), ki67 expression (*n* = 61) (**E**) between three A4-dependent gene clusters (low, inter, and high). With increasing A4 levels ki67 decreased (*n *= 61). (**F**, **G**) A4 levels (**F**) and corresponding ki67 expression (**G**) in ER+ve and ER-ve specimens (*n* = 61). Statistical methods are provided in the figure panels and “Methods”. Box plots show the median (center line), interquartile range (box boundaries, 25th and 75th percentiles), and whiskers extending to the most extreme data points within 1.5 × the interquartile range. Individual data points are shown. A4 androstenedione, DHEA dehydroepiandrosterone, T testosterone, ER estrogen receptor, PR progesterone receptor, AR androgen receptor, HER-2 human epidermal growth factor receptor-2, Low low A4 levels, inter intermediate A4 levels, High high A4 levels, ER-ve estrogen receptor negative, ER+ve estrogen receptor-positive, ki67 proliferation marker, NES normalized enrichment score.

ER and PR expression positively correlated with each other, as anticipated, and ER and PR expression also positively correlated with AR expression (Fig. 2A), supporting the literature evidence that most ER+ve tumors are also AR+ve (Hickey et al, 2021; Ponnusamy et al, 2019a). As expected, due to their ability to differentiate cells, ER, PR, and AR negatively correlated with ki67, with a strong negative correlation observed with all three receptors.

DHEA, A4, and testosterone positively (weakly) correlated with each other, which is expected as they are precursors and products in the androgen metabolism pathway. However, the hormones by themselves had a weak negative correlation with the proliferation marker ki67. As only total hormone levels were measured, and only free SHBG unbound hormones are functional, the weak correlation of total hormone with ki67 was not a surprising outcome. A4 levels range between 0.05 and 23 ng/g tumor tissue. DHEA levels were between 0.1 and 70 ng/g tissue, and testosterone levels ranged between 0.06 and 17 ng/g tumor. Steroid concentrations were log-transformed to reduce skewness and then standardized to z-scores (mean = 0, standard deviation = 1) for downstream analyses. Therefore, the values shown in the figures represent standardized log-transformed steroid levels. We also measured estrone levels and aromatase gene expression and found no correlation between any clinical variable, gene expression, and estrone levels. Hence, estrone was not taken into consideration for further analysis.

**Figure EV1:**
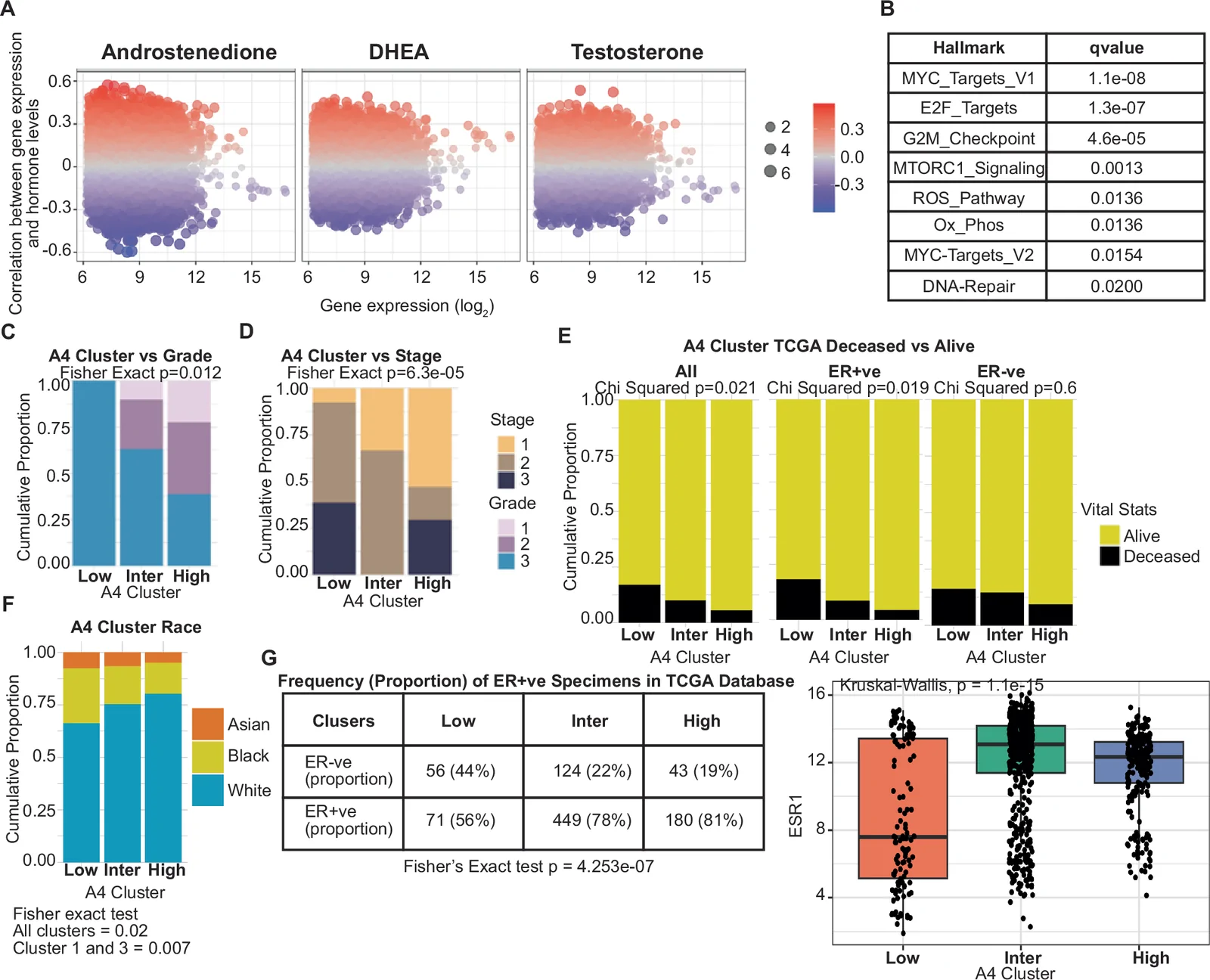
A4-dependent signature correlates with better prognosis. (**A**) Correlation between gene expression and various hormone levels. (**B**) *P* values of the top pathways that are negatively correlated with A4 levels. The 1388 genes significantly correlated with A4 were used for unsupervised subtype detection by K-means clustering (*n* = 61). Tumor grade (**C**) and stage (**D**) between three A4-dependent gene clusters. (**E**, **F**) Clinical and gene expression data extracted from the TCGA BC database (*n* = 973) were used to create clusters with 1231 of 1388 genes identified in Fig. 2. (**E**) Alive and deceased patients separated according to the three A4 clusters. (**F**) Proportion of patients’ race in different clusters in the TCGA dataset. (**G**) Expression of ER+ve and ER-ve specimens in each cluster in the TCGA dataset (*n* = 923). Right: ESR1 gene expression in the TCGA dataset separated based on the three clusters. Box plots show the median (center line), interquartile range (box boundaries, 25th and 75th percentiles), and whiskers extending to the most extreme data points within 1.5 × the interquartile range. Individual data points are shown.

As genes that correlate with a hormone will be a measure of the respective hormone’s activity, RNA sequencing was performed in 73 specimens (61 specimens had usable data), and the genes (13,458) were correlated with tumor DHEA, A4, and testosterone levels. With a false discovery rate (FDR) of *q* < 0.1, 1388 genes (*q* < 0.05 = 497 genes) significantly correlated (positively and negatively) with A4 (Fig. EV1A; Datasets EV1 and EV2). Interestingly, no genes and only 2 genes significantly correlated with DHEA and testosterone, respectively. Hence, further analysis was performed with A4, and the list of A4-correlated genes was cross-validated with DHEA and testosterone. The genes were rank-ordered and the positively and negatively correlated genes were used to conduct hallmark pathway analysis using GSEA. The top three hallmark pathways that negatively correlated with A4 in BC were E2F, Myc, and G2M checkpoint (Figs. 2B and EV1B). These three pathway genes negatively correlated with A4 with a normalized enrichment score (NES) of ~-3 and *q* values in the range of 10^−5^ and 10^−8^. Verification of these pathways with genes in the DHEA and testosterone RNA sequencing correlated signature also showed a significant negative enrichment. Other oncogenic pathways that were also negatively correlated with A4 include mTORC1 (Fig. EV1B). We examined the overlap between the larger set of A4-associated genes and AR-target genes and established androgen-related gene sets. Among the 1388 A4-associated genes, 98 overlapped with androgen-related gene sets curated in MSigDB, including 11 genes present in the HALLMARK_ANDROGEN_RESPONSE gene set (CDK6, HMGCS1, LIFR, MAP7, PIAS1, PLPP1, PTPN21, SAT1, SLC38A2, SMS, TPD52). These data suggest that there is an enrichment of an androgen-dependent signature.

### A4-correlated genes form distinct transcriptional states across tumors

An unsupervised k-means clustering algorithm was applied to the 1388 genes whose expression significantly correlated with A4 (Fig. 2C). This clustering resulted in three tumor groups and two gene clusters, with gene expression patterns changing in a gradual manner across the three tumor groups, suggesting a continuous transcriptional gradient. Because genes were selected based on correlation with A4 levels, separation of tumors along the A4 axis is expected. The three transcriptional groups corresponded to tumors with low, intermediate, and high A4 levels, and were therefore labeled low-, intermediate-, and high-A4 clusters. DHEA and testosterone levels showed similar increasing trends across the three groups, although the trend for testosterone did not reach statistical significance (Fig. 2C). Importantly, clinical variables that were not used in either gene selection or clustering differed significantly across A4-associated groups. The proliferation marker ki67, tumor grade, and stage all significantly decreased from patient cluster A4 low to high (Figs. 2D and EV1C,D). In other words, various cancer aggressive, metastatic, and progression markers negatively correlated with androgen-driven transcriptome, especially with A4. A4 cluster low had 100% grade 3 tumors, while A4 cluster high had the least grade 3 tumors (*P* value = 0.012; Fisher’s Exact test). Also, A4 cluster low had the least stage 1 tumors, while the majority of the specimens in A4 cluster high had stage 1 tumors (*P* value < 10^−5^, Fisher’s exact test) (Figs. EV1C,D). These results suggest that the primary source of variation among tumors is a continuous A4-associated transcriptional axis and that k-means clustering effectively discretizes this gradient into low, intermediate, and high states, which show clinical relevance. One interesting observation made in the patient clusters was that the A4 cluster low had an enrichment of TNBC tumors, while the A4 cluster high had an enrichment of ER+ve, PR+ve tumors. A4 cluster inter had a mix of both TNBC and ER+ve tumors. These findings indicate that the A4-associated transcriptional gradient is associated with clinically relevant differences in tumor aggressiveness.

Since preclinical studies suggest that AR is a tumor suppressor in ER+ve BC and an oncogene in TNBC, we evaluated the clusters for any differences between ER+ve and ER-ve specimens (Fig. 2F,G). As expected, A4 levels inversely correlated with ki67 in ER+ve tumors, and the ki67 decrease was statistically significant. A4 levels also significantly increased from A4 clusters low to high in ER-ve specimens, but no corresponding decrease in ki67 was observed between A4 clusters low to high, consistent with previous preclinical findings that androgens act as tumor suppressors in ER+ve, but not in ER-ve BC (Lehmann et al, 2011).

### Prediction of the A4 clusters in the TCGA BC database

**Figure 3:**
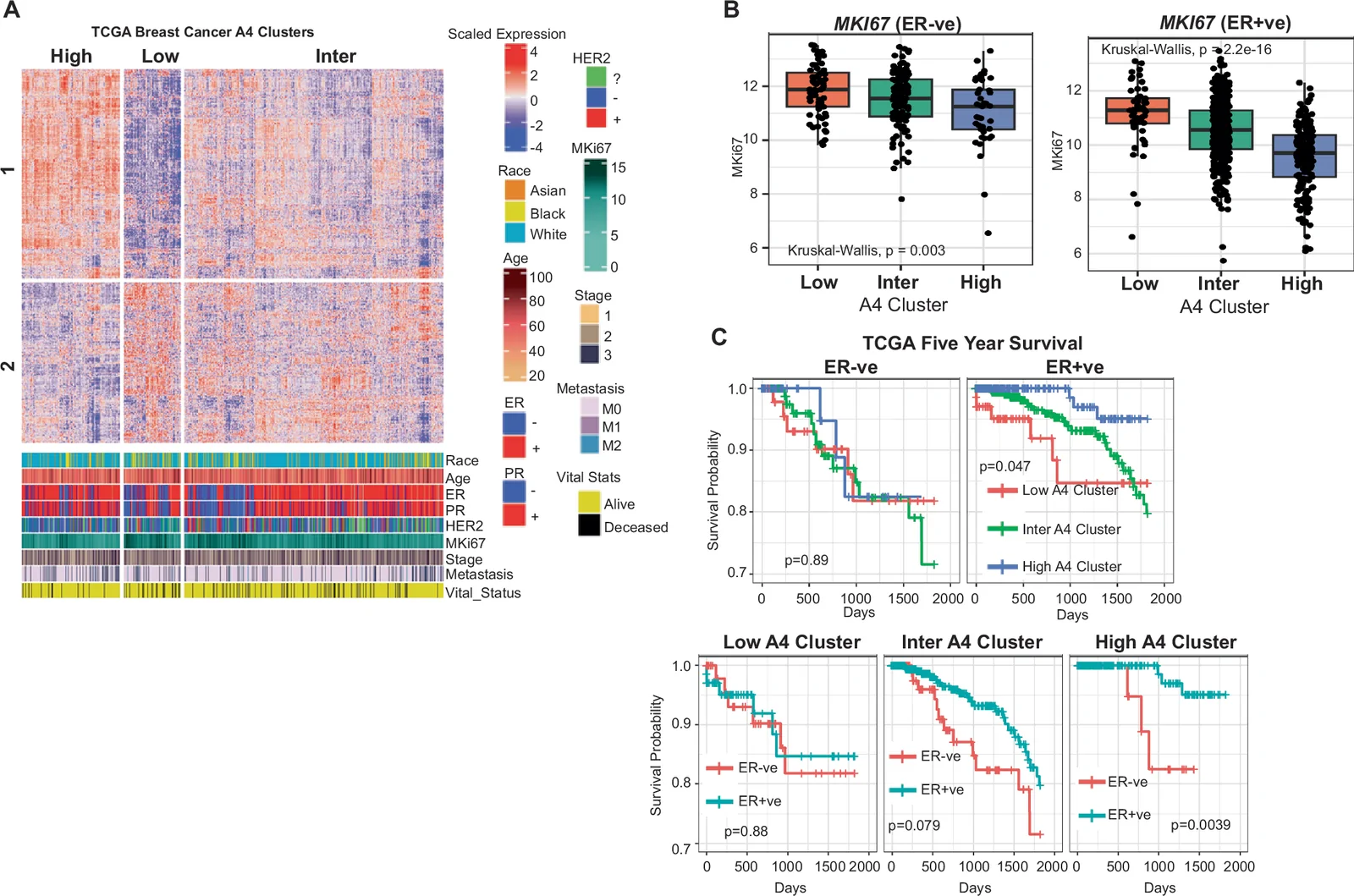
Validation of clinical data outcome using the TCGA database. The 1388 genes (1231 genes of the 1388 genes overlap) were used in the TCGA breast cancer (BC) database to predict the three clusters using a centroid-based prediction approach. The clinical and histological characteristics of the patients in the three clusters were analyzed to determine their properties. Since the TCGA dataset lacks the proliferation marker ki67, its gene *MKI67* was used for the analysis. Since the TCGA dataset has survival data, patient survival across the three patient clusters was evaluated. (**A**) Clinical and gene expression data extracted from the TCGA BC database (*n* = 973) were used to create clusters with 1231 of 1388 genes identified in Fig. 2. (**B**) MKI67 gene expression levels in the TCGA dataset that were segregated into three A4 clusters (*n* = 923; ER status was unavailable for 50 out of the 973 patients). (**C**) Kaplan–Meier (KM) plot of patient 5-year survival of the TCGA dataset (*n* = 923). Survival differences between groups were evaluated using the log-rank test. KM plots were obtained for ER+ve, ER-ve, and for the three clusters comparing ER+ve and ER-ve specimens. TCGA The Cancer Genome Atlas, ER estrogen receptor, PR progesterone receptor, AR androgen receptor, HER-2 human epidermal growth factor receptor-2, Low low A4 levels, Inter intermediate A4 levels, High high A4 levels, ER-ve estrogen receptor negative, ER+ve estrogen receptor-positive, *MKI67* gene for proliferation marker ki67. Box plots show the median (center line), interquartile range (box boundaries, 25th and 75th percentiles), and whiskers extending to the most extreme data points within 1.5 × the interquartile range. Individual data points are shown.

The 1388 genes (1231 genes of the 1388 genes overlap) were used in the TCGA BC database to predict the three clusters using a centroid-based prediction approach (“Methods”). Among the 973 TCGA BC samples, 13% were classified into the A4-low cluster, 22.9% into the A4-high, and the remaining samples into the A4-inter (Fig. 3A). The clinical and histological characteristics of the patients in the three clusters were analyzed to determine their properties. Since the TCGA dataset lacks ki67 measurements, *MKI67* expression was used as a surrogate proliferation marker. In our clinical cohort, *MKI67* expression was highly correlated with both ki67 and the PAM50-based proliferation score, supporting its use as a representative RNA-based proliferation readout (Table EV2). Consistent with observations from our internal dataset, *MKI67* decreased progressively from the A4 low cluster to the A4 high cluster in the TCGA dataset (Fig. 3B). This decrease was evident in both ER+ve and ER-ve specimens, though ER-ve specimens’ significance was much weaker than ER+ve specimens.

Since the TCGA dataset has survival data, we evaluated patient survival across the three patient clusters. The number of deceased patients decreased from A4 low cluster to A4 high cluster (Fig. EV1E). This trend was primarily observed in ER+ve patients only (*P* value = 0.019, Fisher’s exact test). To further investigate, Kaplan–Meier survival curves were compared between the clusters in ER+ve and ER-ve specimens to assess 5-year survival rates across the clusters. In ER+ve specimens, notably, 5-year survival was significantly higher in the A4 high cluster compared to the A4 low cluster. However, this difference was not statistically significant in ER-ve specimens (Fig. 3C). Interestingly, ER+ specimens in A4 low cluster with low A4 levels exhibited survival rates comparable to those of ER-ve specimens in A4 low cluster. This indicates that tumors with low A4 levels and corresponding signature may correlate with aggressive nature, irrespective of ER status.

We also assessed any racial differences between A4 low and high clusters in the TCGA dataset (Fig. EV1F). Considering that the A4 low cluster has a high proportion of TNBC patients, who are likely to be African American (AA) women (Asemota et al, 2023), we expected the A4 low cluster to have a higher proportion of AA patients. Supporting this hypothesis, the A4 low cluster was enriched with AA women, suggesting that this cohort includes a more aggressive TNBC subset, which is more prevalent in AA women.

To validate the observation made with our internal dataset that low A4 cluster is enriched for ER-ve tumors, we analyzed the TCGA dataset (Fig. EV1G). ER-ve tumors comprised 44% of the A4-low cluster, decreasing to 21.6% in the A4-inter cluster and 19.3% in the A4-high cluster. This remarkable decrease in ER-ve tumors that are considered to be less differentiated and more aggressive with an increasing A4 raises the interesting possibility that A4 or other androgens may play a role in regulating ER expression, which will require distinct mechanistic investigation. The expression of the ER gene, *ESR1*, also significantly increased from cluster low to cluster high in the TCGA dataset (Fig. EV1G, right).

### Validation of the A4 clusters using a validation cohort

**Figure EV2:**
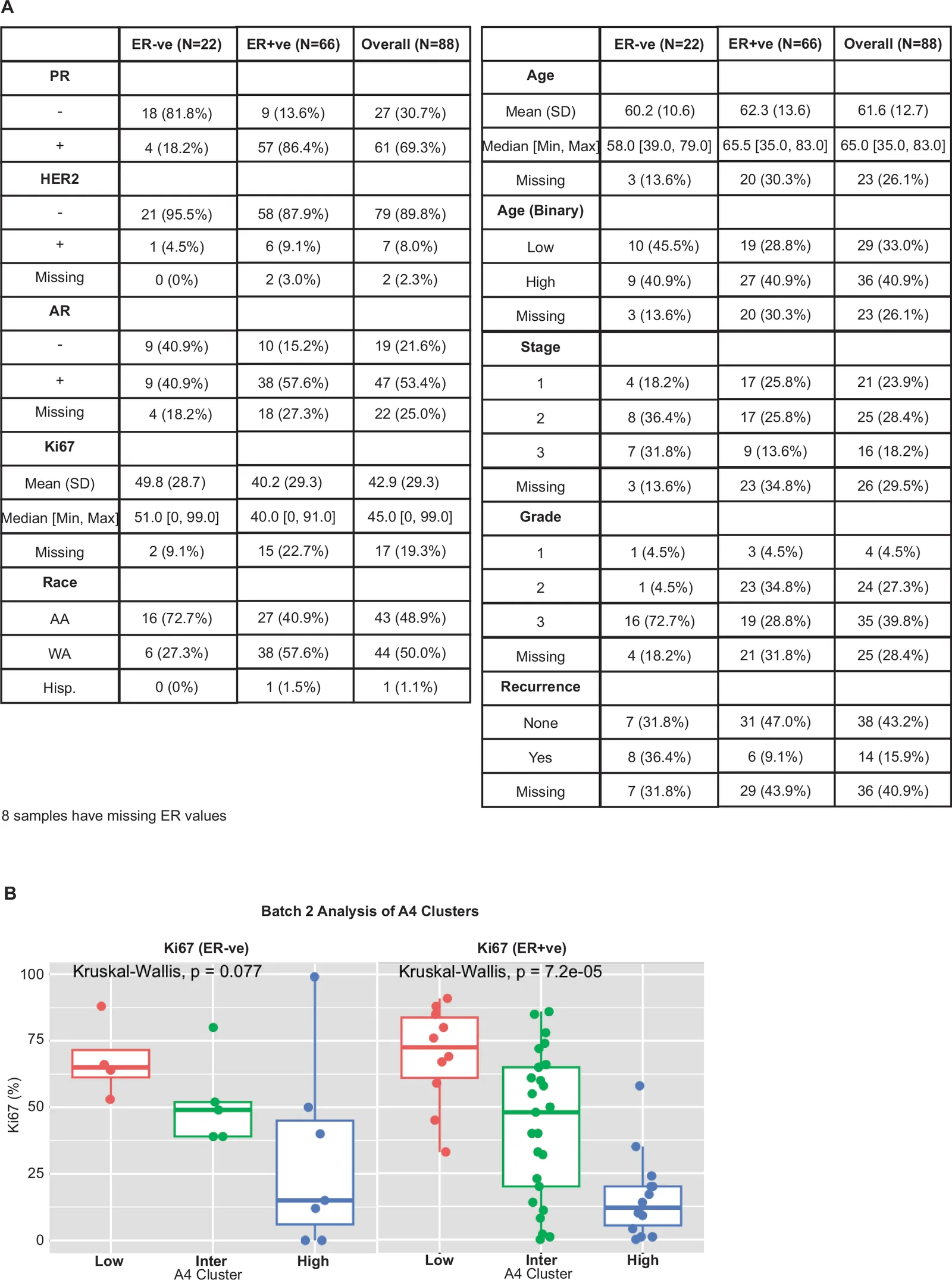
Validation Cohort. (**A**) Clinical and histological variables of validation cohort (*n* = 88). (**B**) Ki67 levels in the validation cohort based on the three clusters obtained from A4-correlated genes (*n* = 77; Gene expression data were unavailable for 11 out of the 88 patients). Box plots show the median (center line), interquartile range (box boundaries, 25th and 75th percentiles), and whiskers extending to the most extreme data points within 1.5 × the interquartile range. Individual data points are shown.

The 1388 genes were used in a second cohort of patients (*n* = 88) to predict the three clusters. Patient characteristics were comparable to our pilot cohort (Fig. EV2A). The three clusters showed a similar trend to our original cohort, with ki67 decreasing from low A4 to high A4 cluster, reinforcing the association between higher A4 and lower proliferation observed in the original cohort (Fig. EV2B).

### A4-dependent predictor signature

**Figure EV3:**
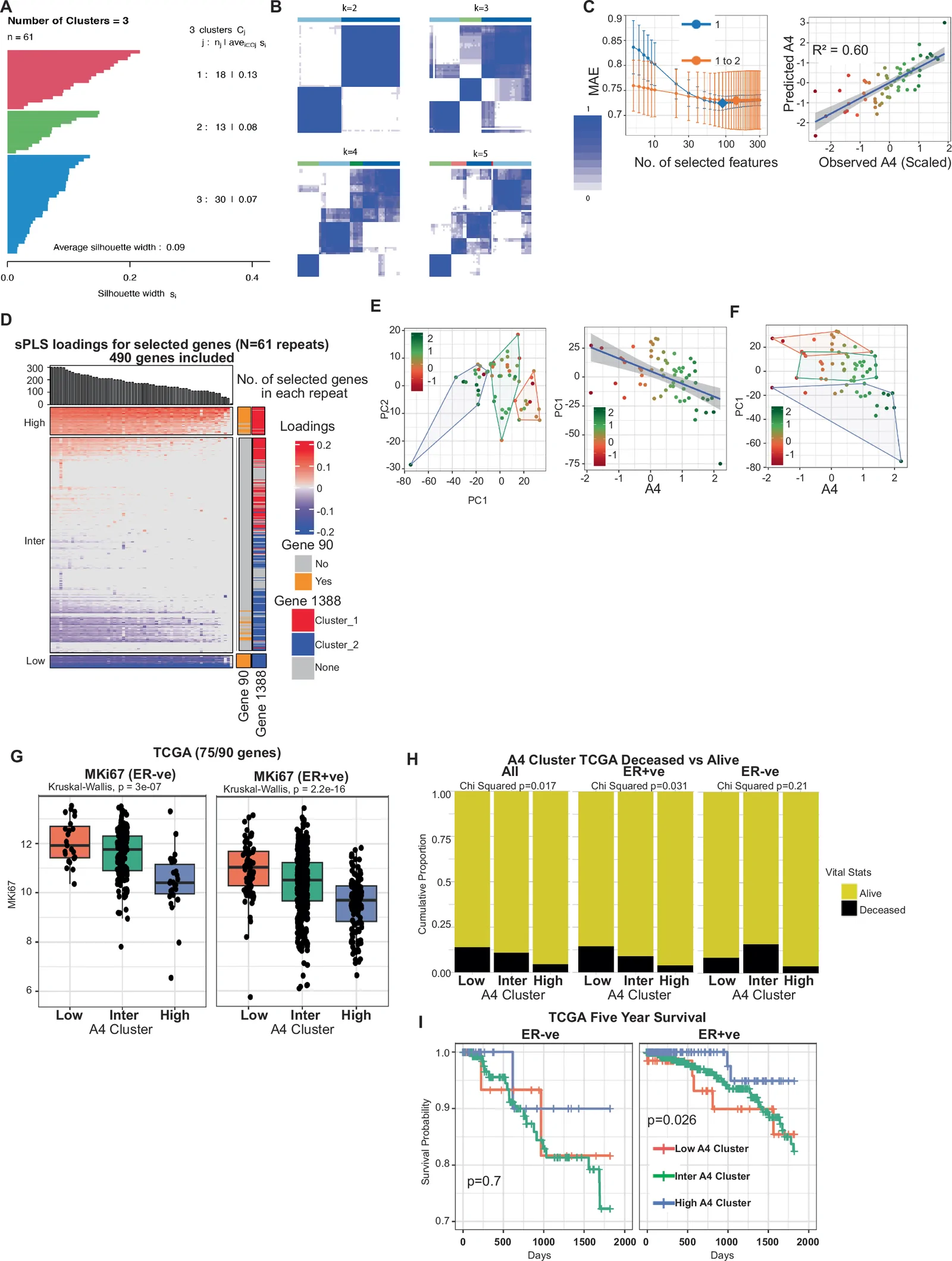
Computational analysis method to obtain the 90 gene signature. (**A**, **B**) Validation of the approach used in the main manuscript (*n* = 61). See “Methods” for details. (**C**) Mean absolute error (MAE) obtained from fivefold cross-validation across varying numbers of selected genes for comparing models with one dimension (Component 1 only) and two dimensions (Components 1 and 2) (*n* = 61). Error bars represent standard deviations across cross-validation runs. (**D**) Further validation to assess the robustness of the selected gene set (*n* = 61). Leave-one-out cross-validation (LOOCV) approach to evaluate the selected 90 genes. (**E**, **F**) Principal component analysis (PCA) on the expression data of 1388 genes previously identified as highly associated with A4 (*n* = 61). Two-dimensional representation using the first two principal components (PCs). Solid polygons represent convex hulls enclosing samples within each A4 cluster. Regression lines were fitted using linear regression, and shaded regions represent 95% confidence intervals around the fitted regression lines. (**G**–**I**) TCGA BC validation of the 90 genes (75 out of the 90 genes were detected in the TCGA cohort; *n* = 973). (**G**) MKI67 gene expression levels in the TCGA dataset that were segregated into three A4 clusters. (**H**) Alive and deceased patients separated according to the three A4 clusters. (**I**) Kaplan–Meier (KM) plot of patient 5-year survival of the TCGA dataset. Survival differences between groups were evaluated using the log-rank test. KM plots were obtained for ER+ve and ER-ve. Box plots show the median (center line), interquartile range (box boundaries, 25th and 75th percentiles), and whiskers extending to the most extreme data points within 1.5 × the interquartile range. Individual data points are shown

Directly measuring A4 levels from tumors requires advanced technologies and is often not feasible. Given the identified 1388 genes highly correlated with A4 levels, we investigated whether a subset of these genes could serve as biomarkers for predicting A4 levels (“Methods” section and Fig. EV3A–D provide details of the analyses). We first performed principal component analysis (PCA) on the expression data of 1388 genes previously identified as highly associated with A4 (*n* = 61). We found a strong correlation between PC1 and A4 levels (cor = −0.51, *P* value = 2.7^−05^), which was higher than the correlations observed between individual gene expressions and A4 levels. This indicates that while individual genes may not be strong predictors, a set of genes considered collectively might provide a powerful tool for predicting A4 levels (Fig. EV3E).

In a two-dimensional representation using the first two principal components (PCs), we observed that the first principal component (PC1) captured most of the variation separating the three sample clusters. This suggests that a single dimension alone, represented by PC1, provides sufficient discriminatory power for cluster separation (Fig. EV3F). Given the strong correlation between PC1 and A4 levels observed through an unsupervised approach, we further explored a smaller gene set for predicting A4 levels within a supervised framework based on sparse partial least squares (sPLS) (“Methods”). To select the model parameters, the lowest Minimum Absolute Error (MAE) achieved was with 90 genes, and the one-dimensional model outperformed the two-dimensional model, suggesting that adding a second component did not improve performance, consistent with our PCA results (“Methods”, Fig. EV3C).

Using these optimized parameters, we fit the final sPLS model to the full gene expression dataset, comprising 13,460 genes. This supervised framework identified 90 genes that contributed significantly to A4 prediction (Dataset EV3). Notably, 88 of these genes overlapped with the 1388 genes previously identified as highly correlated with A4, and the predicted A4 values showed a strong correlation with observed levels (correlation = 0.77, *R*^2^ = 0.6) (Fig. EV3C, left). These findings suggest that the 90-gene predictor has strong potential for inferring underlying functional A4 levels, which are otherwise difficult to measure.

The A4 predictor was further validated to assess the robustness of the selected gene set. In the absence of an external validation cohort, we employed a leave-one-out cross-validation (LOOCV) approach to evaluate whether the selected 90 genes constitute a stable and reliable gene set for A4 prediction. During each iteration, the number of identified genes varied, ranging from 6 to 300 genes (Fig. EV3D). Notably, certain gene sets were consistently selected across iterations, with the majority of the 90-gene set repeatedly emerging as key features in almost every iteration (Fig. EV3D). The loading values from each iteration represent the contribution of each gene, with higher absolute values indicating stronger contributions—positive values reflecting a positive association with A4 and negative values reflecting a negative association. It is evident that most of the 90 genes contribute significantly to A4 prediction in nearly all iterations. These findings suggest that the 90 gene set is relatively stable within this dataset; independent external validation will be needed to establish its reliability as a predictive tool.

### A reduced 90-gene classifier reproducibly captures A4 tumor states and their clinical relevance

**Figure 4:**
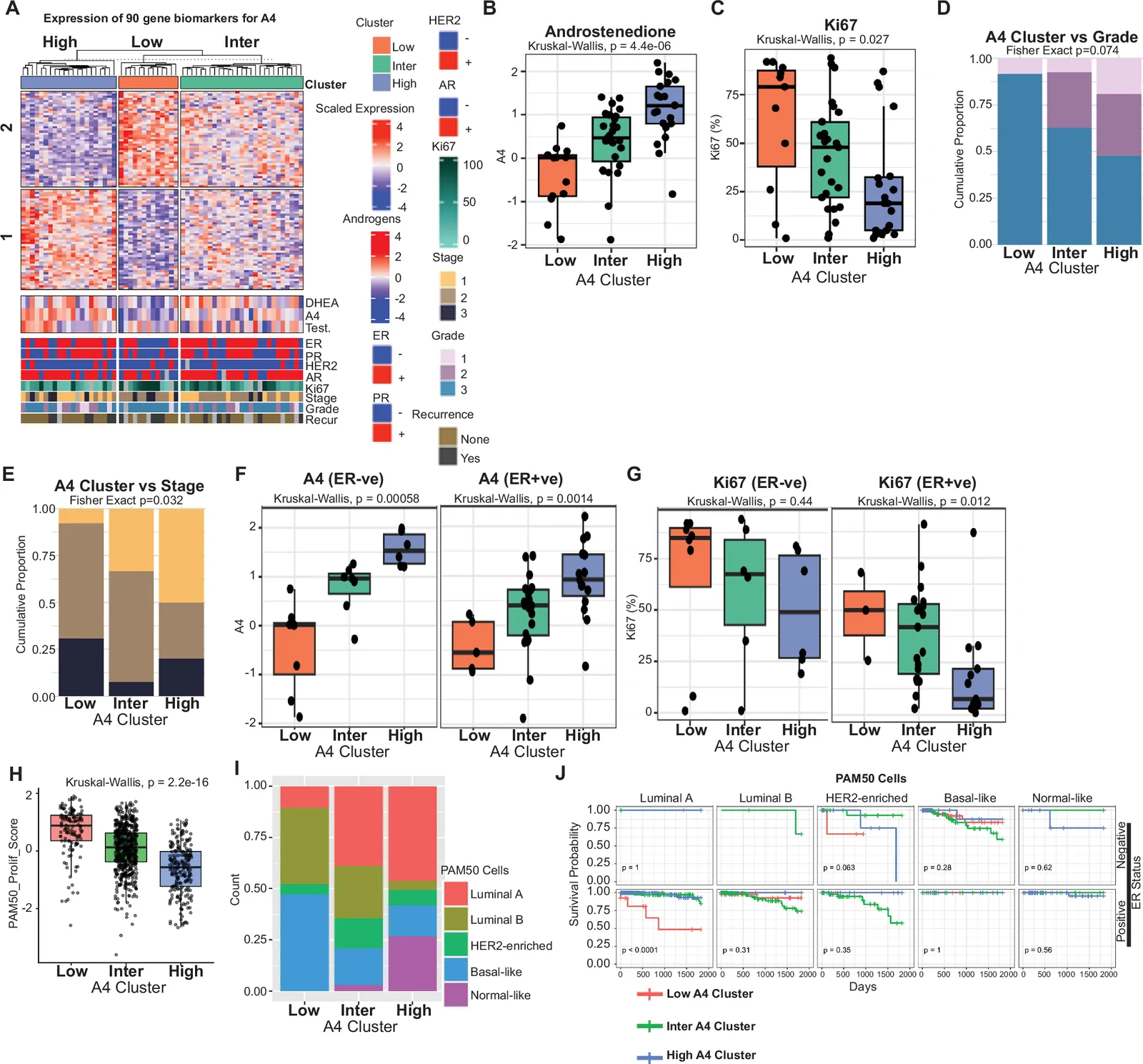
Derivation of a 90-gene signature that corresponds to intratumoral androstenedione levels. Using the reduced gene set, a 90-gene classifier based on a modified centroid prediction method was developed and evaluated for its reproducibility against the outcomes derived from the full 1388-gene set. Sparse partial least squares (sPLS), a supervised multivariate method designed to handle high-dimensional datasets, was used to identify a subset of genes most predictive of A4 levels. For the analysis, gene expression data consisting of 13,458 genes from 61 samples were used as input data. The A4 levels, which served as the response variable, were also standardized to enable direct comparison of model coefficients. All analyses were conducted using SCISSOR and “mixOmics” packages in R. (**A**) The top 90 genes significantly correlated with A4 were used for unsupervised subtype detection (*n* = 61). The heatmap shows the expression of genes and various measured variables. (**B**–**E**) A4 levels (**B**), ki67 expression (**C**), tumor grade (**D**), and stage (**E**) between three A4-dependent gene clusters. (**F**, **G**) A4 levels (**F**) and corresponding ki67 expression (**G**) in ER+ve and ER-ve specimens. (**H**–**J**) Relationship between A4 clusters, proliferation, intrinsic subtype, and survival using TCGA BRCA. Survival differences between groups were evaluated using the log-rank test. (**H**) PAM50-based proliferation scores across A4 clusters (A4-low, A4-intermediate, A4-high) in the TCGA BRCA cohort. A4 clusters show a significant association with proliferation, consistent with MKI67-based analyses. (**I**) Distribution of PAM50 intrinsic subtypes across A4 clusters. A4-low tumors are enriched for Basal-like and Luminal B subtypes, whereas A4-high tumors are enriched for Luminal A and Normal-like. (**J**) Kaplan–Meier survival curves stratified by ER status and PAM50 subtype. Differences in survival between A4 clusters were assessed using the log-rank test. In ER-positive tumors (bottom panels), A4 clusters further stratify survival within subtypes, most notably within Luminal A (*P* = 4.3 × 10⁻⁵), where A4-low tumors show worse outcomes compared to A4-high tumors. A4 androstenedione, DHEA dehydroepiandrosterone, T testosterone, ER estrogen receptor, PR progesterone receptor, AR androgen receptor, HER-2 human epidermal growth factor receptor-2, Low low A4 levels, Inter intermediate A4 levels, High high A4 levels, ER-ve estrogen receptor negative, ER+ve estrogen receptor-positive, ki67 proliferation marker, PAM50 Predictive Analysis of Microarray 50, TCGA The Cancer Genome Atlas. Box plots show the median (center line), interquartile range (box boundaries, 25th and 75th percentiles), and whiskers extending to the most extreme data points within 1.5 × the interquartile range. Individual data points are shown.

Using the reduced gene set, we developed a 90-gene classifier based on a modified centroid prediction method and evaluated whether the major biological and clinical characteristics identified using the full 1388-gene signature could be reproducibly captured using a substantially smaller gene set (“Methods”). The application of the 90-gene classifier alongside clinical data demonstrated high consistency with the results from the full gene set, with only minor discrepancies observed (4.9%, 3/61). Notably, no membership changes occurred between the A4-low and A4-high groups, indicating a clear distinction in expression profiles between these A4 clusters. The heatmap of the 90 genes and patient clusters is provided in Fig. 4A. Consistent with the full 1388-gene classifier, tumors classified as A4-low exhibited higher ki67 and a more advanced stage compared with A4-high tumors, whereas tumor grade showed a similar trend that did not reach statistical significance (Fig. 4B–E). Similar to the results obtained using the full gene set, multiple markers of cancer aggressiveness and progression were inversely associated with A4-associated states.

We evaluated the clusters for any differences between ER+ve and ER-ve specimens (Fig. 4F,G). A4 levels increased significantly from cluster low to high, both in ER+ve and ER-ve specimens. Consistent with the findings obtained using the full gene set, Ki67 expression decreased across A4 clusters in ER-positive tumors, whereas no corresponding decrease was observed in ER-negative tumors despite increasing A4 levels.

To further validate the classifier, we applied the 90-gene set to the TCGA BC dataset (Fig. EV3G–I). For this analysis, only the 75 overlapping genes were used for prediction. While a higher discrepancy was noted (22.2%, 216/973) compared to the clinical data, no membership transfers were observed between the A4-high and A4-low groups, aligning with the findings from the clinical data. This consistency underscores the robustness of the 90-gene classifier in maintaining group membership distinctions despite the reduced gene set. Similar to the ki67 in our internal dataset, *MKI67* decreased between the A4 cluster low and A4 cluster high in the TCGA dataset (Fig. EV3G). The decrease was observed in both ER+ve and ER-ve specimens.

The number of deceased patients decreased from A4 low cluster to A4 high cluster (Fig. EV3H). The observation was made in ER+ve patients only. Subsequently, a Kaplan–Meier plot was drawn between the clusters in ER+ve and ER-ve specimens to determine if 5-year survival increased in both cohorts from cluster low to cluster high. The 5-year survival of A4 cluster high was significantly higher than A4 cluster low only in ER+ve specimens (Fig. EV3I).

To assess the relationship between A4-associated gene expression and established BC classifiers, we evaluated PAM50 intrinsic subtypes and a PAM50-based proliferation score in the TCGA BRCA cohort. A4 clusters were significantly associated with proliferation inhibition, consistent with analyses using MKI67 (Fig. 4H), yet each cluster contained a mixture of subtypes (Fig. 4I). Notably, survival analyses stratified by ER status and PAM50 subtype showed that A4 clusters further stratified outcomes within the same subtype. It was most prominent within Luminal A, where A4-low tumors exhibited worse survival than A4-high tumors in ER-positive tumors (Fig. 4J).

To determine whether the prognostic effect of A4 clusters differed across intrinsic BC subtypes, we performed multivariable Cox proportional hazards analyses. In the overall cohort, the prognostic association of A4 clusters was attenuated after adjustment for PAM50 subtype, age, tumor stage, and MKI67 expression (Table EV2; Model 1). However, given prior evidence that steroid signaling exerts subtype-specific effects in BC, we next evaluated an interaction between A4 clusters and PAM50 subtype (Table EV2; Model 2). Inclusion of the interaction term significantly improved model fit compared with the model containing only main effects (likelihood ratio test, *χ*² = 16.99, df = 7, *P* = 0.017; Table EV3). These findings indicate that the association between A4-associated gene expression and survival is subtype-dependent.

Given the significant interaction between the A4 cluster and the PAM50 subtype, we next evaluated the prognostic significance of A4 clusters specifically within Luminal A tumors. In multivariable Cox proportional hazards analyses adjusting for age, tumor stage, and MKI67 expression, A4 cluster remained significantly associated with overall survival (Table EV4). Compared with A4-low tumors, patients with A4-high tumors exhibited significantly improved survival (HR = 0.13, 95% CI: 0.02–0.77, *P* = 0.025), whereas A4-intermediate tumors showed a similar trend that did not reach statistical significance (HR = 0.25, 95% CI: 0.06–1.03, *P* = 0.055). These findings indicate that androgen-associated gene expression could capture biologically meaningful variation beyond conventional subtype classification and may contribute to additional clinically relevant information, particularly within Luminal A tumors.

### Single-nuclear RNA sequencing (snRNA-seq) supports the A4-dependent bulk transcriptome discovery

The bulk RNA seq correlated the higher intratumoral A4 level-dependent transcriptome to better BC clinical outcome, which corresponded to more luminal subtype BC, lower proliferation index, and better survival. To confirm those findings at the single-cell level and to determine the cellular characteristics, snRNA seq was performed. Three patient specimens, two A4 high and one A4 low, were selected for this analysis. All three specimens were ER+ve, PR+ve, and AR+ve. The A4 low and one A4 high specimens were HER2-ve, PR+ve, and AR+ve. All three specimens had comparable intratumoral estradiol (1.56-2.57 ng/g tissue). The A4 low specimen contained 1.6 ng/g A4 and 6.9 ng/g DHEA, while the two A4 high specimens contained 9.2 ng/g and 13.4 ng/g A4 and 70.29 and 19.03 ng/g DHEA, clearly segregating the specimens into the two categories based on intratumoral androgens.

**Figure EV4:**
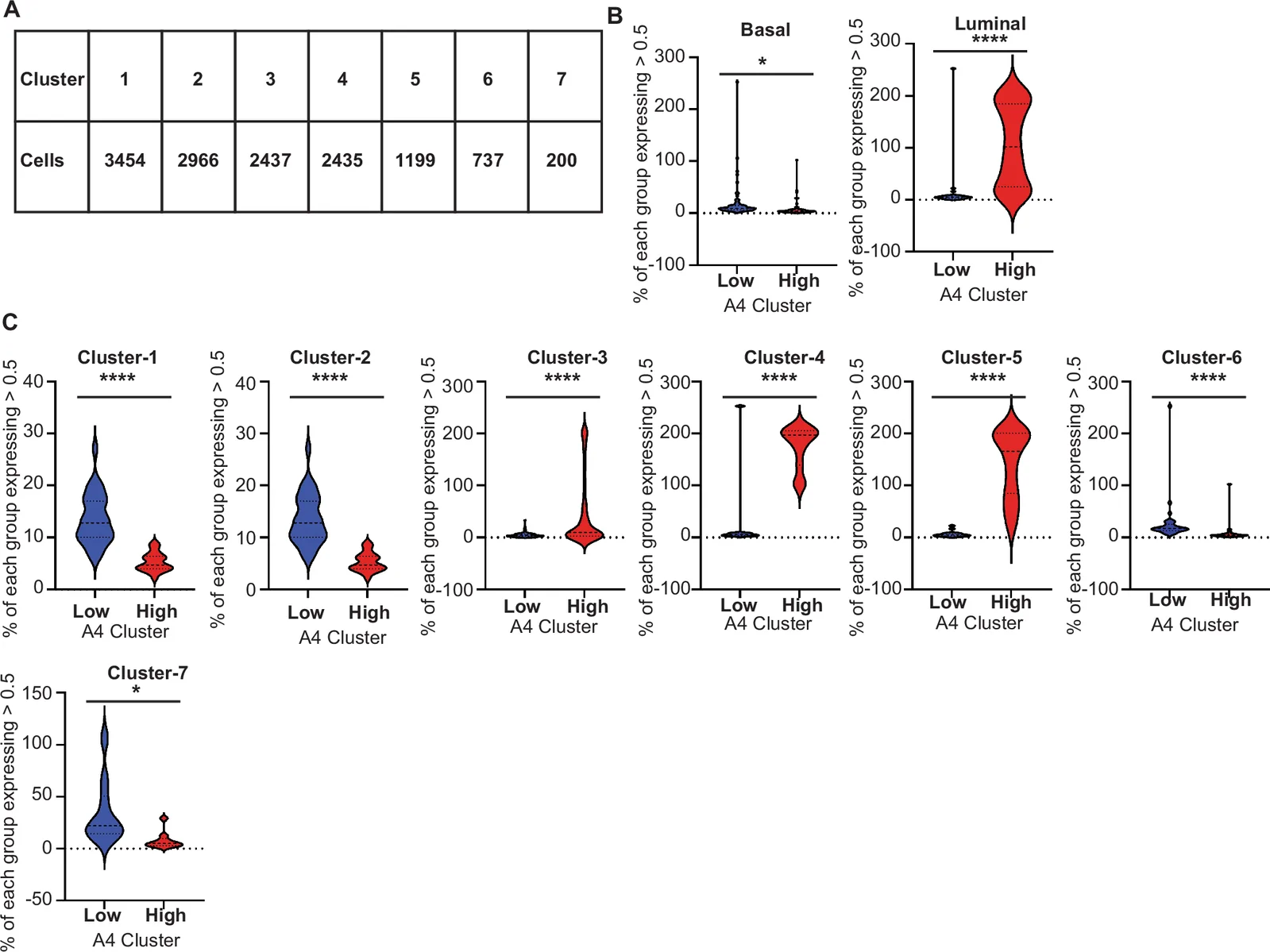
Single-nucleotide RNA sequencing. (**A**) Number of cells in each cluster. (**B**) Violin plot for basal and luminal cells signature. (**C**) Violin plots for all seven cluster signatures. *P < 0.05; **P < 0.01; ***P < 0.001; ****P < 0.0001. A4 low *n* = 7357 nuclei and A4 high *n* = 6071 nuclei. Data were analyzed by Student’s *t* test (*n* = 3). Dashed horizontal lines within the violin plots indicate the median and interquartile range.

**Figure 5:**
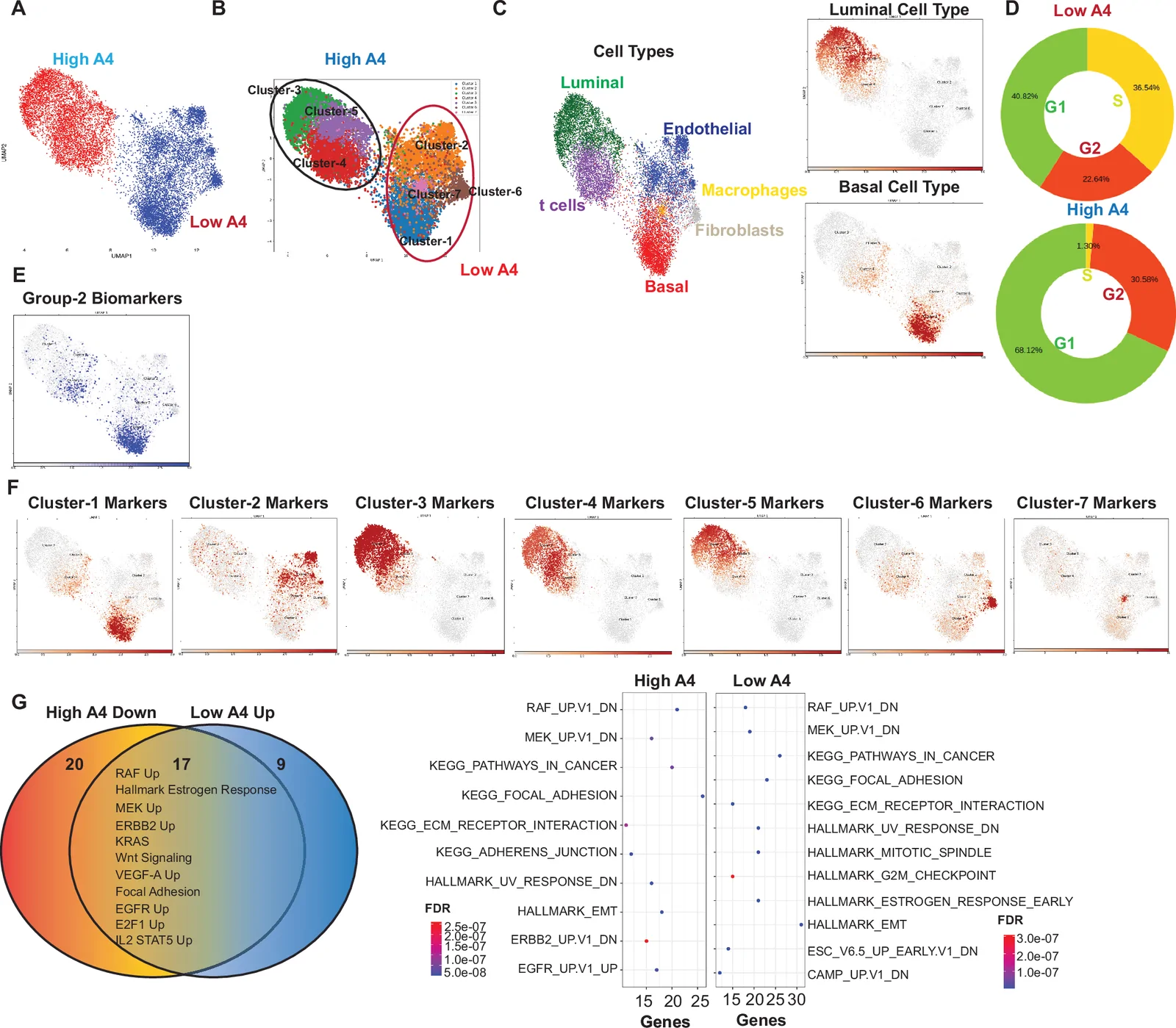
Single-nucleus RNA seq (snRNA seq) separates A4 high and low cells. Two A4 high and one A4 low patient specimens were processed for snRNA seq. About 10,000 nuclei were isolated and sequenced (A4 low *n* = 7357 nuclei and A4 high *n* = 6071 nuclei). (**A**) UMAP of nuclei from A4 high and low samples. (**B**) UMAP showing seven clusters, clusters 1, 2, 6, and 7 belonging to A4 low, and clusters 3, 4, and 5 belonging to A4 high specimens. (**C**) Cell type gene expression signatures were used to determine the enrichment of cells in each cluster, shown as Umap. Basal and luminal cell signature enrichment in A4 low and high samples, respectively, is shown as Umap. (**D**) The pie chart represents the distribution of cell cycle phases in A4 low and high specimens. The percent cell cycle distribution is shown. (**E**) Genes from cluster 2 in Fig. 2C that were high in A4 low samples were used to create an Umap. (**F**) Genes from different clusters are shown as Umap. (**G**) Venn diagram showing the pathways obtained from Gene Set Enrichment Analysis. Pathways increased in the low A4 specimen, and pathways decreased in the high A4 specimen; common pathways in both (up in low A4 and down in high A4) are represented. A4 androstenedione, FDR false discovery rate.

A4 low specimens had 7357 nuclei, while A4 high specimens contained 6071 nuclei sequenced (Fig. EV4A). Umap clearly showed a distinct separation of A4 high and low cells with no overlap (Fig. 5A). Low A4 specimen cells formed four clusters (cluster 1, 2, 6, and 7) while high A4 specimen cells formed three clusters (clusters 3, 4, and 5) (Fig. 5B). The number of cells in each cluster is shown in Fig. EV4A. Evaluating the signatures for each cell type provided distinct type of cells in low A4 and high A4 specimens (Fig. 5C). Low A4 cells were enriched for endothelial cells, basal cells, macrophages (small proportion), and fibroblasts, while high A4 cells were enriched for luminal cells and T cells (Fig. 5C). The luminal cells are typically ER+ve PR+ve, and contribute to better prognosis and response to treatment, while basal cells are ER-ve and are aggressive cell types that lack any therapeutic targets (Malzahn et al, 1998; Oakes et al, 2014; Perou et al, 2000). This cell type identification (Figs. EV4B and 5C) agrees with the ER+ve and TNBC subtypes identified in the bulk RNA seq (Fig. 2C–G). The A4 low specimens are also enriched for fibroblasts and endothelial cells, which are components of the stromal compartment (Mao et al, 2013). The fibroblasts, such as cancer-associated fibroblasts, support a tumorigenic microenvironment (Mao et al, 2013). The endothelial cells that are responsible for the construction of blood vessels promote angiogenesis, invasion, and migration (Longatto Filho et al, 2010). The fibroblasts and endothelial cells form a significant part of the tumor microenvironment. Endothelial cells also play an important role in breast epithelial growth and branching, and stimulate the growth of cancer-derived cells (Ingthorsson et al, 2010; Sigurdsson et al, 2006). Similarly, macrophages play an important role in angiogenesis and blood vessel formation (Leek et al, 1996; Lewis and Pollard, 2006).

Another major cell type found in A4 high specimens is T cells. T cells play a critical role in immune responses in BC (Kos and de Visser, 2021). They are involved in anti-tumor immunity. High levels of CD8+ve T cells through PD-1/PD-L1 inhibition restore the anti-tumor activities of T cells (Tietscher et al, 2023). The cell type distribution and characteristics were reflected in the cell cycle phase distribution of A4 high and low specimens (Fig. 5D). While A4 low specimen comprised of 41% cells in G1 phase, 37% cells in S phase, and 23% cells in G2 phase, A4 high specimens contained cells that were distributed at 68% in G1 phase, 1.3% is S phase, and 31% in G2 phase of the cell cycle.

To validate the 1388-gene signature (Fig. 2C) in sRNA seq data, the genes that are increased in A4 low specimens (gene signature 2) were loaded in snRNA seq data (Fig. 5E). The output, as shown in the Umap, matched the bulk RNA seq heatmap with the genes highly enriched in the basal cell population (cluster 1) with minimum expression in A4 high population. No major crossover was observed in the signature between the A4 low and high specimens. Similarly, the gene signature for each cluster plotted in Umap showed limited crossover between clusters (Figs. 5F and EV4C).

The A4 low and high gene signatures from snRNA seq were used to determine the enriched pathways using GSEA hallmark oncogenic pathways (Fig. 5G). High A4 gene signature exhibited 37 statistically significant pathways, all of which were downregulated. Low A4 gene signature is associated with 26 pathways, with all of them upregulated. All these pathways are oncogenic, suggesting the cell signature of A4 high corresponds to a lower oncogenic signal. Seventeen pathways were shared between A4 high and low cells (regulated in opposite directions). Interestingly, the pathways (RAF, MEK, KRAS, and others) are primarily enriched, suggesting a unified control of the oncogenic MAPK pathway. Collectively, the snRNA seq data are concordant with the bulk RNA seq and provide additional information that facilitates the interpretation that BC cells with a high A4 signature correspond to a favorable molecular profile and disease prognosis. The snRNA seq data were derived from two A4-high and one A4-low ER-positive, PR-positive, and AR-positive specimens, and hence the outcome on immune infiltration, stromal composition, and pathway alteration is exploratory and illustrative rather than definitive, and these outcomes need to be validated in a larger patient specimen cohort.

### Preclinical validation of A4 effects on BC characteristics

**Figure 6:**
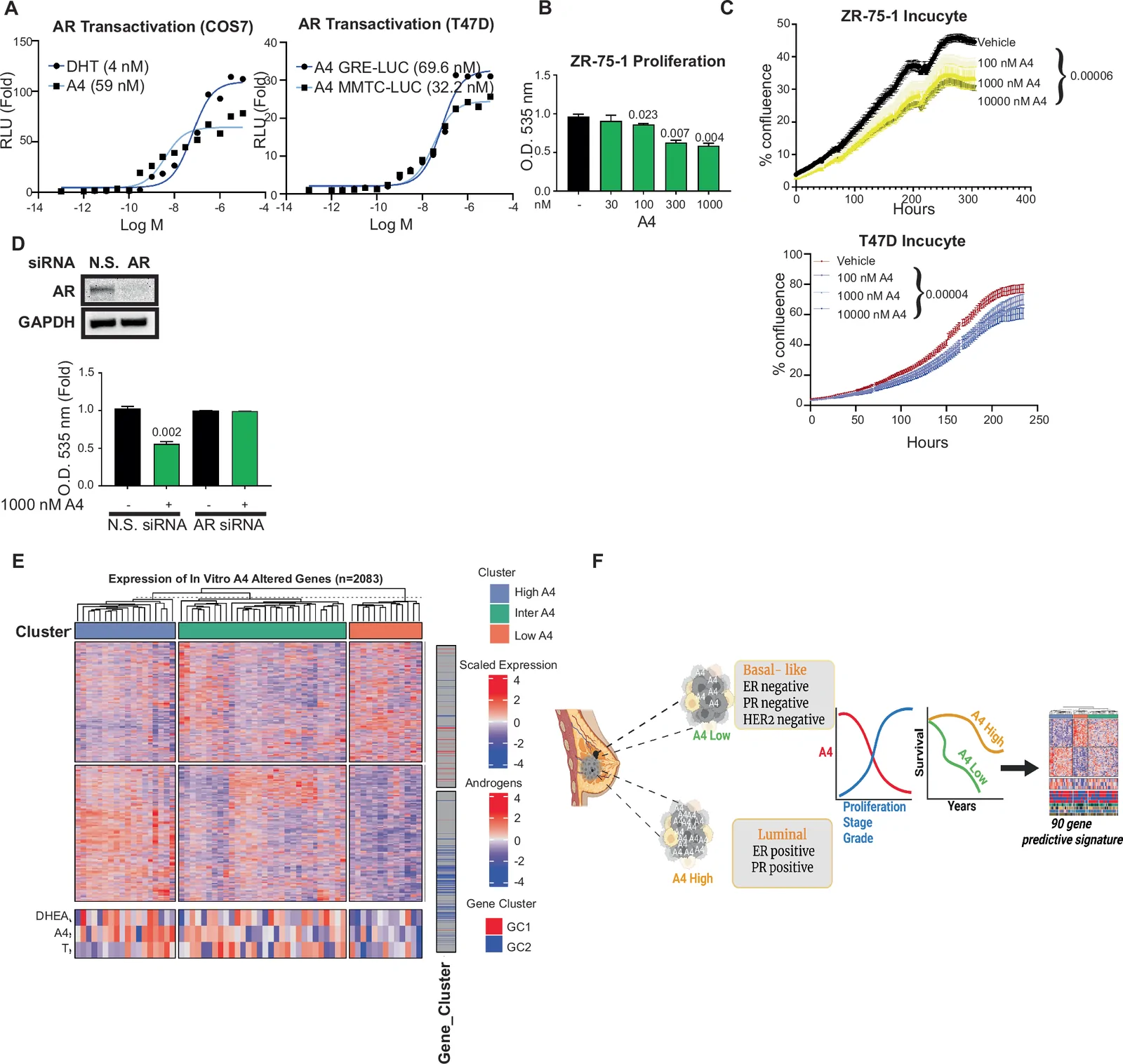
Preclinical validation of clinical results. (**A**) A4 activates AR: AR transactivation study was performed in COS-7 cells transfected with 0.25 µg GRE-LUC, 10 ng CMV-renilla LUC, and 50 ng pCR3.1 hAR using lipofectamine. Cells were treated 24 h after transfection, and a luciferase assay was performed 48 h after transfection. Firefly values were normalized to Renilla luciferase numbers. Transactivation studies in T47D cells were performed with MMTV-LUC and GRE-LUC as indicated above for COS-7 cells. Each transactivation experiment was performed with a nine-point dose response, and the experiment was repeated three times independently. (**B**) A4 inhibits BC cell proliferation: ZR-75-1 cells were plated in a 96-well plate and treated with the indicated doses of A4 (*n* = 4/treatment). Cells were fixed and stained for SRB 14 days after treatment initiation. Data were analyzed by One-way ANOVA followed by the Tukey post-hoc test. Each experiment was performed with *n* = 4 biological replicates, and the experiment was repeated two times independently. (**C**) A4 inhibits the growth of BC cell lines: ZR-75-1 or T47D cells were treated as indicated with a dose response of A4, and the confluence was imaged using IncuCyte over a period of 14 days. Data were analyzed by two-way ANOVA followed by a post-hoc test. The data were also analyzed by the area under the curve. Each experiment was performed with *n* = 4 biological replicates, and the experiment was repeated two independent times. (**D**) A4 effects are mediated through AR: T47D cells were transfected with non-specific siRNA (N.S.) or AR siRNA, and proliferation in the presence of A4 was measured by SRB assay (*n* = 4). Data were analyzed by one-way ANOVA. The experiment was performed three times independently. (**E**) In vitro validation of the gene signature and RNA sequencing: ZR-75-1 cells (*n* = 3–4) were plated in growth medium and treated for 24 h or 14 days with vehicle or 1 µM A4. Cells were harvested, RNA extracted, and sequenced. The scaled gene expression of 2083 genes across the clinical samples (*n* = 61 patients) is displayed with the previously identified patient clusters: High-A4 (blue), Inter-A4 (green), and Low-A4 (orange). Genes (rows) were grouped into two main clusters using hierarchical clustering. The previously defined gene clusters, GC1 and GC2, are annotated in red and blue, respectively, while genes not assigned to either cluster are shown in gray. (**F**) Graphical summary: Data are represented as mean ± SE. Source data are available online for this figure.

Considering that an A4-dependent signature is associated with favorable clinical variables, we performed preclinical experiments to test whether A4 has direct growth-inhibitory activity in ER+ve/AR+ve BC cell lines. We used two ER-positive AR-positive BC cell lines, T47D and ZR-75-1, for these validation experiments. AR transactivation studies in both COS7 and T47D cells demonstrated that A4 activated AR (Fig. 6A). A4 was tested in a proliferation assay in the ER and AR+ve BC cell line, ZR-75-1. A4 dose-dependently inhibited the proliferation of ZR-75-1 cells with ~40% inhibition (Fig. 6B).

A4 dose-dependently inhibited the growth of ZR-75-1 and T47D cells over a 14-day period, as measured by IncuCyte confluence (Fig. 6C). Using repeated-measures two-way ANOVA that accounts for the longitudinal, same-well structure of the data, we observed a significant main effect of treatment × time interaction (*P* = 0.00006). This result was corroborated by an independent, replicate-level AUC analysis, confirming that the effect was not dependent on the specific repeated-measures modeling approach. Since A4 is not a cytotoxic agent, the magnitude of growth inhibition observed (~40% relative to vehicle) is consistent with a cytostatic rather than cytotoxic mechanism, in line with effect sizes reported for other cytostatic compounds in comparable long-term proliferation assays.

The ZR-75-1 vehicle-treated growth curve showed a non-monotonic pattern, including a late increase in confluence (occurring after the day-9 medium change, which we attribute to renewed nutrient availability following a confluence-associated growth plateau; this pattern was reproducible across independent experiments and was specific to the vehicle condition, likely reflecting medium exhaustion between the scheduled every third-day medium changes in this cell line). This pattern did not alter the outcome of either the repeated-measures model or the AUC-based comparison, both of which incorporate the full time-course rather than a single endpoint measurement.

To determine if A4’s effect on proliferation is mediated by its receptor, AR, AR was knocked out in AR and ER +ve T47D cells using siRNA, and proliferation was measured by SRB assay (Fig. 6D). Knocking out AR blocked A4’s effect on cell proliferation, suggesting that A4’s effects in BC are promoted by AR.

### In vitro validation of gene expression

To examine whether the clinical A4-associated gene signature could be recapitulated by direct A4 treatment in vitro, we treated ER and AR-positive ZR-75-1 BC cell line with 1 µM A4 for 24 h and for 14 days (*n* = 4/group) (Fig. 6F). The dose was chosen based on the effect of A4 on cell proliferation. The rationale for two time points was to determine if the A4-correlated clinical gene signature is just the result of transcriptional regulation by A4 or if the signature is the result of the effect on proliferation.

A cutoff of 1.5-fold and FDR of 0.05 resulted only in 41 genes regulated by A4 at 24 h. However, by 14 days, A4 significantly altered the expression of 3292 genes, suggesting that all genes altered by A4 are either due to long-term exposure or a result of an effect on proliferation.

The A4-altered genes identified in the in vitro experiment were further investigated using gene expression data from clinical samples (*n* = 61). Of the 3292 A4-altered genes, 2083 overlapped with the clinical gene expression dataset. To assess the expression patterns of these genes across patients with varying A4 levels, we compared their expression profiles using a heatmap, grouping samples by the previously identified clusters: High-A4, Inter-A4, and Low-A4.

The 2083 genes were newly classified into two clusters through hierarchical clustering. As expected, differential expression patterns were observed between the High-A4 and Low-A4 groups, consistent with the patterns previously identified using 1388 genes. The first gene cluster (*n* = 977) exhibited low expression in the High-A4 group and high expression in the Low-A4 group, while the second cluster (*n* = 1106) showed the opposite pattern. These clusters correspond to gene clusters, GC1 and GC2, respectively, as defined by the original set of 1388 genes (Fig. 2C). This strong correlation highlights the consistency between the A4-altered genes identified through the in vitro experiment and their expression patterns relative to A4 levels in clinical data.

Gene set enrichment analysis of the genes showed that androgen response is one of the hallmark pathways enriched in the A4-treated cells. In addition, estrogen-responsive pathways are enriched in vehicle-treated cells, concordant with our earlier publications that showed treatment of ER-positive BC tumors with androgens inhibits the estrogen pathway(Asemota et al, 2024; Ponnusamy et al, 2019a).

## Discussion

The novel approach to determine the intratumoral androgen levels combined with transcriptome demonstrates that an A4-associated gene signature is associated with lower tumor stage and grade, lower proliferation, better survival, and a lower chance of developing TNBC. Our data also suggests that this A4-associated signature marks a differentiated low-proliferative tumor state that is linked to higher intratumoral A4 levels. The training set data were validated using a large TCGA BC dataset and our internal validation cohort. Although the A4 level measurement in the validation cohort was variable, the A4-correlated gene expression signature-derived clusters obtained in the primary cohort matched well with this cohort and showed the same trend in proliferation. Hence, the genes distinguishing the A4-low and A4-high clusters may hold predictive value for grade, stage, and survival, pending validation in independent cohorts, and this could potentially eliminate some of the bias in immunohistochemical staining and pathological evaluation. Moreover, gene expression has a high dynamic range that could be used to categorize the tumors if we can define a range for the expression. The results are depicted in a model presented in Fig. 6G.

Although we expected a meaningful signature with testosterone, the result with A4 was surprising. Results from previous studies using serum hormone levels were confounding, where a positive correlation between serum androgen levels and increased BC risk was observed (Albers et al, 2024). An interesting observation made in preclinical models based on clinical observation was that aromatase inhibitors, exemestane and formestane, can be metabolized to sterol androgen metabolites by BC cells, causing an inhibition of cell proliferation (Gao et al, 2019). This needs to be determined using our methodology of determining intratumoral androgen levels in patients treated with aromatase inhibitors.

Various clinical studies indirectly predicted that A4 is associated with higher BC risk (Kaaks et al, 2005; Manjer et al, 2003). These studies derived the interpretation based on serum A4 levels and the predicted conversion of A4 to estrone and estradiol (Barone et al, 1979; Manjer et al, 2003). However, in our study, we did not find evidence that higher A4 levels converted to estrone, leading to increased proliferation. Alternatively, a recent work from Dr. McDonnell’s laboratory demonstrated that low intratumoral androgen levels in prostate cancer function through AR monomer that activates the mTOR signaling pathway and increases prostate cancer cellular proliferation, while high androgen levels function through AR dimer that results in c-Myc oncogene inhibition and inhibition of proliferation (Safi et al, 2024). This mechanistic study explains the biphasic androgen function in prostate cancer that have demonstrated that treatment with supraphysiological doses of androgens will result in an inhibition of tumor growth (Chatterjee et al, 2019). It is possible that A4 in BC functions through a related mechanism. The observation that high-A4 specimens show decreased mTOR and c-Myc oncogenic signaling (Fig. 2B) is consistent with, though does not establish, the mechanism described in prostate cancer.

The diagnostic and prognostic landscape of BC is not definitive. Currently, BC is diagnosed by a mammogram. Tumors are classified based on ER, PR, and HER2 expression, and the proliferation rate is determined by ki67. Seven different gene signatures that range from providing an early prediction to treatment response are available (Cardoso et al, 2016; Cuzick et al, 2011; Paik et al, 2004; Parker et al, 2009; Ring et al, 2006; Sestak et al, 2020; Sgroi et al, 2013). Of these, the commonly used signature is the 21-gene panel Oncotype DX. None of these signatures is directed towards a single pathway. The ideal predictive signature will have to be directed towards a well-validated inactivated tumor suppressor or activated oncogenic pathway that can serve a spectrum from early prediction to treatment response, and even survival. We believe that one of the most widely expressed hormone receptors in BC, AR, and its most abundant circulating ligand androgen have potential to serve as both therapeutic and prognostic in BC. On this front, the 90 gene signature that correlates with intratumoral A4 has the potential to serve as a reliable predictive biomarker that can be used to predict future recurrence, survival, and other characteristics. However, additional validation in independent cohorts will be necessary before its clinical utility can be established.

Some of the limitations of this work include a smaller cohort size, tumor purity, stromal fraction, fibroblast enrichment, endothelial cell population, and intratumoral steroidogenic enzyme expression, all of which have the potential to alter the predictive properties of the A4-dependent signature and the disease outcome. The snRNA seq was conducted with a limited number of specimens that are not representative of the large, highly heterogeneous BC specimens. These mechanistic data should be used as guidance until further validation with a large cohort is available. Additionally, longitudinal specimen utilization will help in understanding the relationship of this signature to the progression of the disease. Further validation using a potentially pure cellular population and extensive quantification of enzymes and other hormones will facilitate the confirmation of the findings in this manuscript. Overall, the study sheds light on the potential prognostic association between intratumoral androgens and BC outcome. If this gene signature in A4 high cluster validates in a large cohort and provides a reliable gene signature, the signature will potentially have the necessary characteristics to be translated into the clinic.

## Methods

**Table:** Reagents and tools table

Reagent/resource	Reference or source	Identifier or catalog number
**Experimental models**		
T47D	ATCC	HTB-133
ZR-75-1	ATCC	CRL-1500
COS-7	ATCC	CRL-1651
**Recombinant DNA**		
GRE-LUC	Yepuru et al, 2013; Narayanan et al, 2008	
MMTV-LUC	Gift from Drs. Irina Agoulnik and Nancy L. Weigel (Auboeuf et al, 2002)	
CMV Renilla-LUC	Yepuru et al, 2013; Narayanan et al, 2008	
CMV-hAR	Yepuru et al, 2013; Narayanan et al, 2008	
**Antibodies**		
Androgen Receptor	Cell Signaling	5153
GAPDH	Sigma	G8795
Steroid Receptor Coactivator-1 (SRC-1)	Cell Signaling	2191
SRC-3	Cell Signaling	2126
CBP	Cell Signaling	7389
**Oligonucleotides and other sequence-based reagents**		
Silencer Select AR siRNA	Thermo Fisher	4390824 (Assay ID: s1538)
Silencer Select non-specific snRNA	Thermo Fisher	4390843
**Chemicals, enzymes, and other reagents**		
Dihydrotestosterone	Sigma	A8380
Androstenedione	Sigma	SML2364
Luciferase assay	Goldbio	I-925-1000
Transfection reagent	Life Technologies	18-324-012
**Other**		
Cell line authentication	Genetica cell line testing laboratory, Burlington, NC	

### Ethics approval and consent to participate

This study was approved by the University of Tennessee Health Science Center (UTHSC) institutional review board (IRB) (IRB protocol numbers 14-03113-XP and 20-07769-XP). The studies conformed to the principles set out in the WMA Declaration of Helsinki and the Department of Health and Human Services Belmont Report. All methods/study procedures were carried out in accordance with the relevant guidelines and regulations described in the Declaration of Helsinki. All participants were informed about the purpose of the study, assured of confidentiality, and provided written consent prior to participation. Participation was voluntary, and respondents could withdraw at any time without consequence.

BC patient specimens were collected under protocols approved by the UTHSC IRB (IRB protocol numbers 14-03113-XP and 20-07769-XP). Seventy-three specimens were collected for intratumoral hormone measurements and transcriptomics. Since only 61 specimens’ transcriptomic data met the quality control benchmark, 61 specimens’ data were used for analyses. For the validation cohort, 88 specimens were collected. Transcriptome data from only 77 specimens met the benchmark, and hence, the validation cohort contained data from 77 patient specimens.

### Cell culture

T47D, ZR-75-1, and COS-7 cells were obtained from American Type Culture Collection (ATCC, Manassas, VA). Cells were cultured in accordance with ATCC recommendations and were authenticated by short terminal DNA repeat assay (Genetica cell line testing laboratory). Cells were frequently tested for mycoplasma infection.

### Western blot

Western blots were performed as previously described (Asemota et al, 2023; Ponnusamy et al, 2019a) with AR (Cell Signaling, Danvers, MA) antibody and normalized to GAPDH (Sigma, St. Louis, MO; G8795). Briefly, protein extracts were prepared by three freeze-thaw cycles in lysis buffer (50 mM Tris-HCl, 150 mM NaCl, and 1% Triton-100 adjusted to pH 7.4), and the cell debris was removed by centrifugation at 14,000 rpm for 10 min at 4 °C. Protein concentration was measured by BCA assay (bicinchoninic acid). Equal amounts of protein were loaded on an SDS-PAGE gel and fractionated. Proteins were transferred to a nitrocellulose membrane, and a western blot was performed with AR antibody (dilution of 1:1000) and horseradish peroxidase (HRP)-tagged anti-rabbit secondary antibody (1:3000 dilution). The membrane was stripped and reprobed with GAPDH antibody (1:3000 dilution) and HRP-tagged anti-mouse secondary antibody (1:5000 dilution). The membranes were developed by enhanced chemiluminescence (ECL) reagent.

### Sulforhodamine Blue (SRB) assay

Cells were plated in growth medium in 96-well plates and treated for the duration indicated in the figures. Medium was changed every third day. Cell viability was measured by SRB assay (Asemota et al, 2023; Ponnusamy et al, 2017; Ponnusamy et al, 2019b). Data were analyzed by one-way ANOVA followed by the Tukey post-hoc test.

### IncuCyte growth assay

Cells were plated in 96-well plates in growth medium at varying densities, with *n* = 4 replicate wells per condition within each experiment. Cells were treated with vehicle or A4 at the indicated concentrations. Plates were incubated in an IncuCyte live-cell imaging system and imaged every 4 h for confluence. Growth medium containing the corresponding treatment was refreshed every third day for the duration of the assay. Each experiment was independently repeated at least two times (biological replicates). Well-level (technical replicate) confluence values were averaged within each biological replicate prior to statistical analysis.

Longitudinal confluence data were analyzed using a repeated-measures two-way ANOVA (treatment × time, with biological replicate as the repeated unit), followed by post-hoc comparisons at each time point with correction for multiple comparisons. The *P* value reported in the results corresponds to the treatment × time interaction. A secondary replicate-level analysis that does not depend on the repeated-measures correlation structure, area under the confluence curve (AUC) was calculated for each biological replicate using the trapezoidal method, and AUC values were compared between treatment and vehicle groups using the Mann–Whitney *U* test. Statistical significance was set at *P* < 0.05. Analyses were performed using GraphPad Prism.

### AR transactivation

AR transactivation studies were performed as described before in COS-7 cells transfected with GRE-LUC, CMV Renilla LUC, and hAR using Lipofectamine reagent (Life Technologies, Carlsbad, CA) (Ponnusamy et al, 2017; Ponnusamy et al, 2019b). Transactivation studies with endogenous AR were performed in T47D cells transfected with GRE-LUC and MMTV-LUC and CMV-renilla LUC (Auboeuf et al, 2002; Narayanan et al, 2008; Yepuru et al, 2013).

### RNA sequencing (RNA-seq)

RNA was extracted from formalin-fixed paraffin-embedded (FFPE) specimens using the Qiagen RNA extraction kit, and RNA integrity and quantity were assessed using Agilent Bioanalyzer. mRNA-seq libraries for the Illumina platform were generated and sequenced at Azenta (Burlington, MA) using the Illumina HiSeq following the manufacturer’s protocol. Sequencing with RNA from cell culture was performed in the UTHSC molecular resources center (MRC) using an Illumina sequencer.

### RNA-seq analysis

Analysis was performed as previously described(Asemota et al, 2023). Fastq files that passed quality control processing using FastQC (v0.11.8) were first aligned to the human transcriptome (hg38 genome build, GENCODE v40 transcript annotation) using STAR (v2.7.0) and then sorted with SAMtools (v1.9). Salmon (v0.13.1) was then used for transcript quantification, and gene-level counts were used for data analysis in R (v4.1.2). Read counts were loaded from salmon quant files using tximport (v1.6.1), and differential gene expression analysis was performed using DESeq2 (v1.28.1). An adjusted *P* value <0.1 was used to determine significantly differentially expressed genes (DEGs) from each sample group. Read counts were normalized for downstream analyses and visualization using the variance stabilization transformation (VST) from DESeq2. Heatmaps representing VST normalized and scaled expression values were generated with the ComplexHeatmap package (v2.4.3), where rows and/or columns were clustered via the “Pearson” distance method.

### Patient specimen collection

We collected 73 fresh BrCa specimens from surgical suites. Patient details are provided in Table EV1. The patient characteristics by ER status are represented in Dataset EV2. The specimens were accompanied by personal information-redacted electronic patient records. The data obtained from the 73 patient specimens were validated using a validation set that comprised 88 specimens. Patient characteristics of this validation set are provided in Fig. EV2A (data represented by ER status).

### Immunohistochemistry (IHC)

Formalin-fixed paraffin-embedded blocks were sectioned at a thickness of 3 µm and mounted on glass slides. The slides were stained for ER (Leica Biosystems, Deer Park, IL; Clone 6F11), AR (Cell Signaling #5153), PR (Roche Diagnostics, Germany), HER2 (DAKO, Copenhagen, Denmark; Rabbit polyclonal), and Ki67 (DAKO; M7240) using an IHC protocol previously described (Asemota et al, 2024; Ponnusamy et al, 2019a). All patient specimens were stained simultaneously in a central laboratory to avoid batch effects, and the staining was scored by two pathologists.

### Intratumoral hormone measurement

Intratumoral hormone levels were quantified based on previously described and validated methods (Bhardwaj et al, 2023; Han et al, 2021). Gas chromatography-mass spectrometry (GC-MS)-based steroid profiling was performed using a Shimadzu GC 2010 Plus gas chromatograph interfaced with a triple-quadrupole Shimadzu GCMS-TQ8050 (Shimadzu Co., Kyoto, Japan). In brief, 5 mg of BC specimens were cut and added to 1 mL 0.2 M phosphate buffer (pH 7.2) and 20 μL of a mixture of internal standards (d8-17a-hydroxyprogesterone, 1 μg/mL; d6-DHEA and d9-progesterone, 0.5 μg/mL; d3-testosterone and d4-cortisol, 0.25 μg/mL; d4-17β-estradiol, 0.2 μg/mL). After pulverizing the tissues twice with TissueLyser II (Qiagen; Hilden, Germany) for 10 min at 25 Hz with three zirconia beads (3.0 mm I.D., Toray Industries, Tokyo, Japan), the samples were centrifuged for 5 min at 12,000 rpm, and the supernatant was collected. The samples were then loaded on a preconditioned Oasis HLB SPE cartridge. The cartridge was washed twice with 1 mL of water and eluted with 2 mL of methanol and 2 mL of 90% methanol. The combined eluates were evaporated under nitrogen at 40 °C. Then, 1 mL of 0.2 M acetate buffer (pH 5.2) was added for additional extraction. After two extractions with 2.5 mL of methyl tert-butyl ether, the organic solvent was collected, evaporated under nitrogen at 35 °C, and further dried in an amber vacuum desiccator with P2O5/KOH for at least 30 min. Finally, the dried residues were derivatized in 40 μL of MSTFA/NH4I/DTE (500:4:2, v/w/w) for 20 min at 60 °C, and 2 μL of the resulting mixture was analyzed with GC-MS in the collision-induced dissociation mode.

#### Method validation and quantification limits

Method validation was performed according to the US Food and Drug Administration 2018 guideline for bioanalytical method validation (validation [US Food and Drug Administration. (2018) *Guidance for Industry: Bioanalytical method validation*, No. FDA-2013-D-1020, 1–41]). The following validation parameters were previously established using this assay (Han et al, 2021) and were confirmed to be applicable to the current cohort by inclusion of quality-control samples at defined concentrations in each analytical run. The overall precision (%CV) and accuracy (%bias) across the analytical range were 0.8–19.9% and 69.9–111.1%, respectively. Per-analyte lower limits of quantification (LLOQ), defined as the lowest concentration quantifiable with acceptable accuracy, were 0.1 ng/ml (Han et al, 2021) for all the analytes. Per-analyte lower limits of detection (LLOD), defined as the lowest concentration producing a signal-to-noise ratio of ~3, were 0.028 ng/ml for the analytes. As noted in the Results, DHT levels in a majority of specimens fell below the LLOQ and were therefore excluded from downstream analyses.

#### Matrix effects

Matrix effects were controlled using two complementary strategies. First, deuterated internal standards specific to each steroid class (listed above) were spiked into each tissue sample prior to homogenization and extraction; because internal standards are subject to the same ion suppression/enhancement as the corresponding native analyte, normalizing analyte signal to internal standard signal corrects for sample-to-sample differences in ionization efficiency. Second, calibration curves were prepared in matrix-matched surrogate tissue (rather than solvent-only standards) so that the calibration itself reflects the same matrix background present in patient specimens; each analyte was quantified against its corresponding isotope-labeled internal standard using these matrix-matched curves.

#### Tissue-processing variability

Several steps were taken to minimize and account for pre-analytical and processing variability across specimens. Tissue specimens were collected in the surgical suite, trimmed of adjacent fatty tissue, and snap-frozen within 30 min of excision to limit degradation prior to freezing. Homogenization was performed using a fixed protocol (TissueLyser II, 25 Hz, 10 min, two cycles) identical across all specimens to minimize inter-sample variability in extraction efficiency due to inconsistent tissue disruption. To account for differences in specimen size and cellular composition, hormone concentrations were normalized to tissue wet weight (ng/g tissue) rather than reported as absolute quantities. Internal standards, spiked prior to extraction, additionally correct for sample-to-sample variability in extraction and derivatization efficiency downstream of tissue processing. Steroid concentrations were log-transformed and standardized to z-scores prior to downstream statistical analyses to reduce the influence of any residual technical variability on cluster assignment.

### Gene expression pre-processing

Starting with the normalized gene expression for the protein-coding genes (*n* = 19,438), genes with low expression or low variability were filtered out from the downstream analysis. Specifically, genes with low median (< 25% percentile) or low median absolute deviation (< 25% percentile) were excluded (*n* = 5980), resulting in a final set of *n* = 13,458 protein-coding genes. Of the 161 patient specimens collected across the two cohorts (73 for the clinical cohort and 88 for the validation cohort), 138 samples had usable gene expression data and were included in the transcriptomic analyses (*n* = 61 for the clinical cohort and *n* = 77 for the validation cohort).

### Single-nucleus RNA sequencing (snRNA seq) analysis

Fresh frozen specimens were used for snRNA seq experiments (*n* = 2 from A4 high cohort and *n* = 1 from A4 low cohort). Sample processing, single nuclei isolation, and sequencing were performed at Azenta as described previously (Asemota et al, 2024). Each fastq was subjected to quality control and correction using FASTQC and FASTX trimmer. The trimmed fastq was aligned to the Hg38 reference using RNA-STAR (Dobin et al, 2013). The SAM files were mined for the alignment of primary counts and cell barcodes using Illumina Cell Ranger. Samples contain 10,000 cells before filtering. The counts were normalized, log2 transformed, and filtered for low expression using Seurat (Hao et al, 2021). Samples have 8500 cells after filtering. The normalized data matrix was used to generate UMAP and clustering. The parameters for clustering and UMAP were n_epochs = 1000, resolution = 0.2, and dims = 5. Clustering was organized using metadata, and unsupervised clustering DeSeq2 was used to identify genes with log2 fold change >= 0.58 and *P* value < 0.05 between clusters. GSEA gene ontology and cell type analysis were performed on the clusters to further investigate cell type populations and transcript effects. Web-based cell-type-specific enrichment analysis of genes was used to perform cell type analysis (Dai et al, 2022). Various marker sets were used to visualize Colormaps for scatterplots, including Basal type, Luminal type, Biomarker-associated clusters, expression of differential genes for each cluster, AR-related genes, and ER-related genes (Dataset EV4).

### Correlation analysis between hormones and gene expression (original clinical cohort only, *n* = 61)

This was an open study, and no blinding was utilized for analysis. Patient's personal information was redacted and withheld. The analysis was performed in an unblinded manner. Randomization and blinding were not applicable to analyses of existing clinical and genomic datasets. To select genes that are highly correlated with hormones, the correlation between genes and hormone levels was obtained. For this analysis, we only focused on the samples in the original clinical cohort. Out of the total 73 samples in the original clinical cohort, only 61 samples had usable gene expression profiles and were included for the correlation analysis. For each hormone, Pearson correlation and their *P* values were computed for all 13,458 genes, followed by multiple testing correction using *q* value. For A4, 1388 genes were identified based on *q* value < 0.1 (496 genes with *q* value < 0.05; Dataset EV1).

### Unsupervised molecular subtype detection in the clinical cohort

The 1388 genes that significantly correlated with A4 were used for unsupervised subtype detection by *K*-means clustering. We employed a silhouette plot as well as a consensus matrix to determine the optimal number of clusters using R packages cluster and ConsensusClusterPlus (Wilkerson and Hayes, 2010). A silhouette plot shows the relative distance of how close a sample in one group is to a nearby group, which is indicated by the silhouette width ranging from –1 to 1 (Fig. EV3A). A value close to 1 represents good separation from the neighboring clusters, whereas a negative value implies that the corresponding sample has been assigned to the wrong cluster. The resulting silhouette plot shows a good separation between clusters with no negative values for each sample (Fig. EV3A). In addition, the consensus matrix, a symmetric display of consensus values between sample pairs, indicates consensus levels with dark blue representing high consensus where samples consistently cluster together. The resulting consensus matrix, based on x1000 subsampling iterations, suggests that three clusters (*k* = 3) are a reasonable number of underlying clusters (Fig. EV3B). While a total of two clusters (*k* = 2) also demonstrate good separation, observing the gradual changes in gene expression profiles indicates that *k* = 3 would be more appropriate. This enables the comparison between two extreme clusters, Clusters 1 (low A4 group) vs. 3 (high A4 group), which might have greater clinical relevance.

### Association tests and enrichment analysis

The identified sample clusters were compared with other clinical variables using the Kruskal-Wallis test by ranks for continuous variables (hormones, Ki67, and age) and the Fisher’s exact test for categorical variables (ER, PR, HER2, AR, race, stage, grade, and recurrence). The identified gene clusters were used for Gene Ontology (GO), KEGG/Reactome pathway enrichment, and Hallmark gene set enrichment analysis (GSEA) with the Bioconductor packages msigdbr (https://igordot.github.io/msigdbr/authors.html#citation; Liberzon et al, 2015), biomaRt (Durinck et al, 2009), and clusterProfiler (Yu et al, 2012). The number of genes used as background is 13,458 protein-coding genes, which were the final set of genes consistently used in our analysis.

### Androstenedione-correlated molecular subtype prediction in the TCGA BC dataset

The TCGA BC RNA-seq data along with the corresponding clinical data (*n* = 973) were downloaded from GDC Portal using the TCGAbiolinks Bioconductor R package. For clinical data, we extracted a set of important clinical variables, including race, vital status, age, metastasis, tumor stage, ER status, PR status, and HER2 status. The TCGA BC RNA-seq data were processed by the same normalization technique used for our clinical cohort as described above. To identify the A4-correlated molecular subtypes for the TCGA BC samples, we applied a robust centroid prediction based on the normalized gene expression profile. Among the 20,499 genes available in TCGA BC RNA-seq data, only a subset of genes (*n* = 1231) overlapping with the 1388 A4-correlated genes in the clinical cohort was used for the subtype prediction. Using the centroids obtained from the three clusters identified from the clinical gene expression profile, centroid similarity was measured by Pearson correlation using the 1231 genes, followed by the nearest centroids prediction (Wilkerson et al, 2012).

### Development of a 90-gene predictor for A4

We applied sPLS, a supervised multivariate method designed to handle high-dimensional datasets, to identify a subset of genes most predictive of A4 levels. This method is a variant of the partial least squares (PLS) regression, with the added benefit of sparsity constraints, which facilitates feature selection by shrinking the coefficients of less relevant genes to zero. For the analysis, gene expression data consisting of 13,458 genes from 61 samples were used as input data. The expression data were standardized (mean-centered and scaled to unit variance) prior to analysis to ensure comparability across genes. The A4 levels, which served as the response variable, were also standardized to enable direct comparison of model coefficients. To identify the optimal number of dimensions (components) and genes (features) required for predicting A4 levels, we conducted cross-validation using the mean absolute error (MAE) as the evaluation metric (Fig. EV3C). Specifically, we compared models with one dimension (Component 1) and two dimensions (Components 1 and 2) while varying the number of selected genes (features) (Fig. EV3C). Fivefold cross-validation was used to determine the optimal number of genes in each case. The minimal MAE was achieved with one component and 90 genes, which were selected for subsequent analyses.

Based on the cross-validation results, the final sPLS model was fit to the full dataset, using one component and 90 genes. The sPLS model projects the expression data of the selected genes onto the first component, and the projection is used to predict A4 levels. The sparsity penalty in the model was tuned to maximize feature selection, allowing the identification of genes that contributed most strongly to the prediction of A4 levels.

The performance of the sPLS model was evaluated by comparing the predicted A4 levels to the observed values (Fig. EV3C). Pearson correlation and coefficient of determination were calculated to assess model fit. We also examined the loading values of the selected genes to interpret their contributions to A4 prediction. Genes with higher absolute loading values were considered to have stronger contributions, with positive loadings indicating a positive association with A4 levels, and negative loadings indicating a negative association ().

To further validate the robustness of the model, we assessed the overlap between the 90 genes selected by sPLS and the 1388 genes previously identified as highly correlated with A4 levels through unsupervised analysis. This comparison helped confirm the relevance of the selected gene set in A4 prediction.

All analyses were conducted using SCISSOR (Choi et al, 2021) and “mixOmics” (Rohart et al, 2017) packages in R.

### LOOCV for validating the selected gene set

LOOCV is a cross-validation technique often used to assess the performance of machine learning models, particularly for small datasets. We applied an adapted LOOCV approach to validate the reliability of the selected 90-gene set for predicting A4 levels. In LOOCV, each observation is treated as an individual test case, while the remaining data points are used to train the model. This process is repeated for all observations, meaning that for a dataset with n points, the model is trained and evaluated n times. For each iteration, we focused on one-dimensional prediction using the first component, consistent with our tuning results, but the number of features was re-tuned in every iteration to trace the selected gene set across repeats. In addition, fivefold cross-validation was employed within each iteration to re-estimate the optimal number of genes, providing sufficient randomness through different sample splits to assess whether the gene set was consistently and robustly selected (Fig. EV3D).

### A 90-gene classifier with a modified centroid-based prediction method

The selected 90-gene set was used to predict the A4-low, -inter, and -high clusters by applying a centroid prediction method based on the PAM algorithm (Parker et al, 2023; Tibshirani et al, 2002), utilizing Spearman’s rank correlation as the distance metric. The centroids were derived from the previously identified patient clusters (Fig. 2).

During this analysis, we observed that some samples exhibited low correlations with all three centroids yet were still classified into the A4-low or A4-high clusters despite the weak correlation. This raises concerns about the reliability of the classification, as such samples may not genuinely align with their assigned clusters. This issue is particularly critical for the A4-low and A4-high groups, which represent the two extremes of gene expression profiles. Misclassification into these groups could potentially confound the results and interpretations.

To address this, we modified the standard centroid prediction method to impose stricter criteria for membership in the A4-low and A4-high clusters. Specifically, a sample’s 90-gene expression profile needed to exhibit a Spearman correlation of >0.5 with the corresponding centroid to qualify for inclusion in the A4-low or A4-high clusters. Samples failing to meet this threshold were classified into the A4-inter group, which represents intermediate expression profiles between the two extremes.

We applied this modified centroid prediction method to both the clinical dataset and the TCGA BRCA dataset to ensure robust and reliable classification.

### PAM50 subtype assignment and proliferation score

PAM50 intrinsic subtypes were assigned in the TCGA BRCA cohort using a nearest-centroid approach based on the PAM50 gene set. Gene expression values were normalized (log-transformed) and standardized across samples prior to subtype assignment. For each sample, Pearson correlation was computed between the sample expression profile and subtype-specific centroids, and the subtype with the highest correlation was assigned. A PAM50-based proliferation score was computed as the average standardized expression of 11 proliferation-associated genes within the PAM50 gene set (*BIRC5, CCNB1, CDC20, CEP55, MKI67, NDC80, PTTG1, RRM2, TYMS, UBE2C, MELK*). Gene expression values were first standardized (z-scored) across samples, and the proliferation score was calculated as the mean expression across the selected genes for each sample.

Values are expressed as average ± SE. Sequencing data are deposited in GEO databank, and the numbers are provided.

### Graphics

The visual abstract was created using Biorender.

## Supplementary information

Table EV1

Table EV2

Table EV3

Table EV4

Peer Review File

Dataset EV1

Dataset EV2

Dataset EV3

Source data Fig. 6

Dataset EV4

Expanded View Figures

## Data Availability

All sequencing data were uploaded to Gene Expression Omnibus (GEO) database and accession numbers are provided below (GSE329224, GSE329389, and GSE329391. The source data of this paper are collected in the following database record: biostudies:S-SCDT-10_1038-S44321-026-00530-5.
