# Supplementary material for: Intratumoral androstene-3,17-dione defines breast cancer subtype and prognosis
Source: EMBO Mol Med. 2026 Oct 1;18(11):4468–93. doi: 10.1038/s44321-026-00530-5 (PMC13748591; doi:10.1038/s44321-026-00530-5)
Supplement: Supplementary file 1 [file 44321_2026_530_MOESM11_ESM.pdf]

Expanded View Figures

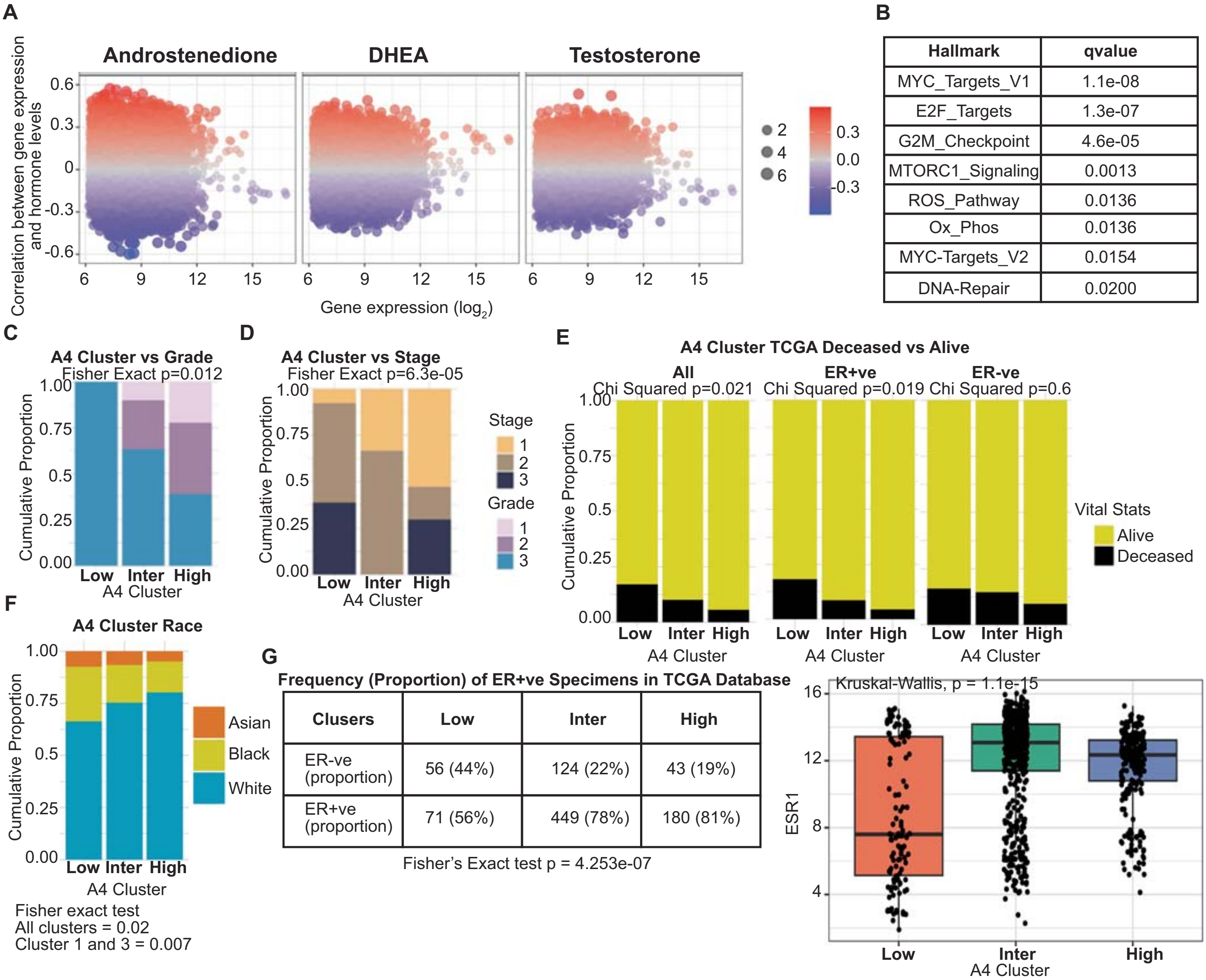

Hallmark	qvalue
MYC_Targets_V1	1.1e-08
E2F_Targets	1.3e-07
G2M_Checkpoint	4.6e-05
MTORC1_Signaling	0.0013
ROS_Pathway	0.0136
Ox_Phos	0.0136
MYC-Targets_V2	0.0154
DNA-Repair	0.0200

Clusers	Low	Inter	High
ER-ve (proportion)	56 (44%)	124 (22%)	43 (19%)
ER+ve (proportion)	71 (56%)	449 (78%)	180 (81%)

Figure EV1. A4-dependent signature correlates with better prognosis.

(**A**) Correlation between gene expression and various hormone levels. (**B**) *P* values of the top pathways that are negatively correlated with A4 levels. The 1388 genes significantly correlated with A4 were used for unsupervised subtype detection by K-means clustering ($n = 61$). Tumor grade (**C**) and stage (**D**) between three A4-dependent gene clusters. (**E**, **F**) Clinical and gene expression data extracted from the TCGA BC database ($n = 973$) were used to create clusters with 1231 of 1388 genes identified in Fig. 2. (**E**) Alive and deceased patients separated according to the three A4 clusters. (**F**) Proportion of patients' race in different clusters in the TCGA dataset. (**G**) Expression of ER+ve and ER-ve specimens in each cluster in the TCGA dataset ($n = 923$). Right: ESR1 gene expression in the TCGA dataset separated based on the three clusters. Box plots show the median (center line), interquartile range (box boundaries, 25th and 75th percentiles), and whiskers extending to the most extreme data points within 1.5 × the interquartile range. Individual data points are shown.

A

	ER-ve (N=22)	ER+ve (N=66)	Overall (N=88)
**PR**			
-	18 (81.8%)	9 (13.6%)	27 (30.7%)
+	4 (18.2%)	57 (86.4%)	61 (69.3%)
**HER2**			
-	21 (95.5%)	58 (87.9%)	79 (89.8%)
+	1 (4.5%)	6 (9.1%)	7 (8.0%)
Missing	0 (0%)	2 (3.0%)	2 (2.3%)
**AR**			
-	9 (40.9%)	10 (15.2%)	19 (21.6%)
+	9 (40.9%)	38 (57.6%)	47 (53.4%)
Missing	4 (18.2%)	18 (27.3%)	22 (25.0%)
**Ki67**			
Mean (SD)	49.8 (28.7)	40.2 (29.3)	42.9 (29.3)
Median [Min, Max]	51.0 [0, 99.0]	40.0 [0, 91.0]	45.0 [0, 99.0]
Missing	2 (9.1%)	15 (22.7%)	17 (19.3%)
**Race**			
AA	16 (72.7%)	27 (40.9%)	43 (48.9%)
WA	6 (27.3%)	38 (57.6%)	44 (50.0%)
Hisp.	0 (0%)	1 (1.5%)	1 (1.1%)

	ER-ve (N=22)	ER+ve (N=66)	Overall (N=88)
**Age**			
Mean (SD)	60.2 (10.6)	62.3 (13.6)	61.6 (12.7)
Median [Min, Max]	58.0 [39.0, 79.0]	65.5 [35.0, 83.0]	65.0 [35.0, 83.0]
Missing	3 (13.6%)	20 (30.3%)	23 (26.1%)
**Age (Binary)**			
Low	10 (45.5%)	19 (28.8%)	29 (33.0%)
High	9 (40.9%)	27 (40.9%)	36 (40.9%)
Missing	3 (13.6%)	20 (30.3%)	23 (26.1%)
**Stage**			
1	4 (18.2%)	17 (25.8%)	21 (23.9%)
2	8 (36.4%)	17 (25.8%)	25 (28.4%)
3	7 (31.8%)	9 (13.6%)	16 (18.2%)
Missing	3 (13.6%)	23 (34.8%)	26 (29.5%)
**Grade**			
1	1 (4.5%)	3 (4.5%)	4 (4.5%)
2	1 (4.5%)	23 (34.8%)	24 (27.3%)
3	16 (72.7%)	19 (28.8%)	35 (39.8%)
Missing	4 (18.2%)	21 (31.8%)	25 (28.4%)
**Recurrence**			
None	7 (31.8%)	31 (47.0%)	38 (43.2%)
Yes	8 (36.4%)	6 (9.1%)	14 (15.9%)
Missing	7 (31.8%)	29 (43.9%)	36 (40.9%)

8 samples have missing ER values

B

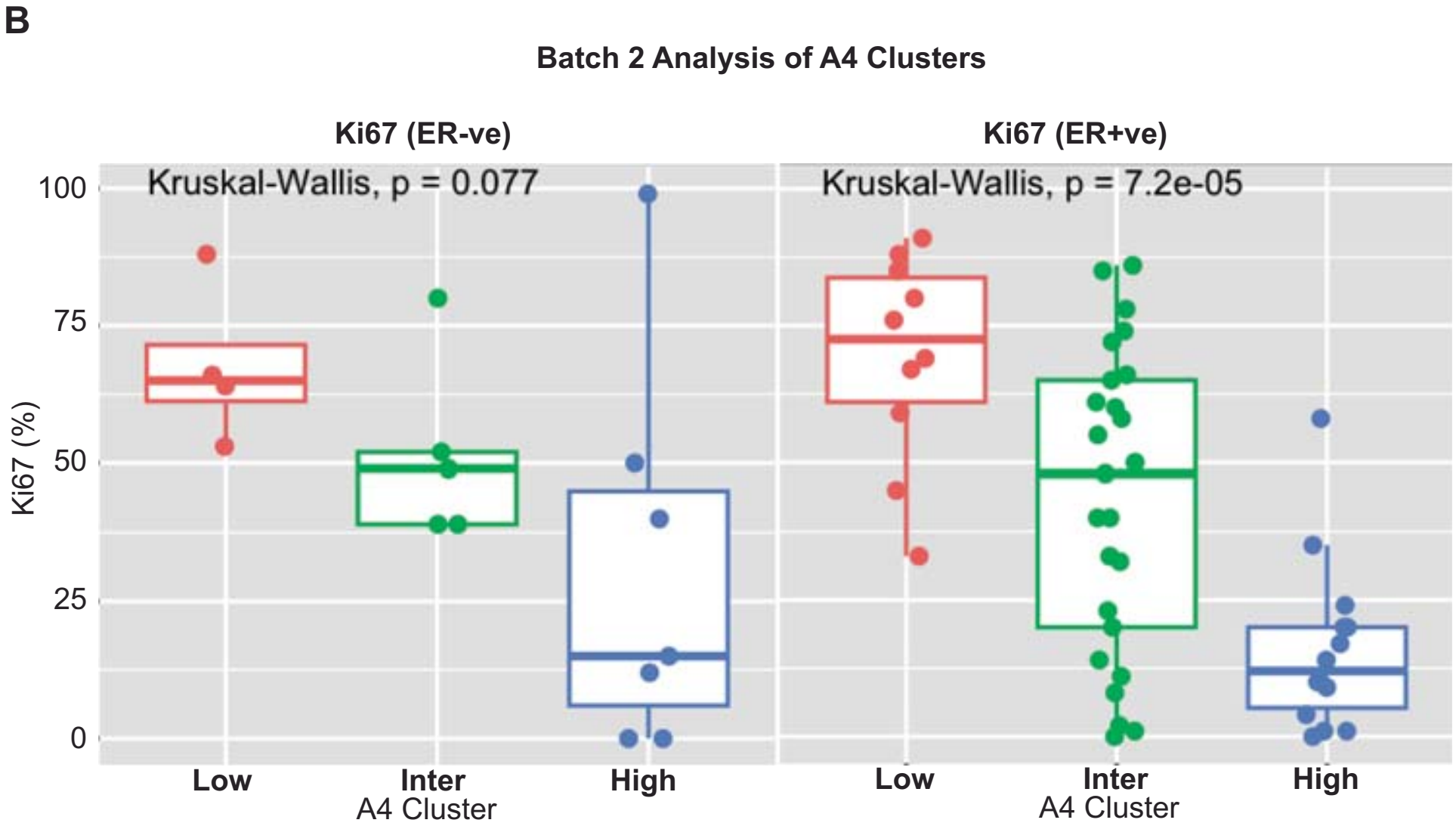

 © The Author(s)

◀ **Figure EV2.** (**A**) Clinical and histological variables of validation cohort ($n = 88$). (**B**) Ki67 levels in the validation cohort based on the three clusters obtained from A4-correlated genes ($n = 77$; Gene expression data were unavailable for 11 out of the 88 patients). Box plots show the median (center line), interquartile range (box boundaries, 25th and 75th percentiles), and whiskers extending to the most extreme data points within 1.5 × the interquartile range. Individual data points are shown.

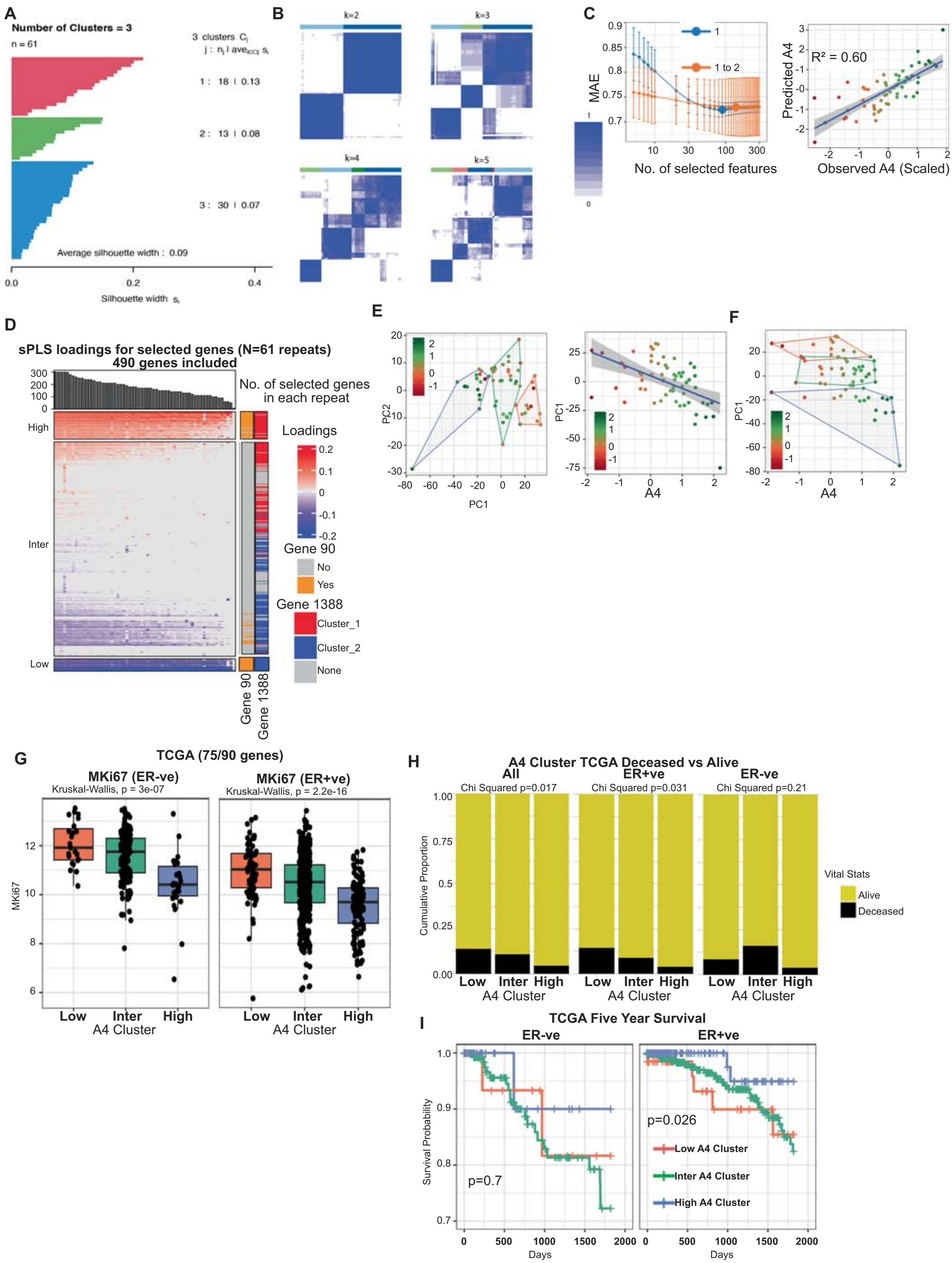
A
Number of Clusters = 3
n = 61
Average silhouette width : 0.09
Silhouette width s_i
B
k=2
k=3
k=4
k=5
C
MAE
No. of selected features
R² = 0.60
Predicted A4
Observed A4 (Scaled)
D
sPLS loadings for selected genes (N=61 repeats)
490 genes included
No. of selected genes
in each repeat
Loadings
High
Inter
Low
Gene 90
No
Yes
Gene 1388
Cluster_1
Cluster_2
None
E
PC1
PC2
A4
F
G
TCGA (75/90 genes)
MKi67 (ER-ve)
Kruskal-Wallis, p = 3e-07
MKi67 (ER+ve)
Kruskal-Wallis, p = 2.2e-16
A4 Cluster
H
A4 Cluster TCGA Deceased vs Alive
All
Chi Squared p=0.017
ER+ve
Chi Squared p=0.031
ER-ve
Chi Squared p=0.21
Cumulative Proportion
Vital Stats
Alive
Deceased
I
TCGA Five Year Survival
Survival Probability
p=0.7
p=0.026
Low A4 Cluster
Inter A4 Cluster
High A4 Cluster
Days

 © The Author(s)

Figure EV3. Computational analysis method to obtain the 90 gene signature.

(**A**, **B**) Validation of the approach used in the main manuscript ($n = 61$). See "Methods" for details. (**C**) Mean absolute error (MAE) obtained from fivefold cross-validation across varying numbers of selected genes for comparing models with one dimension (Component 1 only) and two dimensions (Components 1 and 2) ($n = 61$). Error bars represent standard deviations across cross-validation runs. (**D**) Further validation to assess the robustness of the selected gene set ($n = 61$). Leave-one-out cross-validation (LOOCV) approach to evaluate the selected 90 genes. (**E**, **F**) Principal component analysis (PCA) on the expression data of 1388 genes previously identified as highly associated with A4 ($n = 61$). Two-dimensional representation using the first two principal components (PCs). Solid polygons represent convex hulls enclosing samples within each A4 cluster. Regression lines were fitted using linear regression, and shaded regions represent 95% confidence intervals around the fitted regression lines. (**G**–**I**) TCGA BC validation of the 90 genes (75 out of the 90 genes were detected in the TCGA cohort; $n = 973$). (**G**) MKI67 gene expression levels in the TCGA dataset that were segregated into three A4 clusters. (**H**) Alive and deceased patients separated according to the three A4 clusters. (**I**) Kaplan–Meier (KM) plot of patient 5-year survival of the TCGA dataset. Survival differences between groups were evaluated using the log-rank test. KM plots were obtained for ER+ve and ER-ve. Box plots show the median (center line), interquartile range (box boundaries, 25th and 75th percentiles), and whiskers extending to the most extreme data points within 1.5 × the interquartile range. Individual data points are shown

© The Author(s)

A

Cluster	1	2	3	4	5	6	7
Cells	3454	2966	2437	2435	1199	737	200

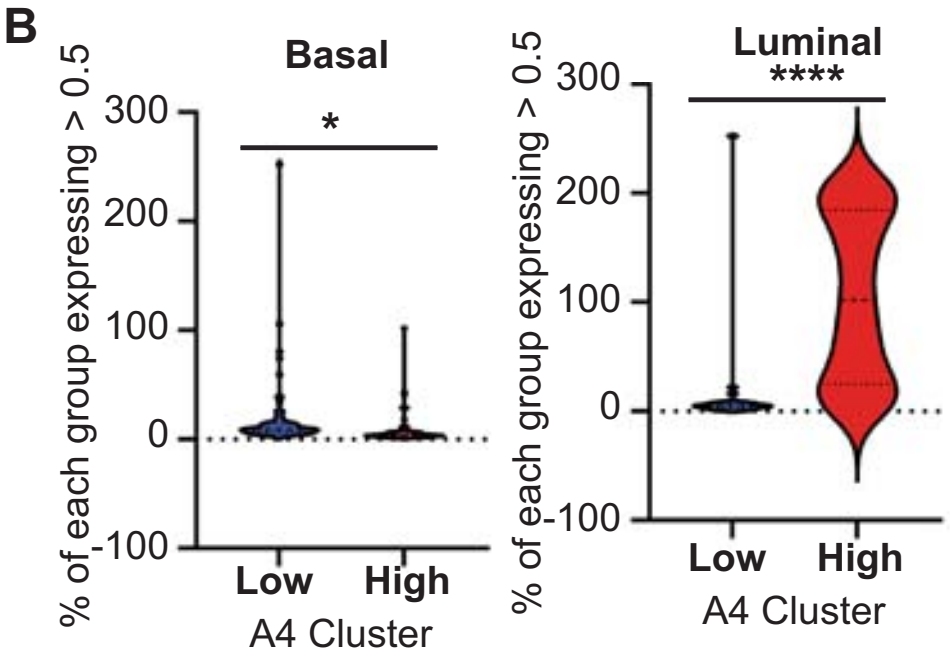

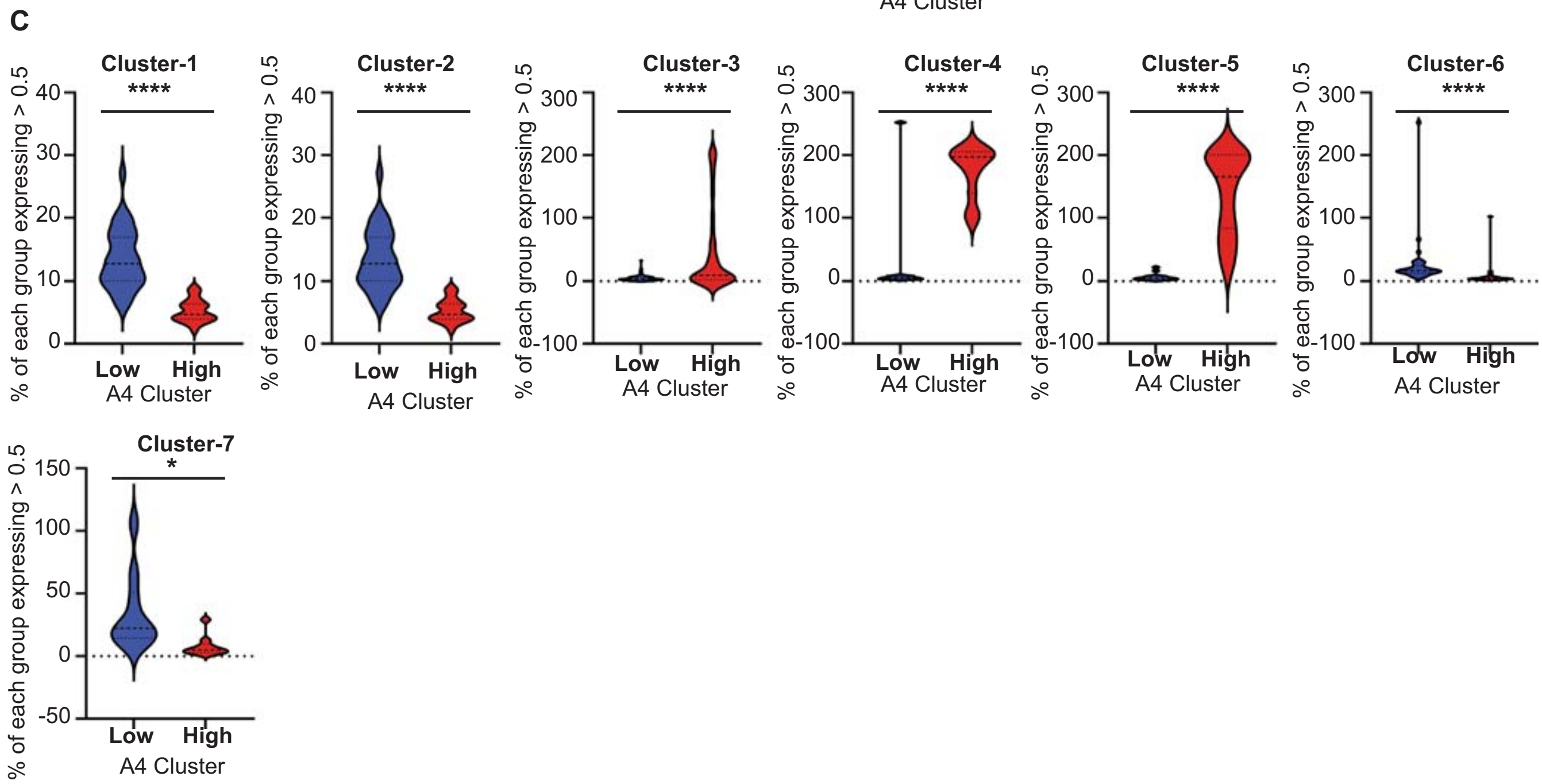

Figure EV4. Single-nucleotide RNA sequencing.

(**A**) Number of cells in each cluster. (**B**) Violin plot for basal and luminal cells signature. (**C**) Violin plots for all seven cluster signatures. *$P < 0.05$; **$P < 0.01$; ***$P < 0.001$; ****$P < 0.0001$. A4 low $n = 7357$ nuclei and A4 high $n = 6071$ nuclei. Data were analyzed by Student's t test ($n = 3$). Dashed horizontal lines within the violin plots indicate the median and interquartile range.

© The Author(s)
