# Supplementary material for: Intratumoral androstene-3,17-dione defines breast cancer subtype and prognosis
Source: EMBO Mol Med. 2026 Oct 1;18(11):4468–93. doi: 10.1038/s44321-026-00530-5 (PMC13748591; doi:10.1038/s44321-026-00530-5)
Supplement: Supplementary file 2 [file 44321_2026_530_MOESM5_ESM.pdf]

EMBO Molecular Medicine

Intratumoral androstene-3,17-dione Defines Breast Cancer Subtype and Prognosis

Sarah Asemota, Hyo Young Choi, Wendy Effah, Jeremiah Holt, Kirsten Young, Linnea Cripe, Keely McNamara, Dong Jun Byun, Hae Jin Seo, Suriyan Ponnusamy, Daniel Johnson, Jin-Young Lee, Yekta Khosroseshki, Brandy Grimes, Martin Fleming, Frances Pritchard, Ashley Hendrix, MIchael Berry, Richard Fine, Richard Gilmore, Farhan Khan, Liza Makowski, Balaji Santhanam, David Hayes, Hironobu Sasano, Man Ho Choi, and Ramesh Narayanan

Corresponding author(s): Ramesh Narayanan (RNARAYA4@UTHSC.EDU) , Hyo Young Choi (hyoyoung.choi@uthsc.edu)

Review Timeline:

Submission Date:	30th Dec 25
Editorial Decision:	10th Feb 26
Revision Received:	4th May 26
Editorial Decision:	15th Jun 26
Revision Received:	10th Jul 26
Editorial Decision:	2nd Aug 26
Revision Received:	2nd Aug 26
Editorial Decision:	3rd Sep 26
Revision Received:	7th Sep 26
Accepted:	10th Sep 26

Editor: Lise Roth

Transaction Report:

(Note: With the exception of the correction of typographical or spelling errors that could be a source of ambiguity, letters and reports are not edited. Depending on transfer agreements, referee reports obtained elsewhere may or may not be included in this compilation. Referee reports are anonymous unless the Referee chooses to sign their reports.)

10th Feb 2026

Dear Dr. Narayanan,

Thank you for submitting your work to EMBO Molecular Medicine, and please accept my apologies for the delay in responding, which was due to one referee requiring additional time to complete their report. We have now received reports from the three referees who agreed to evaluate your manuscript. As you will see below, while the reviewers acknowledge the potential interest of the study, they nevertheless raise substantial concerns about your work, which unfortunately preclude its publication in EMM in its current form.

If you feel you can satisfactorily address the points listed by the referees, you may wish to submit a revised version of your manuscript. Please attach a covering letter giving details of the way in which you have handled each of the points raised by the referees. A revised manuscript will once again be subject to review, and we cannot guarantee at this stage that the eventual outcome will be favorable.

As revising the manuscript according to the referees' recommendations appears to require a lot of additional work and experimentation, and given the potential interest of your findings, we are ready to extend the deadline to 6 months. Should you find that the requested revisions are not feasible within the constraints outlined here and choose, therefore, to submit your paper elsewhere, we would welcome a message to this effect.

If you would like to discuss further the points raised by the referees, I am available to do so via email or video. Let me know if you are interested in this option.

When submitting your revised manuscript, please carefully review the instructions that follow below. We perform an initial quality control of all revised manuscripts before re-review; failure to include requested items will delay the evaluation of your revision.

We require:

1) A .docx formatted version of the manuscript text (including legends for main figures, EV figures and tables). Please make sure that the changes are highlighted to be clearly visible.

2) Individual production quality figure files as .eps, .tif, .jpg (one file per figure). For guidance, download the 'Figure Guide PDF': https://media.springernature.com/original/springer-cms/rest/v1/content/27825798/data/v1.

3) A .docx formatted letter INCLUDING the reviewers' reports and your detailed point-by-point responses to their comments. As part of the EMBO Press transparent editorial process, the point-by-point response is part of the Review Process File (RPF), which will be published alongside your paper.

4) A complete author checklist, which you can download from our author guidelines. Please insert information in the checklist that is also reflected in the manuscript. The completed author checklist will also be part of the RPF.

5) Please note that all corresponding authors are required to supply an ORCID ID for their name upon submission of a revised manuscript.

6) It is mandatory to include a 'Data Availability' section after the Materials and Methods. Before submitting your revision, primary datasets produced in this study need to be deposited in an appropriate public database, and the accession numbers and database listed under 'Data Availability'. Please remember to provide a reviewer password if the datasets are not yet public.

In case you have no data that requires deposition in a public database, please state so in this section. Note that the Data Availability Section is restricted to new primary data that are part of this study.

7) For data quantification: please specify the name of the statistical test used to generate error bars and P values, the number (n) of independent experiments (specify technical or biological replicates) underlying each data point and the test used to calculate p-values in each figure legend. The figure legends should contain a basic description of n, P and the test applied. Graphs must include a description of the bars and the error bars (s.d., s.e.m.).

8) At EMBO Press we ask authors to provide source data for the main manuscript figures. You will receive a separate email with instructions for providing source data with your revised manuscript, including how to upload and organize the files.

9) Our journal encourages inclusion of *data citations in the reference list* to directly cite datasets that were re-used and obtained from public databases. Data citations in the article text are distinct from normal bibliographical citations and should

directly link to the database records from which the data can be accessed. In the main text, data citations are formatted as follows: "Data ref: Smith et al, 2001" or "Data ref: NCBI Sequence Read Archive PRJNA342805, 2017". In the Reference list, data citations must be labeled with "[DATASET]". A data reference must provide the database name, accession number/identifiers and a resolvable link to the landing page from which the data can be accessed at the end of the reference.

10) We replaced Supplementary Information with Expanded View (EV) Figures and Tables that are collapsible/expandable online. A maximum of 5 EV Figures can be typeset. EV Figures should be cited as 'Figure EV1, Figure EV2" etc... in the text and their respective legends should be included in the main text after the legends of regular figures.

- For the figures that you do NOT wish to display as Expanded View figures, they should be bundled together with their legends in a single PDF file called *Appendix*, which should start with a short Table of Content. Appendix figures should be referred to in the main text as: "Appendix Figure S1, Appendix Figure S2" etc.

- Additional Tables/Datasets should be labeled and referred to as Table EV1, Dataset EV1, etc. Legends have to be provided in a separate tab in case of .xls files. Alternatively, the legend can be supplied as a separate text file (README) and zipped together with the Table/Dataset file.

11) The paper explained: EMBO Molecular Medicine articles are accompanied by a summary of the articles to emphasize the major findings in the paper and their medical implications for the non-specialist reader. Please provide a draft summary of your article highlighting
- the medical issue you are addressing,
- the results obtained and
- their clinical impact.

This may be edited to ensure that readers understand the significance and context of the research. Please refer to any of our published articles for an example.

12) Author contributions: You will be asked to provide CRediT (Contributor Role Taxonomy) terms in the submission system. These replace a narrative author contribution section in the manuscript.

13) A "Disclosure and Competing Interests Statement" should be provided in the main text.

14) Every published paper includes a 'Synopsis' to further enhance discoverability. Synopses are displayed on the journal webpage and are freely accessible to all readers. They include a short stand first (maximum of 300 characters, including space) as well as 2-5 one-sentences bullet points that summarizes the paper. Please write the bullet points to summarize the key NEW findings. They should be designed to be complementary to the abstract - i.e. not repeat the same text. We encourage inclusion of key acronyms and quantitative information (maximum of 30 words / bullet point). Please use the passive voice. Please attach these in a separate file or send them by email, we will incorporate them accordingly.

Please also suggest a striking image or visual abstract to illustrate your article as a PNG file 550 px wide x 300-600 px high.

15) Include a Reagents and Tools Table as part of the Methods section, which can be downloaded from our author guidelines.

16) As part of the EMBO Publications transparent editorial process initiative (see our Editorial at http://embomolmed.embopress.org/content/2/9/329), EMBO Molecular Medicine will publish online a Review Process File (RPF) to accompany accepted manuscripts.
In the event of acceptance, this file will be published in conjunction with your paper and will include the anonymous referee reports, your point-by-point response and all pertinent correspondence relating to the manuscript. Let us know whether you agree with the publication of the RPF and as here, if you want to remove or not any figures from it prior to publication.
Please note that the Authors checklist will be published at the end of the RPF.

EMBO Molecular Medicine has a "scooping protection" policy, whereby similar findings that are published by others during review or revision are not a criterion for rejection. Should you decide to submit a revised version, I do ask that you get in touch after three months if you have not completed it, to update us on the status.

I look forward to receiving your revised manuscript.

Yours sincerely,

Lise Roth

Lise Roth, PhD

Senior Editor
EMBO Molecular Medicine

***** Reviewer's comments *****

Referee #1 (Remarks for Author):

This manuscript addresses an important and relatively underexplored aspect of hormone receptor-positive breast cancer biology by focusing on intratumoral androstenedione (A4) levels rather than circulating hormones, and by linking these measurements to transcriptional programs, tumor characteristics, and clinical outcome. While androgen receptor expression and androgen signaling have been studied previously in breast cancer, the systematic quantification of intratumoral A4 and the derivation of an A4-associated gene signature with proposed prognostic relevance represent a novel conceptual angle, particularly in ER-positive disease. The overall approach is ambitious and the datasets are substantial. However, I found the manuscript difficult to fully interpret in its current form. In particular, the absence of formal figure legends made it challenging to evaluate the data, as key information regarding panel structure, experimental conditions, normalization procedures, replicate numbers, and statistical analyses must be inferred from the figures themselves or pieced together from dispersed statements in the text. Given the complexity of the analyses presented, complete and standalone figure legends are essential to enable clear interpretation and proper assessment of the study's conclusions.

Please find my specific comments below:

1. The central claim that intratumoral androstenedione (A4) functions as a tumor suppressor in hormone receptor-positive breast cancer is supported almost entirely by correlative analyses linking A4 levels to proliferation, grade, stage, survival, and transcriptional programs. While these correlations are internally consistent and reproduced across cohorts, the manuscript frequently adopts causal language that is not warranted by the presented data. In particular, the inference that A4 "prevents the development of ER-negative tumors" or actively enforces a luminal state goes beyond what can be concluded from cross-sectional measurements. At minimum, the authors should more explicitly frame A4 as a marker of a differentiated, low-proliferative state, and carefully distinguish whether A4 is a driver of this state, a consequence of it, or both. Clarifying this distinction is essential for the conceptual integrity of the study.

2. The manuscript repeatedly treats A4-correlated transcriptional signatures as a proxy for functional androgen receptor (AR) signaling, yet this assumption is not rigorously tested. A4 is a weak AR agonist relative to DHT and testosterone, and is also a substrate for aromatization. The observation that classical androgen response signatures are not enriched raises an important but unresolved question: what molecular program is being captured by the A4 signature? Is it AR-mediated, indirectly AR-dependent, or reflective of broader metabolic or differentiation states? The preclinical AR knockdown experiments demonstrate that A4's antiproliferative effect in vitro requires AR, but this does not establish that the bulk tumor transcriptomic signature is AR-driven. This mechanistic gap should be explicitly acknowledged and, where possible, addressed experimentally (for example, by integrating AR ChIP-seq-derived programs or testing AR-dependence of the 90-gene signature). This part is very crucial as I do believe these findings are interesting, however more in vitro testing is needed.

3. The unsupervised clustering of patients into low, intermediate, and high A4 groups underpins much of the manuscript's narrative. However, clustering is performed on genes pre-selected for correlation with A4, which risks circularity when downstream associations with A4 and clinical variables are assessed. While the authors partially mitigate this by validating clusters in TCGA and an internal cohort, it remains unclear how stable these clusters are to perturbations in gene selection, normalization, or clustering parameters. A more explicit sensitivity analysis would strengthen confidence that the observed stratification reflects underlying biology rather than analytical choice. Without this, the distinction between biologically discrete states and a continuous A4-associated gradient remains ambiguous.

4. The development of the 90-gene A4 predictor is technically well executed and clearly demonstrates internal robustness. However, its clinical and biological interpretation would benefit from further clarification. The predictor is trained to infer intratumoral A4 levels, yet it is subsequently presented as a prognostic tool for tumor grade, proliferation, and survival. This raises the important question of whether the signature provides information that is distinct from, or additive to, established transcriptional programs that already capture prognosis in ER-positive breast cancer. In particular, it remains unclear how orthogonal the 90-gene signature is to canonical proliferation markers (e.g. MKI67), intrinsic subtype classifiers, or widely used prognostic gene panels in ER-positive disease. Given the extensive availability of validated ER-positive breast cancer

signatures, the manuscript would be substantially strengthened by a direct comparison of predictive performance. Relevant benchmarks could include PAM50 intrinsic subtype classification, Oncotype DX (21-gene recurrence score), EndoPredict, Prosigna, Breast Cancer Index, and IHC-based surrogates such as IHC4. A multivariate framework assessing whether the A4 signature retains independent prognostic value when adjusted for these established predictors, or improves risk stratification when added to them, would be particularly informative. Such analyses would help clarify whether the 90-gene signature captures a genuinely distinct biological axis related to intratumoral androgen biology, rather than largely recapitulating known luminal-basal or proliferation-driven transcriptional programs.

5. The single-nucleus RNA-seq analysis is conceptually appealing but remains underpowered and potentially overinterpreted. The analysis relies on three tumors (two A4-high, one A4-low), all of which are ER-positive, PR-positive, and AR-positive, limiting conclusions about broader subtype diversity. While the separation of luminal versus basal-like states is striking, it largely mirrors known differences between differentiated and aggressive tumors. Given the small sample size, claims regarding immune infiltration, stromal composition, and pathway repression should be framed as illustrative rather than definitive. The manuscript would benefit from a more cautious interpretation of these data and clearer acknowledgment of their exploratory nature.

6. A recurring conceptual tension in the manuscript concerns whether intratumoral A4 levels reflect tumor-intrinsic regulatory states or broader systemic and metabolic influences. Although the authors argue convincingly against reliance on circulating hormone measurements, intratumoral A4 may still be shaped by stromal content, local steroidogenic enzyme expression, or tissue composition differences between low-grade and high-grade tumors. The enrichment of fibroblasts and endothelial cells in A4-low tumors further complicates interpretation. The manuscript would be strengthened by explicitly discussing how tumor purity, stromal fraction, and intracrine enzyme expression might confound or contribute to measured A4 levels.

7. The Discussion positions A4 and the associated gene signature as having substantial diagnostic, prognostic, and therapeutic potential, including possible use as a companion diagnostic or predictor in benign tissue. These claims are ambitious relative to the evidence presented. The study does not assess longitudinal risk, treatment response, or predictive performance in clinical decision-making contexts. Moreover, proposing A4 as a therapeutic agent is conceptually problematic given its dual androgenic and estrogenic potential. A more restrained and evidence-aligned discussion of clinical implications would improve credibility and avoid overextension beyond the current data.

8. The functional assays presented in Figure 6 are intended to provide mechanistic support for the clinical observations, but in their current form they raise several concerns regarding experimental design, quantification, and interpretability. First, the AR transactivation assays (COS7 and T47D) appear under-sampled across the concentration range. The dose-response curves are fitted with a limited number of concentration points, particularly around the inflection region, making it difficult to reliably determine EC50 values or compare relative potency between A4 and DHT. Without sufficient data density in the dynamic range of the response, it is unclear whether the apparent shifts in activation reflect true differences in receptor activation or are artefacts of curve fitting. These assays should be repeated with additional concentrations included. Second, the proliferation assays raise questions regarding statistical analysis and biological relevance. In the endpoint SRB assay, the magnitude of the reported effect appears modest, yet statistical significance is claimed without a clear description of the statistical framework, sample size (biological vs technical replicates), or multiple-comparison correction across doses. Please, all of this should be disclosed and an additional cell line should be taken in these assays. Relatedly, the IncuCyte growth curves show only subtle separation between conditions, despite strong statistical claims. Given the visual overlap between curves, additional information is required on how statistical testing was performed for time-course data (e.g. area under the curve, mixed-effects models, or endpoint comparisons), and whether effect sizes are biologically meaningful. Improved visualization or complementary quantification would substantially strengthen this analysis. Additionally, another cell line should be used to confirm the effects. Third, the AR knockdown experiments require some changes. While AR siRNA abolishes the A4-dependent effect, it is unclear what the baseline effect of AR knockdown alone is on proliferation. Specifically, it is not evident whether the data are normalized to non-silencing siRNA within each treatment condition, or whether AR depletion itself alters baseline growth independently of A4. That is to solve this please show the non normalised data; as then we will be able to see the effect of AR knockdown as well.

Referee #2 (Comments on Novelty/Model System for Author):

There may be other explanations for the findings relating to how the androgen receptor may be the tumor suppressor only at high 4-AD.

Referee #2 (Remarks for Author):

This manuscript makes the case that high levels of 4-AD suppress breast cancer growth and progression, and low levels of 4-AD do the reverse. The evidence for this proposal involves correlative experiments showing an inverse relationship between intratumoral 4-AD levels measured by GC-MS with changes in the transcriptome that would promote tumor aggression. The transcriptome evidence is based on bulk-RNA, clinical data from TCGA and ultimately snRNA seq. The authors through validation of their first gene set enrichment promote a 90 gene classifier that may have prognostic value. There are a number of issues that need to be addressed.

1. The title of the article needs to be changed. 4-AD cannot be a tumor suppressor since this term is used for genes and not small molecules. The title should read "androstene-3,17-dione" suppresses hormone receptor positive breast cancer.

2. In hormone positive breast cancer the prevailing view has been that 4-AD would be converted to estrone by aromatase and this would be pro-proliferative. The authors suggest that this pathway may be by-passed by activation of the AR. To make this case the authors need to measure aromatase levels and perhaps E1 as well.

3. The authors show a large dynamic range of DHEA,4-AD and T in the tumor specimens and classify these levels from - 4 to +4. The authors should provide the steroid levels that correspond to these ordinal values. As these levels can vary by more than 100-fold do the authors have sufficient power to from meaningful gene clusters?

4. The measurement of hormone levels is crucial data for the manuscript. The authors describe what appears to be a nice GC-MS method with stable isotope internal standards. However, more details of this method should be given and how the authors compensated for any matrix effects from the tumor samples. Also, the authors should provide LLOQ, LLOD and precision and accuracy estimates, when analyzing the tissue samples.

5. The authors show almost equal potency between DHT and 4-AD in reporter gene assays in COS-7 and T47D cells. Have the authors investigated whether 4-AD recruits different coactivators to the AR? This could be supported by the statement on p12 which found only 2 genes significantly correlated with DHEA and T levels but more with 4-AD.

7. The authors should cite the work of the McDonnell laboratory (Safi et al. Nature Communications) which showed that high levels of androgens in the prostate cause AR dimerization and do not promote growth but instead cause cell differentiation; and that low levels of androgens working through monomeric AR in the cytosol promote growth . Could a similar mechanism explain the authors findings?

8. Is there any evidence that AR can regulate ER and PR expression in breast cancer?

9. The authors indicate that 1388 genes highly correlate with 4-AD; and then show that 4-AD levels correlate with gene expression. This seems like a circular argument and should be clarified.

10. The authors show collectively that high 4-AD specimens favor a better prognosis and therapy response while the low 4-AD specimens indicate aggressive growth and proliferation and then go on to indicate how these two groups differ in cell cycle phase. The differences in cell cycle phase are given as a single percentage. Should this not be a range given the range of 4-AD levels?

Minor:

1. Please provide the sources of all plasmids including the hAR.

2. Do not use the tern "dead" patients please change to "deceased"

Referee #3 (Comments on Novelty/Model System for Author):

the authors work mostly with patient samples the value of the validation with two cell line models is questionable.
A lot of progress in clinical breast cancer management has come from more differentiated diagnostic classification in particular different subtypes.Here correlations are made a wide tumor spectrum agnostic of different biologies that require different treatments. It is unlcear how a single signature should have prognostic and predictive power across such a broad spectrum of disease.

Referee #3 (Remarks for Author):

"Intratumoral Androstenedione is a Tumor Suppressor in Hormone Receptor-Positive Breast Cancer"

In this manuscript, Asemota et al. quantify androgen levels directly in breast cancer tissue and report that higher intratumoral androstenedione (A4) levels are associated with lower Ki67 and improved clinical outcome in ER-positive breast cancer. The study addresses an important and understudied aspect of tumor intracrinology and moves beyond the limitations of circulating hormone measurements. The availability of two patient cohorts and the integration of bulk RNA-seq and single-nucleus RNA-seq are notable strengths.
However, the current version remains largely correlative and would benefit from clearer framing of the underlying biological hypothesis, more methodological detail regarding tissue hormone measurements, and deeper mechanistic interrogation of androgen-estrogen signaling interactions. Several conclusions appear overstated relative to the data presented, and additional

analyses would substantially strengthen the manuscript.

Major points
1. Framing of prior literature and abstract statement
The opening statement of the abstract - "The role of androgens and androgen receptor in breast cancer in the clinic has been defined by correlating circulating androgen levels to clinical variables" - is misleading.
There is extensive preclinical literature as well as clinical trial data addressing AR signaling in breast cancer, and it is well established that AR biology differs fundamentally between ER-positive and ER-negative disease. This statement should be revised to accurately reflect the field and to clearly position the present study in relation to existing mechanistic and clinical work.
2. Biological hypothesis and mechanism
It remains unclear whether the authors hypothesize that androstenedione exerts tumor-suppressive effects via:
o AR activation and downstream anti-proliferative gene programs, or
o an alternative, AR-independent intracrine or paracrine mechanism.
If AR-mediated effects are proposed, this is neither novel nor directly demonstrated in the current study. If a different mechanism is envisioned, it should be clearly articulated in the Introduction and supported by appropriate analyses. At present, the mechanistic model underlying the key conclusions is insufficiently defined.
3. Hormone measurements and tissue intracrinology
Given that tumor intracrinology remains poorly characterized, more detail on the technical and biological aspects of tissue hormone measurements is essential. In particular:
o Which tissue-related or pre-analytical factors affect intratumoral hormone levels?
o Were any samples excluded due to hormone measurement quality, analogous to the RNA-seq exclusions due to RNA integrity?
o How robust are A4 measurements across tissue processing conditions?
Greater transparency here is critical for interpreting the correlations reported.
4. Context of estrogen signaling
The manuscript addresses an important question and provides intriguing correlative data on intratumoral androstenedione in ER-positive breast cancer. However, clearer mechanistic framing, improved methodological transparency, and additional analyses are required before the conclusions can be fully supported. In light of the extensive ER/AR interactions it is of interest to consider the findings in the context of ER signalling activity and estrogen levels.
5.Patient samples are selected "randomly" reflecting the full spectrum of patients, while this has some attraction, it is well established that the biology of hormone receptor signaling is very different between different tumor subtypes. As such hypotheses should be testable within specific subtypes. While the authors distinguish ER+ and ER- tumors, molecular subtypes should be taken into consideration.

Minor: to assess cell proliferation by RNAseq, the authors use KI67. Better to use the combined 11 or 14 proliferation genes from the PAM50 signature.

Addressing the points above would substantially enhance the impact and rigor of the study.

Reviewer-1: We thank the reviewer for the favorable opinion that this work is novel and is a potentially important contribution to the field. We also thank the reviewer for a critical review and providing several suggestions that significantly improved the quality and readability of the manuscript. We hope we have responded appropriately to the reviewer's satisfaction.

1. *I found the manuscript difficult to fully interpret in its current form. In particular, the absence of formal figure legends made it challenging to evaluate the data, as key information regarding panel structure, experimental conditions, normalization procedures, replicate numbers, and statistical analyses must be inferred from the figures themselves or pieced together from dispersed statements in the text. Given the complexity of the analyses presented, complete and standalone figure legends are essential to enable clear interpretation and proper assessment of the study's conclusions*.

   Response: We sincerely apologize for the oversight. The figure legends file did not get integrated into the manuscript body file when we submitted the original version. We thank the reviewer for bringing this to our attention. Now the standalone figure legends are incorporated into the body of the manuscript. The legends now contain details of experiment design and statistical analyses.

2. *The central claim that intratumoral androstenedione (A4) functions as a tumor suppressor in hormone receptor-positive breast cancer is supported almost entirely by correlative analyses linking A4 levels to proliferation, grade, stage, survival, and transcriptional programs. While these correlations are internally consistent and reproduced across cohorts, the manuscript frequently adopts causal language that is not warranted by the presented data. In particular, the inference that A4 "prevents the development of ER-negative tumors" or actively enforces a luminal state goes beyond what can be concluded from cross-sectional measurements. At minimum, the authors should more explicitly frame A4 as a marker of a differentiated, low-proliferative state, and carefully distinguish whether A4 is a driver of this state, a consequence of it, or both. Clarifying this distinction is essential for the conceptual integrity of the study*.

   Response: We thank the reviewer for this very nice comment. We have now revised the manuscript to eliminate conjunctures that are beyond the direct interpretation of the data. We have thoroughly reviewed the manuscript for such statements and revised it.

3. *The manuscript repeatedly treats A4-correlated transcriptional signatures as a proxy for functional androgen receptor (AR) signaling, yet this assumption is not rigorously tested. A4 is a weak AR agonist relative to DHT and testosterone, and is also a substrate for aromatization. The observation that classical androgen response signatures are not enriched raises an important but unresolved question: what molecular program is being captured by the A4 signature? Is it AR-mediated, indirectly AR-dependent, or reflective of*

broader metabolic or differentiation states? The preclinical AR knockdown experiments demonstrate that A4's antiproliferative effect in vitro requires AR, but this does not establish that the bulk tumor transcriptomic signature is AR-driven. This mechanistic gap should be explicitly acknowledged and, where possible, addressed experimentally (for example, by integrating AR ChIP-seq-derived programs or testing AR-dependence of the 90-gene signature). This part is very crucial as I do believe these findings are interesting, however more in vitro testing is needed.

Response: We thank the reviewer for this comment. We took multiple approaches to address this comment. A) performed a pathway analysis of the 90 gene signature; b) performed AR ChIP-seq in A4 high and low specimens; and c) performed gene expression pathway analysis of in vitro RNA sequencing data. We have now incorporated these analyses in the manuscript.

This table and text referred to data that was removed from the final version of the manuscript.

Additionally, "we examined overlap between the larger set of A4-associated genes and AR-target genes and established androgen-related gene sets. Among the 1,388 A4-associated genes, 98 overlapped with androgen-related gene sets curated in MSigDB, including 11 genes present in the HALLMARK_ANDROGEN_RESPONSE gene set (CDK6, HMGCS1, LIFR, MAP7, PIAS1, PLPP1, PTPN21, SAT1, SLC38A2, SMS, TPD52). These data suggest that there is an enrichment of androgen-dependent signature". The above information within quotes has been incorporated in the manuscript.

The ChIP-seq studies demonstrated that AR is recruited to its downstream target gene family myocyte enhancer factor (MEF2). Although androgen response elements (AREs) are not one of the motifs AR was recruited to, the AR is recruited to its downstream target genes' regulatory regions. The section on ChIP seq has been incorporated in the manuscript and pasted below for the reviewer's convenience.

This figure and text referred to data that was removed from the final version of the manuscript.

In vitro RNA seq GSEA analysis showed that androgen response pathway is one of the pathways enriched in A4-treated cells, while estrogen-responsive pathways are enriched in vehicle-treated cells, which is in concordance with our previous publications. The section is provided below for the reviewer's convenience.

> "Gene set enrichment analysis of the genes showed that androgen response is one of the hallmark pathways enriched in the A4-treated cells. In addition, estrogen-responsive pathways are enriched in vehicle-treated cells, concordant with our earlier publications that showed treatment of ER-positive BC tumors with androgens inhibits estrogen pathway(Asemota *et al.*, 2024; Ponnusamy *et al.*, 2019a)."

4. *The unsupervised clustering of patients into low, intermediate, and high A4 groups underpins much of the manuscript's narrative. However, clustering is performed on genes pre-selected for correlation with A4, which risks circularity when downstream associations with A4 and clinical variables are assessed. While the authors partially mitigate this by validating clusters in TCGA and an internal cohort, it remains unclear how stable these clusters are to perturbations in gene selection, normalization, or clustering parameters. A more explicit sensitivity analysis would strengthen confidence that the observed stratification reflects underlying biology rather than analytical choice. Without this, the distinction between biologically discrete states and a continuous A4-associated gradient remains ambiguous*.

   Response: a) We thank the reviewer for raising this important point regarding potential circularity. We would like to clarify that the goal of this analysis was not to test whether clusters differ in A4 levels. As the reviewer pointed out, because genes were selected based on correlation with a continuous A4 measurement, separation of tumors along the A4 axis is expected. Therefore, the key question is not whether clusters differ in A4, but whether tumors segregate into reproducible transcriptional states within the A4-associated gene space and these states are associated with independent clinical and pathological variables. Importantly, hormone measurements were not used in the clustering algorithm but only used post hoc for biological interpretation of the resulting transcriptional groups.
   We have clarified this in Results to avoid impression that clustering was performed to rediscover A4 differences: The section is provided below for the reviewer's convenience.

   > "A4-correlated genes form different transcriptional states across tumors. Unsupervised k-means clustering algorithm was applied to the 1388 genes whose expression significantly correlated with A4 (**Fig. 2C**). This clustering resulted in three tumor groups and two gene clusters, with gene expression patterns changing in a gradual manner across the three tumor groups, suggesting a continuous transcriptional gradient. Because genes were selected based on correlation with A4 levels, separation of tumors along the A4 axis is expected. The three transcriptional groups corresponded to tumors with low, intermediate, and high A4 levels, and were therefore labeled low-, intermediate-, and high-A4 clusters. DHEA and testosterone levels showed similar increasing trends across clusters (although testosterone was not statistically significant), suggesting that A4-dependent clusters and genes in the clusters promote broad androgenic clustering (**Figs. 2C-D**)."

   b) We thank the reviewer for this insightful comment regarding stability of the identified clusters and the ambiguous distinction between biologically discrete states and a continuous A4-associated gradient. We agree that demonstrating stability to ensure that the observed clustering reflects underlying biology rather than analytical choices. To address

this, we performed multiple complementary analyses to evaluate cluster robustness and reproducibility.

1. Robustness to clustering parameters (number of clusters, initial seed of K-means clustering, and sampling variation)
First, we evaluated the stability of the clustering solution and the optimal number of clusters using both silhouette analysis and consensus clustering with 1,000 subsampling iterations. The silhouette plot demonstrated good separation between clusters with no negative silhouette widths, indicating that samples were appropriately assigned into three clusters. The consensus clustering analysis showed stable sample co-clustering patterns and supported a three-cluster solution as a robust structure in the data. These analyses indicate that the identified clusters are not driven by random sampling variation or clustering initialization.

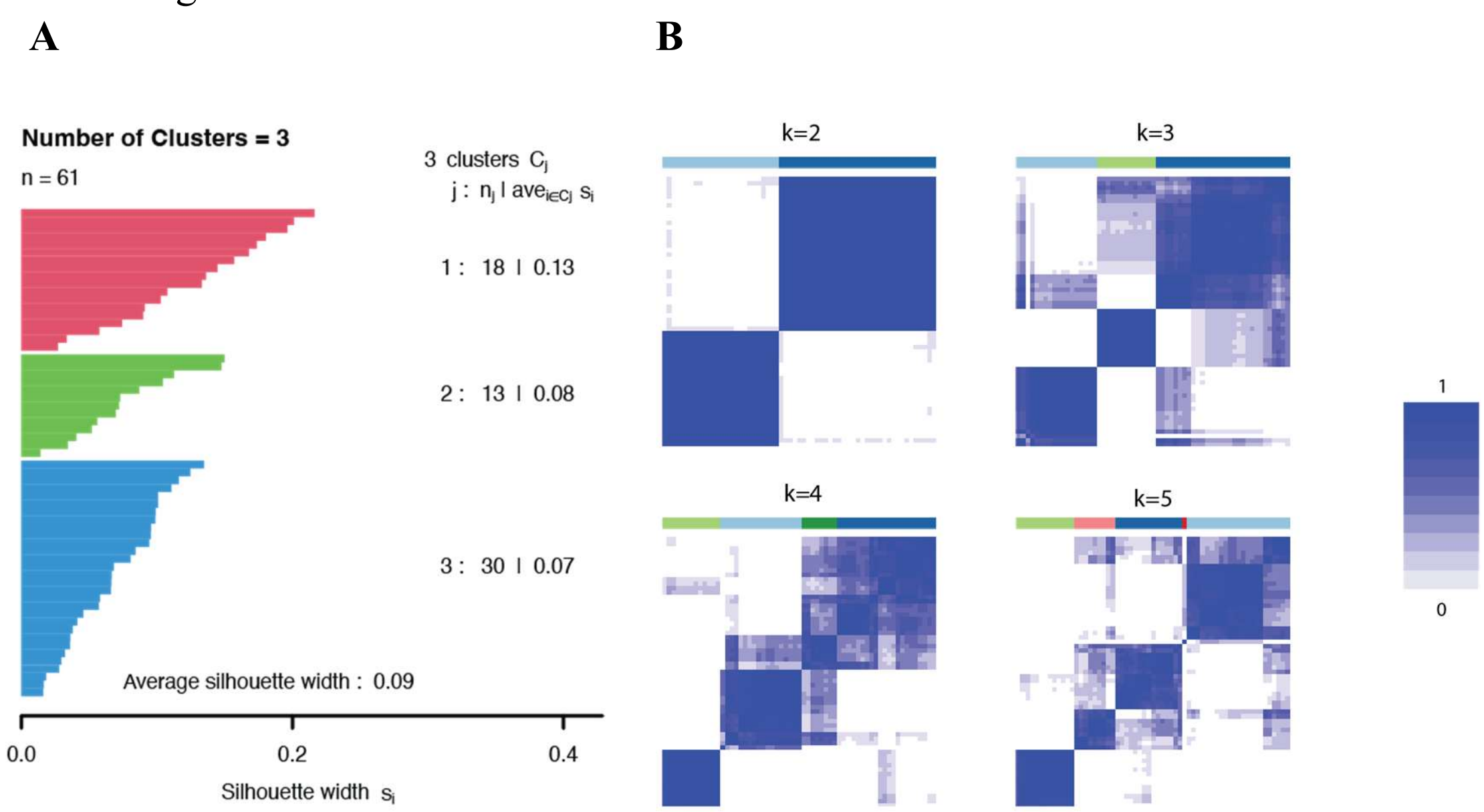

We have the following text In Methods:

"The 1,388 genes that significantly correlated with A4 were used for unsupervised subtype detection by K-means clustering. We employed a silhouette plot as well as a consensus matrix to determine the optimal number of clusters using R packages cluster and ConsensusClusterPlus(Wilkerson & Hayes, 2010). A silhouette plot shows the relative distance of how close a sample in one group is to a nearby group,

which is indicated by the silhouette width ranging from –1 to 1 (**Fig. EV4A**). A value close to 1 represents good separation from the neighboring clusters whereas a negative value implies that the corresponding sample has been assigned to the wrong cluster. The resulting silhouette plot shows a good separation between clusters with no negative values for each sample (**Fig. EV4A**). Additionally, the consensus matrix, a symmetric display of consensus values between sample pairs, indicates consensus levels with dark blue representing high consensus where samples consistently cluster together. The resulting consensus matrix, based on x1000 subsampling iterations, suggests that three clusters (k=3) are reasonable number of underlying clusters (**Fig. EV4B**). While a total of two clusters (k=2) also demonstrate good separation, observing the gradual changes in gene expression profiles indicates that k=3 would be more appropriate. This enables the comparison between two extreme clusters, Clusters 1 (low A4 group) vs. 3 (high A4 group), which might have greater clinical relevance."

2. Robustness to gene selection

Second, we evaluated whether the same clustering structure could be recovered using alternative gene selection. Principal component analysis (PCA) of the 1,388 A4-associated genes showed that the first PC (PC1) strongly correlated with A4 levels and alone separated the three patient groups, indicating that the clustering structure is largely driven by a single dominant biological axis rather than being dependent on the specific clustering algorithm.

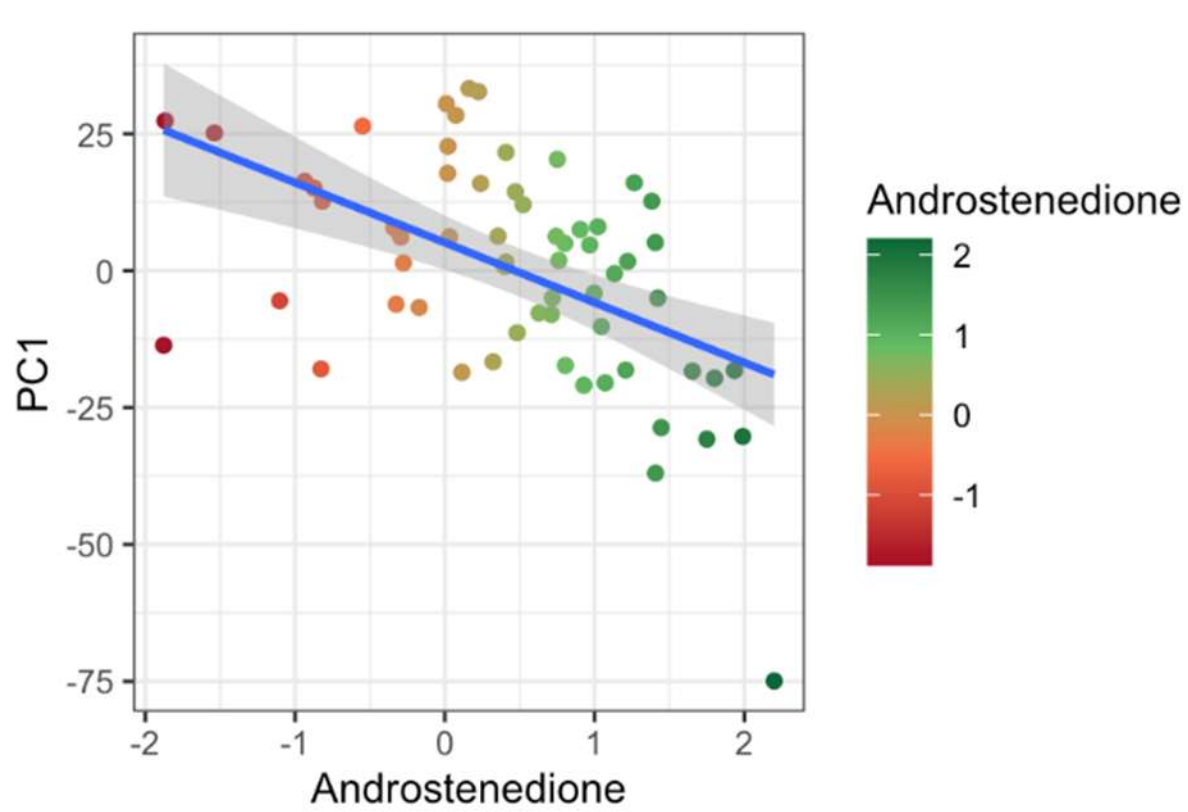

Consistent with this, a supervised sparse PLS model independently identified a reduced 90-gene set predictive of A4 levels. The robustness of this gene set was evaluated using leave-one-out cross-validation (LOOCV) with re-tuning of feature selection in each iteration. The consistent selection of genes across cross-validation iterations and the strong correlation between predicted and observed A4 levels indicate that A4-associated gene set is stable and not dependent on a specific gene subset. Importantly, clustering and classification using this reduced gene set reproduced the same three patient groups with high concordance and no switching between the extreme groups (low vs high) (**Figure 4A**). This demonstrates that the observed clustering is robust to gene selection and dimensionality reduction.

3. Robustness to normalization techniques

We also note that the centroid-based prediction method used in this study is relatively robust to differences in normalization. The subtype prediction in this study was performed using a nearest-centroid classifier, similar to the PAM50 classifier, in which each sample

is assigned to the subtype whose centroid expression profile shows the highest correlation with the sample's expression profile. Because correlation measures relative expression patterns across genes rather than absolute expression levels, this approach is less sensitive to differences in scaling and normalization across datasets. For this reason, nearest-centroid classifiers have been widely used for cross-platform molecular subtype prediction. The successful application of our centroid predictor to the TCGA RNA-seq dataset, which was processed independently, further supports that subtype assignment is robust to normalization differences and reflects underlying biological signal rather than technical variation.

4. The ambiguous distinction between biologically discrete states and a continuous A4-associated gradient

We agree that A4 activity likely exists on a continuous spectrum rather than forming strictly discrete tumor subtypes. The discrete A4-low, -intermediate, and -high groups were introduced primarily as an interpretability framework to facilitate clinical comparisons, particularly between two extreme states (A4-low vs. A4-high), where biological and clinical differences were most apparent.

In our data, raw A4 levels did not show clear associations with clinical variables. However, when tumors were grouped into low, intermediate, and high A4-associated transcriptional states, we were able to more clearly identify associations with proliferation, tumor grade, and clinical outcomes particularly due to two extreme states. This is because the intermediate group appeared to represent tumors with mixed or transitional expression patterns, which reduced statistical power when treated as a single continuous variable.

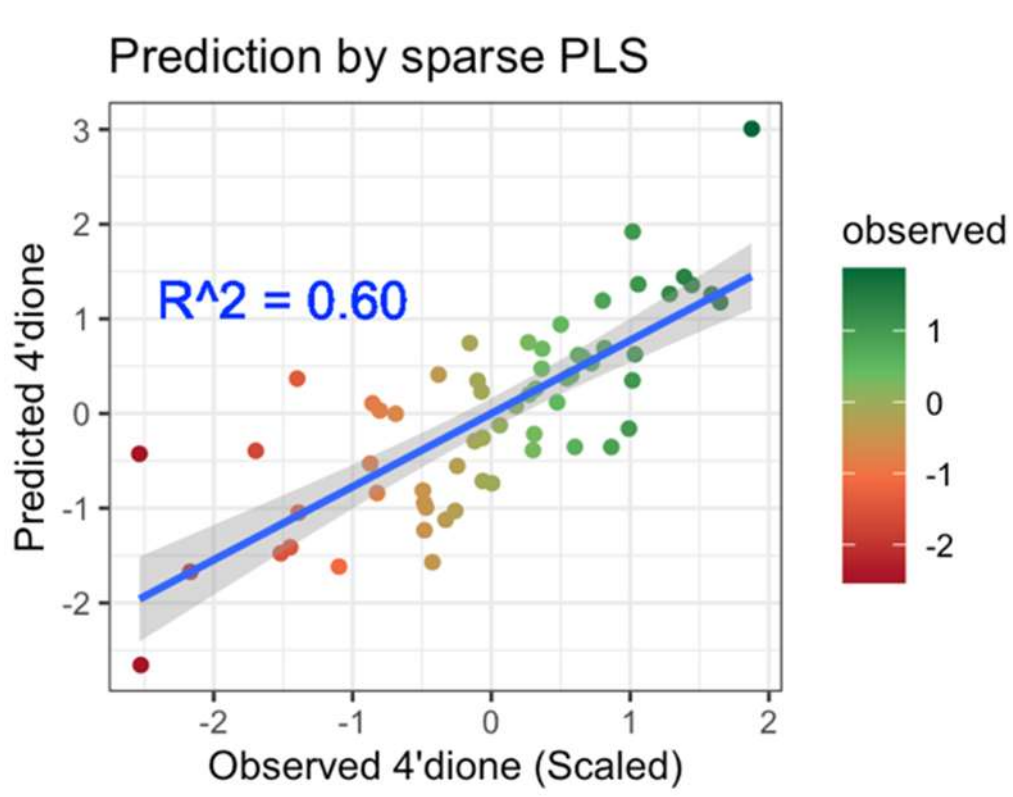

Having observed the strong associations using the discrete A4 states, we further investigated whether we can predict A4 levels directly at a continuous scale. We developed a reduced gene predictor using sparse partial least squares (sPLS), which identified a 90-gene set predictive of A4 levels (Full text can be found in "A4-dependent predictor signature" in Results). In this analysis, we were able to observe strong correlation with the predicted A4 and the measured A4 levels, which indicates that gene expression profiles capture a continuous signal associated with intratumoral androstenedione activity. This finding is particularly interesting because direct measurement of A4 levels in tumor tissues requires specialized assays that are not routinely available, whereas gene expression data are more widely accessible. Importantly, this analysis also supports the interpretation that the underlying biological structure is continuous rather than strictly discrete. The three groups identified by clustering should be interpreted as representing low, intermediate, and high states along a continuous A4-associated transcriptional axis, rather than strictly discrete molecular subtypes.

We have revised the manuscript to clarify that these groups represent discretization of a continuous biological gradient for interpretability rather than strictly discrete tumor subtypes:

> "These results suggest that the primary source of variation among tumors is a continuous A4-associated transcriptional axis, and that k-means clustering effectively discretizes this gradient into low, intermediate, and high states, which show high clinical relevance."

5. *The development of the 90-gene A4 predictor is technically well executed and clearly demonstrates internal robustness. However, its clinical and biological interpretation would benefit from further clarification. The predictor is trained to infer intratumoral A4 levels, yet it is subsequently presented as a prognostic tool for tumor grade, proliferation, and survival. This raises the important question of whether the signature provides information that is distinct from, or additive to, established transcriptional programs that already capture prognosis in ER-positive breast cancer. In particular, it remains unclear how orthogonal the 90-gene signature is to canonical proliferation markers (e.g. MKI67), intrinsic subtype classifiers, or widely used prognostic gene panels in ER-positive disease. Given the extensive availability of validated ER-positive breast cancer signatures, the manuscript would be substantially strengthened by a direct comparison of predictive performance. Relevant benchmarks could include PAM50 intrinsic subtype classification, Oncotype DX (21-gene recurrence score), EndoPredict, Prosigna, Breast Cancer Index, and IHC-based surrogates such as IHC4. A multivariate framework assessing whether the A4 signature retains independent prognostic value when adjusted for these established predictors, or improves risk stratification when added to them, would be particularly informative. Such analyses would help clarify whether the 90-gene signature captures a genuinely distinct biological axis related to intratumoral androgen biology, rather than largely recapitulating known luminal-basal or proliferation-driven transcriptional programs*.

   Response: We thank the reviewer for this insightful comment. We would like to clarify that the primary goal of developing the 90-gene signature was not to create a new prognostic classifier, but rather to identify gene expression patterns associated with intratumoral androstenedione (A4) levels and to determine whether this androgen-associated expression patterns are related to tumor characteristics and clinical outcomes. We agree that it is important to understand how this signature relates to established predictors such as proliferation markers and intrinsic subtype. To address this, we performed additional analyses in the TCGA BRCA cohort to derive PAM50 subtypes (luminal A, luminal B, basal-like, HER2-enriched, and normal-like) and PAM50 proliferation scores. We computed a PAM50-based proliferation score using the proliferation-associated genes within the PAM50 gene set. Consistent with our observations using MKI67, A4-associated clusters were associated with proliferation (**Fig. 4H**). Subtype distribution analysis showed

that the A4-low cluster was enriched for Basal-like and Luminal B tumors, whereas the A4-high cluster was enriched for Luminal A, with the A4-intermediate cluster showing a mixed composition (**Fig. 4I**). These results indicate an association between A4 clusters and PAM50 intrinsic subtypes. However, each A4 cluster contained a mixture of subtypes, indicating that the A4-associated classification does not simply recapitulate intrinsic subtype.

Furthermore, survival analyses stratified by ER status and PAM50 subtype showed that even within the same PAM50 subtype, A4 clusters further stratified patient outcomes. Most notably within Luminal A, A4-low tumors showed significantly worse survival compared to A4-high tumors (P.val < 0.0001). This pattern was not observed in ER-negative tumors. These results suggest that the 90-gene signature reflects androgen-related tumor biology that is related to, but not identical to, known proliferation and intrinsic subtype-associated expression patterns, and provides additional prognostic dimension beyond these established markers.

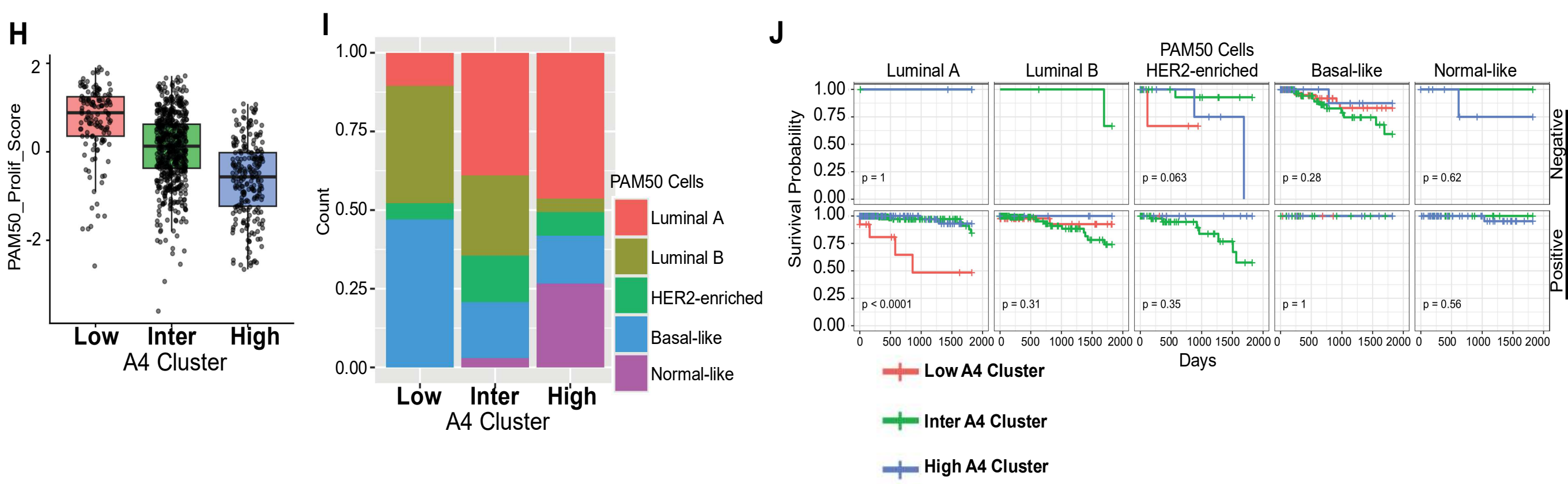

Figs. 4H-J.

We have clarified this point in the revised manuscript:

> "To assess the relationship between A4-associated gene expression and established BC classifiers, we evaluated PAM50 intrinsic subtypes and a PAM50-based proliferation score in the TCGA BRCA cohort. A4 clusters were significantly associated with proliferation inhibition, consistent with analyses using MKI67 (**Fig. 4H**), yet each cluster contained a mixture of subtypes (**Fig. 4I**). Notably, survival analyses stratified by ER status and PAM50 subtype showed that A4 clusters further stratified outcomes within the same subtype. It was most prominent within Luminal A where A4-low tumors exhibited worse survival than A4-high tumors in ER-positive tumors (**Fig. 4J**). These findings indicate that androgen-associated gene expression could capture additional prognostic dimension beyond conventional subtype classification."

6. *The single-nucleus RNA-seq analysis is conceptually appealing but remains underpowered and potentially overinterpreted. The analysis relies on three tumors (two A4-high, one A4-low), all of which are ER-positive, PR-positive, and AR-positive, limiting conclusions about broader subtype diversity. While the separation of luminal versus basal-like states is striking, it largely mirrors known differences between differentiated and aggressive tumors. Given the small sample size, claims regarding immune infiltration, stromal composition, and pathway repression should be framed as illustrative rather than definitive. The manuscript would benefit from a more cautious interpretation of these data and clearer acknowledgment of their exploratory nature*.

   Response: We thank the reviewer for this comment. We have revised this section by removing the overstatements and also including a more cautious interpretation. For the convenience of the reviewer, the entire scRNA seq section is provided below.

> "Single nuclear RNA sequencing (snRNA-seq) confirms the tumor inhibitory role of A4. The bulk RNA seq clearly demonstrated the beneficial effects of higher intratumoral A4, which corresponded to more luminal subtype BC, lower proliferation index, and better survival outcome. To confirm those findings at single cell level and to determine the cellular characteristics, snRNA seq was performed. Three patient specimens, two A4 high and one A4 low, were selected for this analysis. All three specimens were ER+ve, PR+ve, and AR+ve. The A4 low and one A4 high specimens were HER2-ve, PR+ve, and AR+ve. All three specimens had comparable intratumoral estradiol (1.56-2.57 ng/g tissue). The A4 low specimen contained 1.6 ng/g A4 and 6.9 ng/g DHEA, while the two A4 high specimens contained 9.2 ng/g and 13.4 ng/g A4 and 70.29 and 19.03 ng/g DHEA, clearly segregating the specimens into the two categories based on intratumoral androgens. Considering that all properties other than intratumoral A4 and DHEA are comparable, we expect the changes in cellular properties and signatures to be driven by intratumoral androgens.
>
> A4 low specimen had 7357 nuclei, while A4 high specimens contained 6071 nuclei sequenced (**Fig. EV5A**). Umap clearly showed a distinct separation of A4 high and low cells with no overlap (**Fig. 5A**). Low A4 specimen cells formed four clusters (cluster 1,2,6, and 7) while high A4 specimen cells formed three clusters (cluster 4,5, and 6) (**Fig. 5B**). Number of cells in each cluster is shown in **Fig. EV5A**. Evaluating the signatures for each cell type provided distinct type of cells in low A4 and high A4 specimens (**Fig. 5C**). Low A4 cells were enriched for endothelial cells, basal cells, macrophages (small proportion), and fibroblasts, while high A4 cells were enriched for luminal cells and t-cells (**Fig. 5C**). The luminal cells are typically ER+ve PR+ve, and contribute to better prognosis and response to treatment, while basal cells are ER-ve and are aggressive cell types that lack any therapeutic targets(Malzahn *et al*, 1998; Oakes *et al*, 2014; Perou *et al*, 2000). This cell type identification (**Figs. EV5B and 5C**) agrees with the ER+ve and TNBC subtypes identified in the bulk RNA seq (**Figs. 2C-G; EV5B**). The A4 low

specimens are also enriched for fibroblasts and endothelial cells, which are components of the stromal compartment(Mao *et al*, 2013). The fibroblasts, such as cancer-associated fibroblasts, support tumorigenic microenvironment(Mao *et al.*, 2013). The endothelial cells that are responsible for the construction of blood vessels promote angiogenesis, invasion, and migration(Longatto Filho *et al*, 2010). The fibroblasts and endothelial cells form a significant part of the tumor microenvironment. Endothelial cells also play an important role in breast epithelial growth and branching and stimulating growth of cancer-derived cells(Ingthorsson *et al*, 2010; Sigurdsson *et al*, 2006). Similarly, macrophages play an important role in angiogenesis and blood vessel formation(Leek *et al*, 1996; Lewis & Pollard, 2006).

Another major cell type found in A4 high specimens is t-cells. T-cells play a critical role in immune responses in BC(Kos & de Visser, 2021). They are involved in anti-tumor immunity. High levels of CD8+ve t-cells through PD-1/PD-L1 inhibition restore the anti-tumor activities of t-cells(Tietscher *et al*, 2023). The cell type distribution and characteristics were reflected in the cell cycle phase distribution of A4 high and low specimens (**Fig. 5D**). While A4 low specimen comprised of 41% cells in G1 phase, 37% cells in S phase, and 23% cells in G2 phase, A4 high specimens contained cells that were distributed at 68% in G1 phase, 1.3% is S phase, and 31% in G2 phase of the cell cycle. .

To validate the 1388 genes signature (**Fig. 2C**) in scRNA seq data, the genes that are increased in A4 low specimens (gene signature 2) were loaded in snRNA seq data (**Fig. 5E**). The output as shown in the Umap matched the bulk RNA seq heat map with the genes highly enriched in basal cell population (cluster 1) with minimum expression in A4 high population. No major crossover was observed in the signature between the A4 low and high specimens. Similarly, the gene signature for each cluster plotted in Umap showed limited crossover between clusters (**Fig. 5F** and **EV5C**).

The A4 low and high gene signatures from snRNA seq were used to determine the enriched pathways using GSEA hallmark oncogenic pathways (**Fig. 5G**). High A4 gene signature exhibited 37 statistically significant pathways, all of which were downregulated. Low A4 gene signature is associated with 26 pathways, with all of them upregulated. All these pathways are oncogenic, suggesting the cell signature of A4 high corresponds to oncogenic inhibition. Seventeen pathways were shared between A4 high and low cells (regulated in opposite directions). Interestingly, the pathways (RAF, MEK, KRAS, and others) are primarily enriched, suggesting a unified control of the oncogenic MAPK pathway. Collectively, the snRNA seq data is concordant with the bulk RNA seq and provides additional information that facilitate the interpretation that BC cells with high A4 signature have favorable molecular profile and disease prognosis. The snRNA seq data was derived from two A4-high and one A4-low ER-positive, PR-positive, and AR-positive specimens and hence the outcome on immune infiltration, stromal composition, and pathway alteration are exploratory and illustrative rather than definitive, and these outcomes need to be validated in a larger patient specimen cohort."

7. *A recurring conceptual tension in the manuscript concerns whether intratumoral A4 levels reflect tumor-intrinsic regulatory states or broader systemic and metabolic influences. Although the authors argue convincingly against reliance on circulating hormone measurements, intratumoral A4 may still be shaped by stromal content, local steroidogenic enzyme expression, or tissue composition differences between low-grade and high-grade tumors. The enrichment of fibroblasts and endothelial cells in A4-low tumors further complicates interpretation. The manuscript would be strengthened by explicitly discussing how tumor purity, stromal fraction, and intracrine enzyme expression might confound or contribute to measured A4 levels*.

   Response: We thank the reviewer for this valid comment. We have included a paragraph at the end of the discussion to point out to the limitations of this work. The paragraph reads:

   > "Some of the limitations of this work include smaller cohort size, tumor purity, stromal fraction, fibroblast enrichment, endothelial cell population, and intratumoral steroidogenic enzyme expression, all of which have the potential to alter the predictive properties of A4-dependent signature and the disease outcome. Additionally, longitudinal specimen utilization will help in understanding the relationship of this signature to progression of the disease. Further validation using potential pure cellular population and extensive quantification of enzymes and other hormones will facilitate the confirmation of the findings in this manuscript."

8. *The Discussion positions A4 and the associated gene signature as having substantial diagnostic, prognostic, and therapeutic potential, including possible use as a companion diagnostic or predictor in benign tissue. These claims are ambitious relative to the evidence presented. The study does not assess longitudinal risk, treatment response, or predictive performance in clinical decision-making contexts. Moreover, proposing A4 as a therapeutic agent is conceptually problematic given its dual androgenic and estrogenic potential. A more restrained and evidence-aligned discussion of clinical implications would improve credibility and avoid overextension beyond the current data*.

   Response: We have now removed any mention of the therapeutic benefit of A4. We agree with the reviewer that the findings are correlative and not causation. Also, we have included a sentence in the discussion regarding this point. The sentence reads;

   > "Additionally, longitudinal specimen utilization will help in understanding the relationship of this signature to progression of the disease."

9. *The functional assays presented in Figure 6 are intended to provide mechanistic support for the clinical observations, but in their current form they raise several concerns regarding experimental design, quantification, and interpretability. First, the AR transactivation assays (COS7 and T47D) appear under-sampled across the concentration range. The dose-response curves are fitted with a limited number of concentration points, particularly around the inflection region, making it difficult to reliably determine EC50 values or compare relative potency between A4 and DHT. Without sufficient data density in the dynamic range of the response, it is unclear whether the apparent shifts in activation reflect true differences in receptor activation or are artefacts of curve fitting. These assays should be repeated with additional concentrations included. Second, the proliferation assays raise questions regarding statistical analysis and biological relevance. In the endpoint SRB assay, the magnitude of the reported effect appears modest, yet statistical significance is claimed without a clear description of the statistical framework, sample size (biological vs technical replicates), or multiple-comparison correction across doses. Please, all of this should be disclosed and an additional cell line should be taken in these assays. Relatedly, the IncuCyte growth curves show only subtle separation between conditions, despite strong statistical claims. Given the visual overlap between curves, additional information is required on how statistical testing was performed for time-course data (e.g. area under the curve, mixed-effects models, or endpoint comparisons), and whether effect sizes are biologically meaningful. Improved visualization or complementary quantification would substantially strengthen this analysis. Additionally, another cell line should be used to confirm the effects. Third, the AR knockdown experiments require some changes. While AR siRNA abolishes the A4-dependent effect, it is unclear what the baseline effect of AR knockdown alone is on proliferation. Specifically, it is not evident whether the data are normalized to non-silencing siRNA within each treatment condition, or whether AR depletion itself alters baseline growth independently of A4. That is to solve this please show the non normalised data; as then we will be able to see the effect of AR knockdown as well*.

Response: We thank the reviewer for this critical review of the mechanistic studies. We have addressed the comment with the following evidences;

1. The AR transactivation studies were repeated with half-log doses to ensure that we obtain appropriate EC50 numbers (**Fig. 6A**). The new figure is pasted below for the convenience of the reviewer.

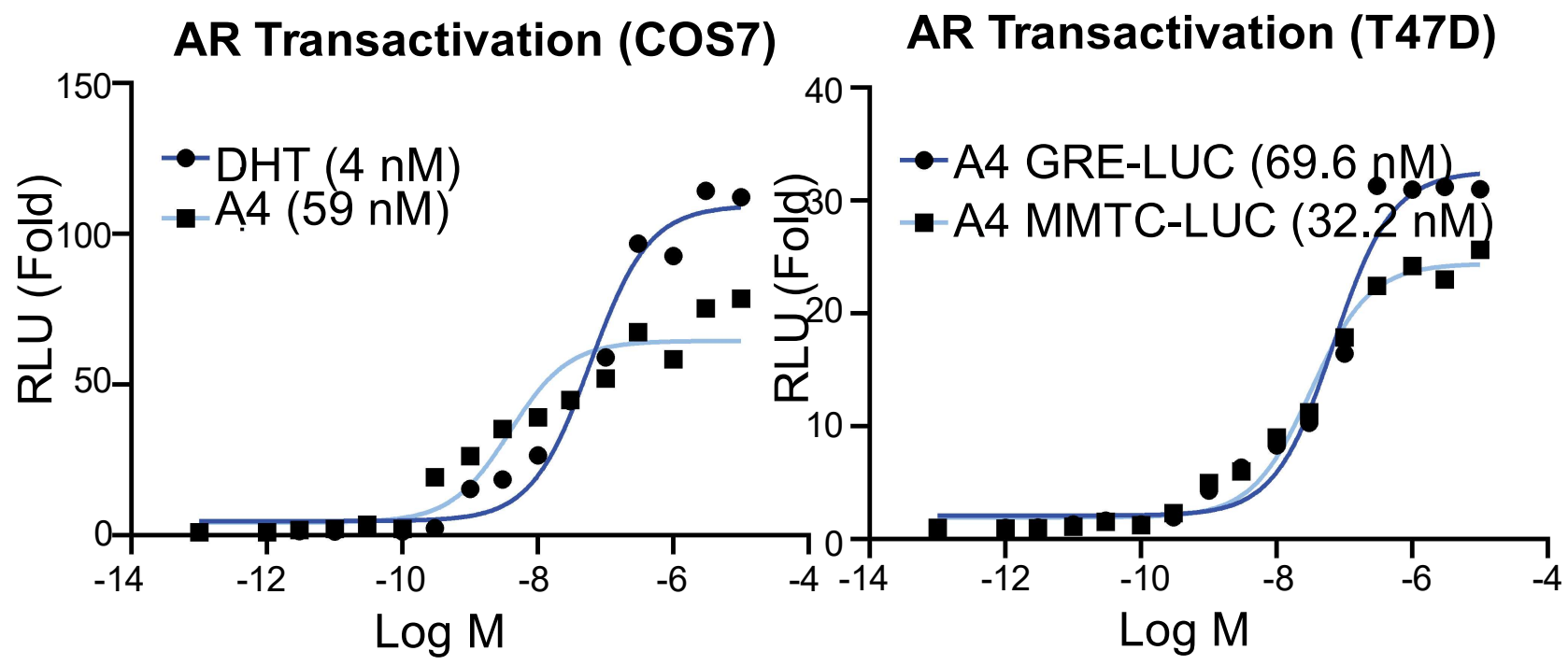

2. A4 inhibited proliferation in ZR-75-1 was about 40%. We have repeated the experiment in ZR-75-1 and T47D using incucyte. The data is provided below. It confirms the anti-proliferative effect of A4 (**Fig. 6C**).

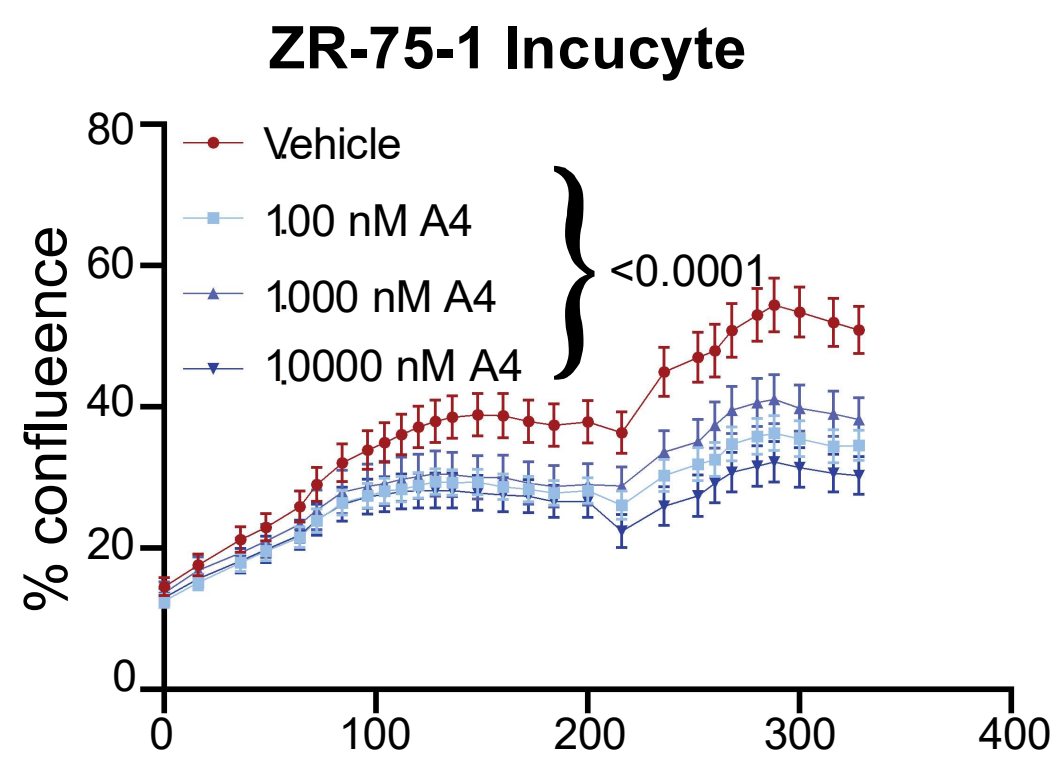

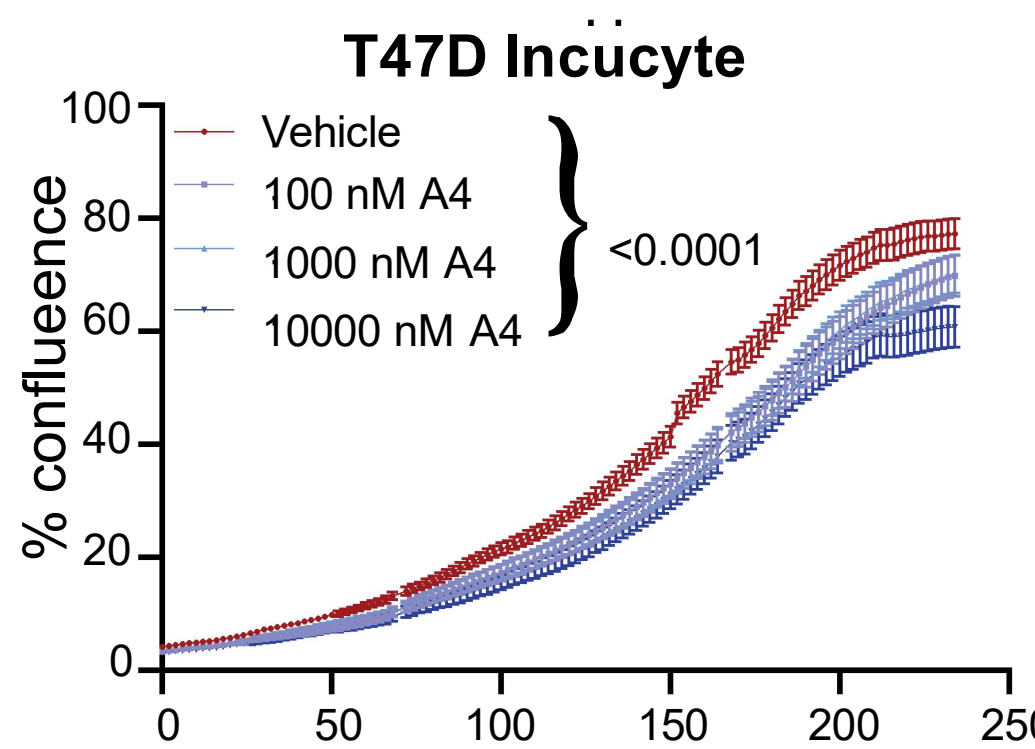

3. The data are normalized to the respective siRNA. The non-specific siRNA normalized data is provided below. This shows a modest difference in the proliferation of cells due to AR knock-down.

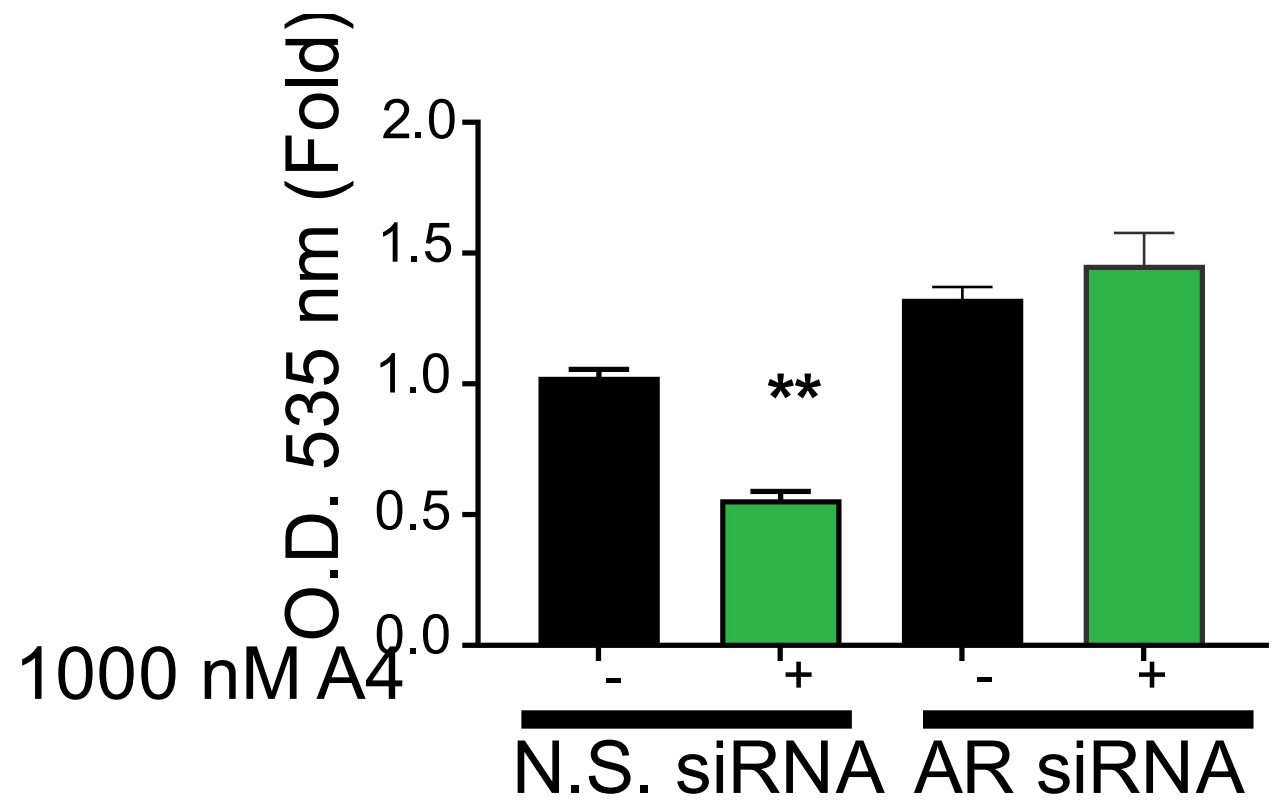

Reviewer-2: We thank the reviewer for providing these comments to improve the quality of the manuscript. We hope we have addressed the comments to the satisfaction of the reviewer.

1. *The title of the article needs to be changed. 4-AD cannot be a tumor suppressor since this term is used for genes and not small molecules. The title should read "androstene-3,17-dione" suppresses hormone receptor positive breast cancer*.

   Response: We thank the reviewer for this suggestion. We have revised the title as suggested by the reviewer. We are providing an alternate title. I hope the reviewer will agree with this new title. The new title of the manuscript is

   "Intratumoral androstene-3,17-dione Defines Breast Cancer Subtype and Prognosis"

2. *In hormone positive breast cancer the prevailing view has been that 4-AD would be converted to estrone by aromatase and this would be pro-proliferative. The authors suggest that this pathway may be by-passed by activation of the AR. To make this case the authors need to measure aromatase levels and perhaps E1 as well*.

   Response: This is a valid point raised by the reviewer. We measured the estrone levels in the samples (**Dataset EV2**). The average estrone levels in the three groups are 1.46, 2.02, and 1.49. We found no difference between the groups. Also, we performed extensive analysis with estrone and found no pattern of differences between specimens nor any clustering between specimens. We have included the following sentences in the manuscript;

   > "We also measured estrone levels and aromatase gene expression and found no correlation between any clinical variable, gene expression, estrone levels. Hence, estrone was not taken into consideration for further analysis."

We did further analysis comparing estrone vs androstenedione, aromatase vs androstenedione, and aromatase vs estrone. We did not find any correlation between any of these comparators. The analysis outcome is provided below.

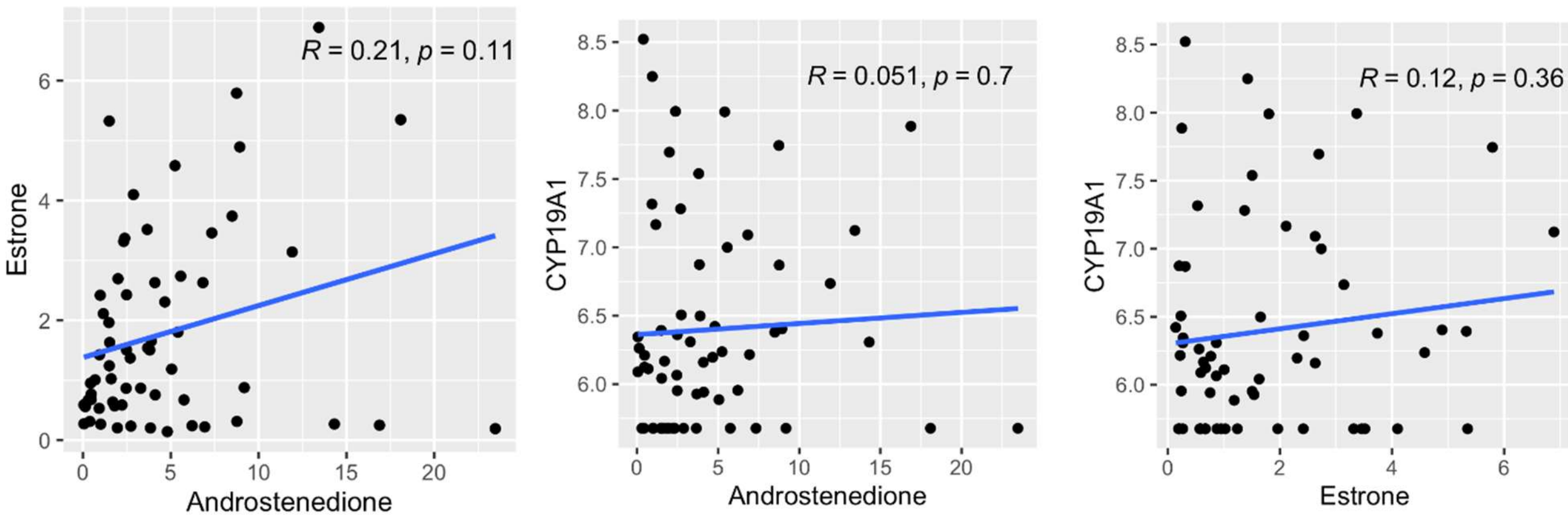

3. *The authors show a large dynamic range of DHEA,4-AD and T in the tumor specimens and classify these levels from - 4 to +4. The authors should provide the steroid levels that correspond to these ordinal values. As these levels can vary by more than 100-fold do the authors have sufficient power to from meaningful gene clusters*?

   Response: We thank the reviewer for this comment. We would like to clarify that the −4 to +4 values shown in the figures do not represent ordinal categories. The steroid measurements were treated as continuous variables. Because steroid levels exhibited somewhat right-skewed distributions (**Response Fig 2-3A**), the raw values were first log-transformed (**Response Fig 2-3B**) and then standardized to have mean 0 and standard deviation 1 (**Response Fig 2-3C**). Therefore, the values shown in the manuscript represent standardized z-scores of log-transformed steroid concentrations, which typically range between approximately −4 and +4.

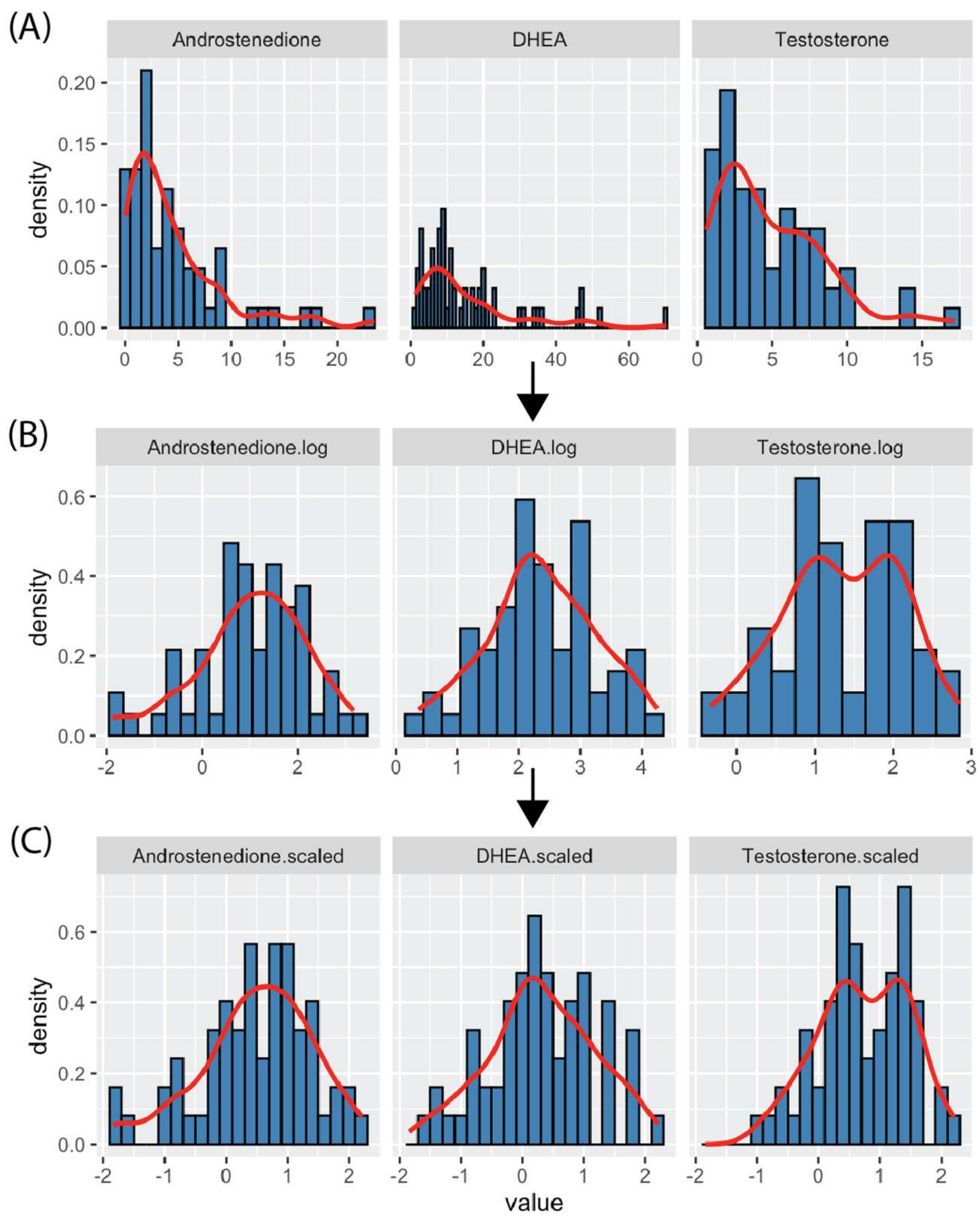

Response Figure 2-3.

We have clarified this in the Results to avoid confusion:

> "Steroid concentrations were log-transformed to reduce skewness and then standardized to z-scores (mean = 0, standard deviation = 1) for downstream analyses. Therefore, the values shown in the figures represent standardized log-transformed steroid levels."

4. *The measurement of hormone levels is crucial data for the manuscript. The authors describe what appears to be a nice GC-MS method with stable isotope internal standards. However, more details of this method should be given and how the authors compensated for any matrix effects from the tumor samples. Also, the authors should provide LLOQ, LLOD and precision and accuracy estimates, when analyzing the tissue samples*.

Response: We have now provided additional details of the method used to measure the hormones. The following is the detailed description of the methods;

> "Intratumoral hormone levels were quantified based on previous reports(Bhardwaj *et al*, 2023; Han *et al*, 2021). Gas chromatography-mass spectrometry (GC-MS)-based steroid profiling was performed using a Shimadzu GC 2010 Plus gas chromatograph interfaced with a triple-quadrupole Shimadzu GCMS-TQ8050 (Shimadzu Co., Kyoto, Japan). In brief, 5 mg of BC specimens were cut and added to 1 mL 0.2 *M* phosphate buffer (pH 7.2) and 20 µL of a mixture of internal standards (d_8-17α-hydroxyprogesterone, 1 µg/mL; d_6-DHEA and d_9-progesterone, 0.5 µg/mL; d_3-testosterone and d_4-cortisol, 0.25 µg/mL; d_4-17β-estradiol, 0.2 µg/mL). After pulverizing the tissues twice with TissueLyser II (Qiagen; Hilden, Germany) for 10 min at 25 Hz with three zirconia beads (3.0 mm I.D., Toray Industries, Tokyo, Japan), the samples were centrifuged for 5 min at 12,000 rpm, and the supernatant collected. The samples were then loaded on a preconditioned Oasis HLB SPE cartridge. The cartridge was washed twice with 1 mL of water and eluted with 2 mL of methanol and 2 mL of 90% methanol. The combined eluates were evaporated under nitrogen at 40°C. Then, 1 mL of 0.2 *M* acetate buffer (pH 5.2) was dissolved for additional extraction. After two extractions with 2.5 mL of methyl *tert*-butyl ether, the organic solvent was collected, evaporated under nitrogen at 35°C, and further dried in an amber vacuum desiccator with P_2O_5/KOH for at least 30 min. Finally, the dried residues were derivatized in 40 µL of MSTFA/NH_4I/DTE (500:4:2, *v/w/w*) for 20 min at 60°C, and 2 µL of the resulting mixture was analyzed with GC-MS in the collision-induced dissociation mode.
>
> Method validation was performed accordingly to the US Food and Drug Administration 2018 guideline for bioanalytical method validation [US Food and Drug Administration. (2018) *Guidance for Industry: Bioanalytical method validation*, No. FDA-2013-D-1020, 1–41]. The overall imprecision (%*CV*) and accuracy (%*bias*) for DHEA, A4, testosterone and DHT ranged from 0.8–19.9% and 69.9–111.1%, respectively, while the lower limit of quantification (LLOQ) was 0.1–0.25 ng/mL (Han *et al*., 2021)."

5. *The authors show almost equal potency between DHT and 4-AD in reporter gene assays in COS-7 and T47D cells. Have the authors investigated whether 4-AD recruits different coactivators to the AR? This could be supported by the statement on p12 which found only 2 genes significantly correlated with DHEA and T levels but more with 4-AD*.

Response: We thank the reviewer for this suggestion. We performed a ChIP-PCR experiment with various coactivators. We have incorporated this data in **Figure 6E**, which is provided below for reviewer's convenience.

This figure and text referred to data that was removed from the final version of the manuscript.

6. *The authors should cite the work of the McDonnell laboratory (Safi et al. Nature Communications) which showed that high levels of androgens in the prostate cause AR dimerization and do not promote growth but instead cause cell differentiation; and that low levels of androgens working through monomeric AR in the cytosol promote growth . Could a similar mechanism explain the authors findings*?

   Response: We thank the reviewer for bringing this reference to our attention. We have now added the reference from Dr. McDonnell's group. We have also included the following section in the manuscript in relation to this finding;

   > "Alternatively, a recent work from Dr. McDonnell's laboratory demonstrated that low intratumoral androgen levels in prostate cancer function through AR monomer that activate mTOR signaling pathway and increase prostate cancer cellular proliferation, while high androgen levels function through AR dimer that result in c-Myc oncogene inhibition and inhibition of proliferation(Safi *et al*, 2024). This mechanistic study explains the biphasic androgen function in prostate cancer that have demonstrated that treatment with supraphysiological doses of androgens will result in an inhibition of tumor growth(Chatterjee *et al*, 2019). It is likely that the A4 in BC could function by a similar mechanism. **Figure 2B** shows that high A4 specimens have decreased mTOR and c-myc oncogenic signals, potentially validating the findings made in prostate cancer."

7. *Is there any evidence that AR can regulate ER and PR expression in breast cancer*?

Response: Our work (Asemota S et al., 2024 and Ponnusamy S et al., 2019) have shown that activating AR inhibits ER and PR function by dissociating ER binding to EREs. Activated AR also competes with ER for FOXA1 and coregulators, all of which inhibit ER function. Since PR is an ER target gene, PR expression is inhibited by activated AR. However, no clear evidence is available for the regulation of ER expression by AR. Our studies in patient-derived xenografts and cell line xenografts where AR activated by agonists such as DHT did not alter ER expression, but altered ER function.

8. *The authors indicate that 1388 genes highly correlate with 4-AD; and then show that 4-AD levels correlate with gene expression. This seems like a circular argument and should be clarified.*

   Response: We thank the reviewer for raising this important point regarding potential circularity. We would like to clarify that the goal of this analysis was not to test whether clusters differ in A4 levels. As the reviewer pointed out, because genes were selected based on correlation with a continuous A4 measurement, separation of tumors along the A4 axis is expected. Therefore, the key question is not whether clusters differ in A4, but whether tumors segregate into reproducible transcriptional states within the A4-associated gene space and these states are associated with independent clinical and pathological variables. Importantly, hormone measurements were not used in the clustering algorithm, but only used post hoc for biological interpretation of the resulting transcriptional groups.

   We have clarified this in Results to avoid impression that clustering was performed to rediscover A4 differences. The section reads

   > "A4-correlated genes form different transcriptional states across tumors. Unsupervised k-means clustering algorithm was applied to the 1388 genes whose expression significantly correlated with A4 (**Fig. 2C**). This clustering resulted in three tumor groups and two gene clusters, with gene expression patterns changing in a gradual manner across the three tumor groups, suggesting a continuous transcriptional gradient. Because genes were selected based on correlation with A4 levels, separation of tumors along the A4 axis is expected. The three transcriptional groups corresponded to tumors with low, intermediate, and high A4 levels, and were therefore labeled low-, intermediate-, and high-A4 clusters. DHEA and testosterone levels showed similar increasing trends across clusters (although testosterone was not statistically significant), suggesting that A4-dependent clusters and genes in the clusters promote broad androgenic clustering (**Figs. 2C-D**)."

9. *The authors show collectively that high 4-AD specimens favor a better prognosis and therapy response while the low 4-AD specimens indicate aggressive growth and*

proliferation and then go on to indicate how these two groups differ in cell cycle phase. The differences in cell cycle phase are given as a single percentage. Should this not be a range given the range of 4-AD levels?

Response: We discussed with the scRNA seq and flow cytometry experts and also with computational biologists. They all indicated that the percent of cells in a cell cycle phase is typically a single number (number of cells in a phase/total number of cells), unless the experiment is repeated multiple times to obtain a range.

Minor comments:

10. *Please provide the sources of all plasmids including the hAR*.

    Response: We have now included the source of all plasmids in the reagents and tools table in the methods section. We apologize for the oversight.

11. *Do not use the tern "dead" patients please change to "deceased"*.

    Response: We thank the reviewer for this correction. We searched for "dead" and replaced with "deceased".

Reviewer-3: The authors thank the reviewer for the favorable comment that this work is important and understudied. We also thank the reviewer for the comments to strengthen the manuscript. We have addressed all the comments provided by the reviewer.

1. *Framing of prior literature and abstract statement The opening statement of the abstract - "The role of androgens and androgen receptor in breast cancer in the clinic has been defined by correlating circulating androgen levels to clinical variables" - is misleading. There is extensive preclinical literature as well as clinical trial data addressing AR signaling in breast cancer, and it is well established that AR biology differs fundamentally between ER-positive and ER-negative disease. This statement should be revised to accurately reflect the field and to clearly position the present study in relation to existing mechanistic and clinical work*.

   Response: We thank the reviewer for pointing out to the literature background and our claim. We have now revised these sections. The following are the different sections that were revised;

   > "Preclinical and clinical evidences suggest that androgens inhibit breast cancer (BC) growth in estrogen receptor (ER)-positive BC and promote growth in ER-

negative BC. Clinically, the subtype-selective role of androgens in BC could be confirmed by correlating circulating androgen levels to clinical variables"

"Clinical studies to determine the role of estrogens and androgens in BC failed to take into consideration the local conversion by steroidogenic enzymes(Hong & Chen, 2011; Pasqualini & Chetrite, 2005; Song *et al*, 2006) and expression of free serum hormone binding globulin (SHBG)-unbound hormone, all of which have resulted in confounding results(Albers *et al*, 2024; Brennan *et al*, 1973; Bulbrook *et al*, 1971; Dorgan *et al*, 1996; Secreto *et al*, 1984; Wang *et al*, 1975)."

2. *Biological hypothesis and mechanism*
It remains unclear whether the authors hypothesize that androstenedione exerts tumor-suppressive effects via:
o AR activation and downstream anti-proliferative gene programs, or
o an alternative, AR-independent intracrine or paracrine mechanism.
If AR-mediated effects are proposed, this is neither novel nor directly demonstrated in the current study. If a different mechanism is envisioned, it should be clearly articulated in the Introduction and supported by appropriate analyses. At present, the mechanistic model underlying the key conclusions is insufficiently defined.

Response: We thank the reviewer for this comment. We took multiple approaches to address this comment. A) performed a pathway analysis of the 90 gene signature; b) performed ChIP-seq in A4 high and low specimens; and c) performed gene expression pathway analysis of in vitro RNA sequencing data. We have now incorporated these analyses in the manuscript.

This table and text referred to data that was removed from the final version of the manuscript.

Additionally, “we examined overlap between the larger set of A4-associated genes and AR-target genes and established androgen-related gene sets. Among the 1,388 A4-associated genes, 98 overlapped with androgen-related gene sets curated in MSigDB, including 11 genes present in the HALLMARK_ANDROGEN_RESPONSE gene set (CDK6, HMGCS1, LIFR, MAP7, PIAS1, PLPP1, PTPN21, SAT1, SLC38A2, SMS, TPD52). These data suggest that there is an enrichment of androgen-dependent signature”. The above information within quotes has been incorporated in the manuscript.

The ChIP-seq studies demonstrated that AR is recruited to its downstream target gene family myocyte enhancer factor (MEF2). Although androgen response elements (AREs) are not one of the motifs AR was recruited to, the AR is recruited to its downstream target genes’ regulatory regions. The section on ChIP seq has been incorporated in the manuscript and provided below for the reviewer’s convenience.

This figure and text referred to data that was removed from the final version of the manuscript.

In vitro RNA seq GSEA analysis showed that androgen response pathway is one of the pathways enriched in A4-treated cells, while estrogen-responsive pathways are enriched in vehicle-treated cells, which is in concordance with our previous publications. The section is provided below for the reviewer's convenience.

> "Gene set enrichment analysis of the genes showed that androgen response is one of the hallmark pathways enriched in the A4-treated cells. In addition, estrogen-responsive pathways are enriched in vehicle-treated cells, concordant with our earlier publications that showed treatment of ER-positive BC tumors with androgens inhibits estrogen pathway(Asemota *et al.*, 2024; Ponnusamy *et al.*, 2019a)."

This figure and text referred to data that was removed from the final version of the manuscript.

3. *Hormone measurements and tissue intracrinology*
   Given that tumor intracrinology remains poorly characterized, more detail on the technical and biological aspects of tissue hormone measurements is essential. In particular:
   o Which tissue-related or pre-analytical factors affect intratumoral hormone levels?
   o Were any samples excluded due to hormone measurement quality, analogous to the RNA-seq exclusions due to RNA integrity?
   o How robust are A4 measurements across tissue processing conditions?
   Greater transparency here is critical for interpreting the correlations reported.

Response: We did not exclude any samples based on hormone measurement quality. The only exclusion criterion was the availability of corresponding transcriptomic data. Samples with hormone measurements but without matched transcriptomic data were excluded from the integrative analyses. Specifically, in the discovery cohort, 73 specimens were originally collected for intratumoral hormone measurements, of which 62 had matched transcriptomic data and were included in the analysis. In the validation cohort, 88 specimens had hormone measurements, and 77 with matched transcriptomic data were included in the analysis.

We have the following text in Methods:

> "BC patient specimens were collected under protocols approved by the University of Tennessee Health Science Center (UTHSC) institutional review board (IRB) (IRB protocol numbers 14-03113-XP and 20-07769-XP). Seventy-three specimens were collected for intratumoral hormone measurements and transcriptomics. Since only 62 specimens' transcriptomic data met the quality control benchmark, 62 specimens' data were used for analyses. For the validation cohort, 88 specimens were collected. Transcriptome data from only 77 specimens met the benchmark and hence the validation cohort contained data from 77 patient specimens."

4. *Context of estrogen signaling*
   The manuscript addresses an important question and provides intriguing correlative data on intratumoral androstenedione in ER-positive breast cancer. However, clearer mechanistic framing, improved methodological transparency, and additional analyses are required before the conclusions can be fully supported. In light of the extensive ER/AR interactions it is of interest to consider the findings in the context of ER signalling activity and estrogen levels.

   Response: We have extensively shown in our prior works that AR activation interferes with ER signaling (Asemota S et al and Ponnusamy S et al). This is also observed in our in vitro RNA seq data that showed downregulation of ER signaling by A4.

   We have now provided all methodology adopted to analyze the data.

5. *Patient samples are selected "randomly" reflecting the full spectrum of patients, while this has some attraction, it is well established that the biology of hormone receptor signaling is very different between different tumor subtypes. As such hypotheses should be testable within specific subtypes. While the authors distinguish ER+ and ER- tumors, molecular subtypes should be taken into consideration*.

   Response: We thank the reviewer for this important comment. We agree that hormone receptor signaling biology differs across breast cancer subtypes and that it is important to

evaluate hypotheses within subtype-specific contexts. While our cohort was not pre-stratified by subtype at the design stage, we performed additional data analyses to explicitly address this point.

In the TCGA BRCA cohort, we derived PAM50 intrinsic subtypes (luminal A, luminal B, basal-like, HER2-enriched, and normal-like) and evaluated their relationship with A4-associated gene expression. We also computed a PAM50-based proliferation score using the proliferation-associated genes within the PAM50 gene set. Consistent with our observations using MKI67, A4-associated clusters were associated with proliferation (**Fig. 4H**). Subtype distribution analysis showed that the A4-low cluster was enriched for Basal-like and Luminal B tumors, whereas the A4-high cluster was enriched for Luminal A, with the A4-intermediate cluster showing a mixed composition (**Fig. 4I**). These results indicate an association between A4 clusters and PAM50 intrinsic subtypes. However, each A4 cluster contained a mixture of subtypes, indicating that the A4-associated classification does not simply recapitulate intrinsic subtype.

Importantly, we further assessed the association within subtype by stratifying analyses by ER status and PAM50 subtype. We observed that A4-associated differences were most pronounced in ER-positive tumors, where A4 clusters stratified clinical outcomes even within the same subtype, particularly within Luminal A tumors (**Fig. 4J**). In contrast, this pattern was not observed in ER-negative tumors. These results demonstrate that the association between A4-associated gene expression and tumor characteristics can be evaluated within subtype and is not solely driven by differences between subtypes. These results suggest that the 90-gene signature reflects androgen-related tumor biology that is related to, but not identical to, known proliferation and intrinsic subtype-associated expression patterns, and provides additional prognostic dimension beyond these established markers.

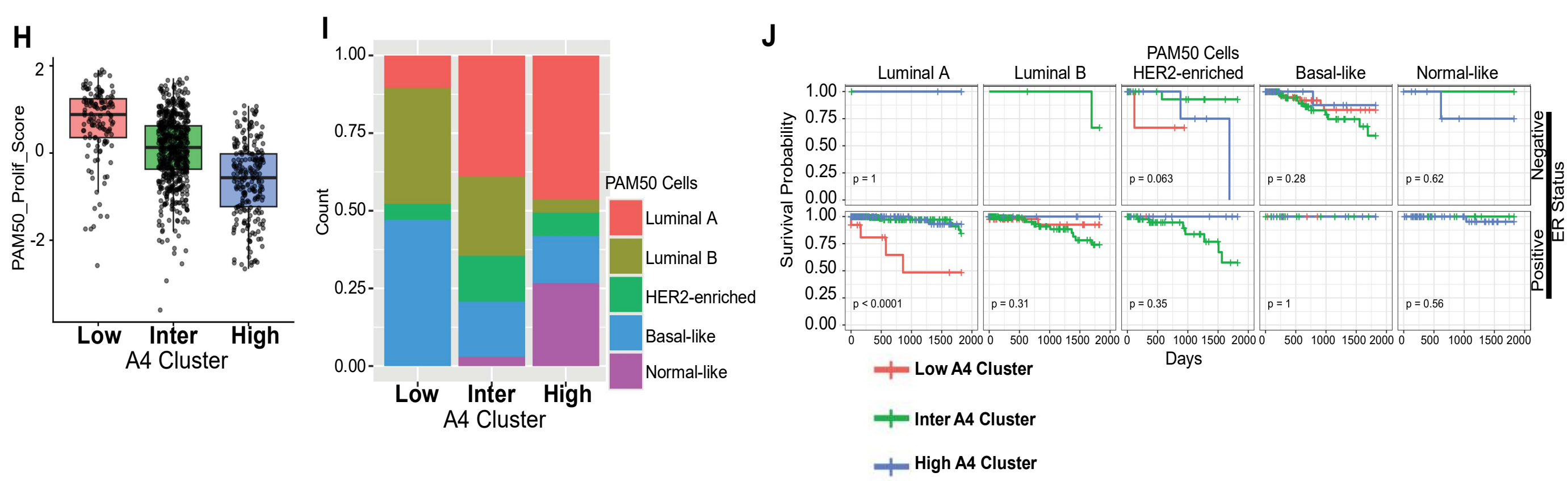

Figures 4H-J.

We have clarified this point in the revised manuscript:

"To assess the relationship between A4-associated gene expression and established BC classifiers, we evaluated PAM50 intrinsic subtypes and a PAM50-based proliferation score in the TCGA BRCA cohort. A4 clusters were significantly associated with proliferation inhibition, consistent with analyses using MKI67 (**Fig. 4H**), yet each cluster contained a mixture of subtypes (**Fig. 4I**). Notably, survival analyses stratified by ER status and PAM50 subtype showed that A4 clusters further stratified outcomes within the same subtype. It was most prominent within Luminal A where A4-low tumors exhibited worse survival than A4-high tumors in ER-positive tumors (**Fig. 4J**). These findings indicate that androgen-associated gene expression could capture additional prognostic dimension beyond conventional subtype classification."

Minor:

6. *To assess cell proliferation by RNAseq, the authors use KI67. Better to use the combined 11 or 14 proliferation genes from the PAM50 signature*.

   Response: We thank the reviewer for this helpful suggestion. We agree that a multi-gene proliferation score, such as the PAM50 proliferation signature, can provide a more robust transcriptomic measure of proliferation than MKI67 alone. In response, we evaluated the PAM50 proliferation score in our cohort and found that it was highly correlated with Ki67 as well as MKI67, indicating that Ki67 served as a reasonable proxy for proliferation in this dataset. Because the results were concordant and Ki67 or MKI67 is a widely used and clinically interpretable proliferation marker in breast cancer, we elected to retain Ki67 and MKI67 in the main manuscript for simplicity and consistency of presentation. We are happy to provide the comparison plot to the editor or reviewers if helpful.

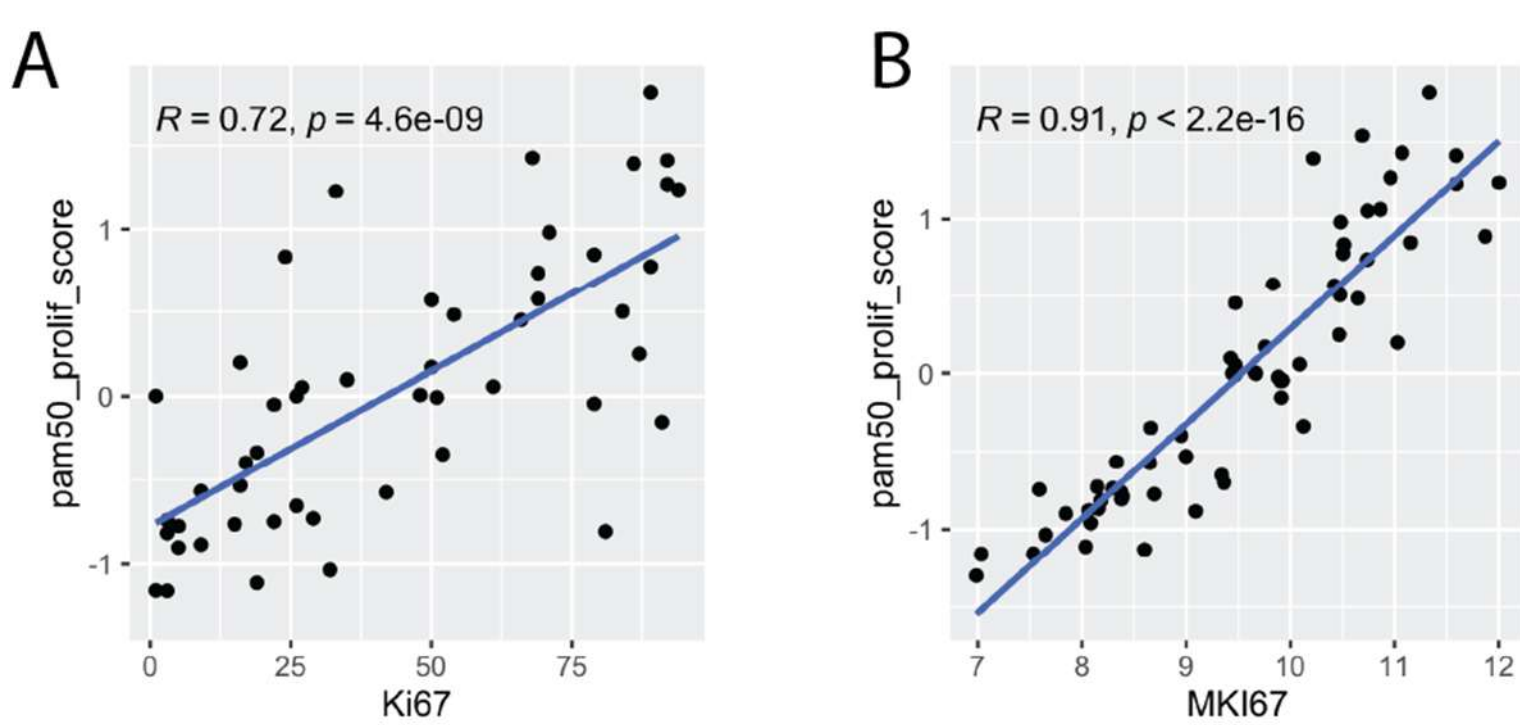

Response Figure 3-6.

15th Jun 2026

Dear Dr. Narayanan,

Thank you for submitting your revised manuscript to EMBO Molecular Medicine, and please accept my apologies for the delay in getting back to you. As referee #2 was unavailable to assess the revision, referee #1 kindly agreed to evaluate your responses to the comments from both referees #1 and #2 (see below). Unfortunately, we were also unable to obtain a follow-up assessment from referee #3. Referee #1 therefore generously agreed to review your responses to referee #3's comments as well. Their additional evaluation is reproduced below:

"I looked again at the revised manuscript, specifically with respect to how the authors addressed Reviewer 3, keeping in mind what my previous comments already covered. My assessment is that the authors have made a meaningful effort, but the response to Reviewer 3 is only partially satisfactory. The revised framing of prior androgen/AR literature is improved, and the added PAM50/subtype analysis is useful, but the central mechanistic concern remains insufficiently resolved. Even the revised framing remains somewhat stronger than warranted, because the manuscript still tends to present the intratumoral A4/transcriptome approach as defining the clinical role of androgens, whereas the data more conservatively support an association between A4-linked transcriptional states and tumour phenotype. The authors now add androgen-gene-set overlap, in vitroA4 RNA-seq, AR ChIP-seq, and FKBP5 ChIP-qPCR, yet these analyses still do not clearly distinguish whether the clinical A4-associated transcriptional state is directly AR-driven, indirectly linked to AR signalling, or mainly reflects a broader differentiation or intracrine state. This is particularly important because the AR ChIP-seq/motif analysis is interpreted too strongly; MEF-family motif enrichment at differential AR-bound regions is interesting, but it does not demonstrate MEF2 activity, MEF2 dependency, or a causal AR-MEF2 mechanism. I also think Reviewer 3's concerns about intratumoral hormone measurement were not fully addressed: the authors clarify sample inclusion, but still do not sufficiently explain how tissue heterogeneity, matrix effects, pre-analytical variability, and robustness of A4 measurements across processing conditions were handled. The estrogen-signalling point is also only partly answered, mostly by reference to prior work and in vitro data. Finally, the PAM50 analysis addresses subtype composition descriptively, but it does not establish that the A4 signature has independent prognostic value beyond subtype, proliferation, and standard clinical variables. Overall, I would say the revision is improved and does not require a complete reframing, but I would still ask for focused tightening before acceptance. In particular, the limitations should be made more specific and better integrated into the interpretation of the results. At present, these issues are partly acknowledged in the Discussion, but the strength of the language in the Abstract, Results, Paper Explained, and figure legends is not always true to those limitations.

This is broadly in line with my assessment of how the authors addressed the other reviewer comments as well. They have made a clear effort to respond, but in several places I do not think the additional data are sufficient to justify retaining the strength of the mechanistic and clinical claims currently made in the manuscript."

We have carefully considered the referee's feedback. We appreciate, as does the referee, the substantial effort that has gone into revising the manuscript and addressing the concerns raised during peer review. Nevertheless, several issues remain that require further revision. Importantly, these concerns appear to be addressable without additional experimentation, through clearer descriptions of the methods and results, and a more accurate and balanced presentation of the conclusions supported by the data.
On this basis, we would like to invite a further revision of the manuscript. Please note that, at EMBO Press, we typically allow only one round of major revision. Accordingly, this will be the final opportunity to address the outstanding concerns raised during peer review.

In addition, please address the editorial matters listed below:
- Background should be changed to Introduction.
- The manuscript sections should be in the following order: Title page - Abstract - Introduction - Results - Discussion - Methods - Data Availability - Acknowledgments - Disclosure Statement & Competing Interests - References - Figure Legends - (Main Tables with legends if applicable) - Expanded View Figure Legends.
- Ethics approval and consent to participate should be placed somewhere in the Methods.
- Declarations and Consent for publication should be removed.
- Rename "Availability of data and materials" to "Data Availability Section" and place it before the Acknowledgements.
- Remove author contributions from the manuscript. You will be asked to provide CRediT (Contributor Role Taxonomy) terms in the submission system. These replace a narrative author contribution section in the manuscript.
- Eric Muirhead Chair of Excellence, and the UTHSC Center for Cancer Research: the funding info in the manuscript needs to be provided under Acknowledgments so no separate Funding section should be used.
- Figure EV1 should be made Table EV1.
- Supplementary methods should not be used.
- 4 datasets are uploaded, and there is also a file with legends, which is not needed since each legend needs to be added to its corresponding Excel file, as a separate sheet/tab.

- The visual abstract needs reformatting as it is too large and has a large white border; the image should be exactly 550 pixels wide x 300 pixels high.
- The reagent and tools table should be removed from the manuscript and uploaded as a separate file.
- Please provide the Source Data as one zip file per figure, with a subfolder for each figure panel.
- Please address the queries from our data editors in the figure legends:
o Please note that the exact p values are not provided in the legends of 4J; 6B-D; EV-5B,C.
o Please indicate the statistical test used for data analysis in the legends of figures 2B; 3C; 4J; 6D; EV-4I; EV-5B,C.
o Please note that the box plots need to be defined in terms of minima, maxima, centre, bounds of box and whiskers, and percentile in the legends of figures 2DE,F,G; 3B; 4B,C,F,G,H; EV-2G; EV-3B; EV-4G.
o Please note that information related to n is missing in the legends of 2D,E,F,G; 3B; 6B,C,E; EV-2G; EV-3B; EV-5B,C.
o Please note that the error bars are not defined in the legends of figures 6B,C,D,E; EV-4C,E.

I look forward to seeing a revised form of your manuscript as soon as possible. Use this link to login to the manuscript system and submit your revision: https://embomolmed.msubmit.net/cgi-bin/main.plex

With kind regards,

Lise Roth

Lise Roth, Ph.D
Senior Editor
EMBO Molecular Medicine

***** Reviewer's comments *****

Referee #1 (Comments on Novelty/Model System for Author):

Technical quality:
The biology and multi-modal design are strong, but the statistical framework is mostly descriptive and association-based. The main clinical analyses rely heavily on unadjusted correlations, Kruskal-Wallis/Fisher tests, Kaplan-Meier curves, and stratified visual comparisons, rather than models that formally adjust for obvious confounders such as ER status, PAM50 subtype, proliferation, grade/stage, race, age, and tumor purity/stromal composition. This matters because the A4 axis is tightly entangled with subtype and proliferation. The authors partly acknowledge the circularity of selecting A4-correlated genes and then clustering on them, but the downstream clinical claims still depend on those derived groups. Their sPLS/90-gene analysis is useful as an internal feature-reduction exercise, but it is not a proper clinical prediction validation: the final model is fit in a small discovery set, the LOOCV is mainly used to argue gene-set stability, and the TCGA analysis validates inferred cluster behavior rather than measured intratumoral A4. The survival analysis is also statistically thin; Kaplan-Meier stratification by ER/PAM50 is informative, but it does not establish independent prognostic value. For that, they would need at least Cox or logistic models adjusted for standard clinical and molecular predictors, with effect sizes and confidence intervals, not only p-values. The IncuCyte statistics are a separate concern: a two-way ANOVA on longitudinal confluence measurements is not adequate unless the repeated structure is handled correctly or each well is reduced to an independent summary metric such as AUC, slope, or endpoint confluence. The statistical quality is acceptable for an exploratory mechanistic/biomarker manuscript, but it is not strong enough to support the more translational claims about prognosis, diagnostic utility, or independent predictive value. The methods describe the use of Kruskal-Wallis/Fisher tests for clinical associations, centroid prediction, sPLS with cross-validation, and two-way ANOVA for IncuCyte, which is precisely where the statistical limitations arise.

Novelty:
The focus on intratumoral A4 and the attempt to connect tumor steroid state with transcriptional phenotype and outcome is genuinely novel. The AR/androgen biology itself is not new, but the clinical framing around A4-associated tumor states is original.

Medical impact:
The work has clear potential relevance because it links A4-associated transcriptional states to proliferation, subtype, and survival, but the clinical utility is not yet demonstrated. The 90-gene signature is interesting, but it is not yet independently validated as prognostic, diagnostic, or treatment-predictive beyond established classifiers.

Referee #1 (Remarks for Author):

The revised manuscript is considerably improved, and I would like to thank the authors for the substantial effort made in

addressing the previous concerns. The clarification that the A4-associated groups represent a transcriptional gradient rather than discrete molecular subtypes, the additional PAM50/proliferation analyses, the estrone/aromatase comparisons, the more cautious framing of the snRNA-seq data, and the revised title all strengthen the manuscript. However, several points still require tightening before the conclusions are fully aligned with the data.

The most important issue at this point is the AR ChIP-seq section, where the interpretation still goes beyond what the experiment can support. As presented, these data show differential AR ChIP-seq signal in a very small number of A4-high and A4-low tumors, together with enrichment of MEF-family motifs among gained regions and DLX-family motifs among lost regions. This is interesting, but it should not be interpreted as evidence that AR is recruited to MEF2 target genes, that MEF2 activity is altered, or that MEF2 represents a functional downstream mechanism of A4 in breast cancer. Motif enrichment identifies sequence features associated with differential peaks; it does not establish transcription-factor activity, dependency, or causal involvement in the A4-associated phenotype. This section should therefore be rewritten as exploratory and descriptive, and the authors should provide sufficient ChIP-seq quality and analysis information to allow the reader to assess the robustness of the result, including peak-level summaries, replicate consistency, enrichment metrics, control handling, normalization, and multiple-testing correction for differential binding. It would also be useful to indicate whether the differential AR peaks overlap known AR-binding sites, A4-correlated genes, or the 90-gene signature, as this would directly test whether the ChIP-seq data are connected to the main transcriptional claims. I would also ask the authors to report the genomic distribution of these peaks, since AR binding is often predominantly distal/enhancer-associated, and this would help interpret whether the identified sites are consistent with expected AR regulatory architecture. Relatedly, the FKBP5 ChIP-qPCR experiment supports that A4 can recruit AR and selected coactivators to a canonical AR-responsive locus in vitro, but it should not be used as evidence that the clinical A4-associated signature is AR-driven without more direct integration; the apparent inconsistency between ZR-75-1 and T47D in the description of this experiment should also be corrected.

I also remain concerned that the functional growth assays are statistically under-described. The added T47D IncuCyte data are useful, but the ZR-75-1 time-course is visually less straightforward, with a non-monotonic growth pattern, substantial variability, and a late increase in the vehicle condition that should be explained. The authors report a strong p-value, but it is unclear whether this reflects a treatment main effect, a treatment-by-time interaction, an endpoint comparison, or an analysis of all time points without accounting for repeated measurements from the same wells. Since IncuCyte measurements are longitudinal, the authors should clarify the statistical unit, the number of biological and technical replicates, and whether the analysis was based on repeated-measures or mixed-effects modelling, or on independent per-well summaries such as AUC, endpoint confluence, or growth rate. Without this clarification, the statement that A4 dose-dependently inhibits growth in both cell lines remains somewhat stronger than the quantification supports.

Beyond this, the hormone-measurement methods should be clarified by reporting LLOD as well as LLOQ and by explaining how matrix effects and tissue-processing variability were handled, since these measurements are central to the manuscript.

Moreover, if the PAM50 proliferation analysis is used to justify retaining Ki67/MKI67 as the main proliferation readout, this comparison should be included in the manuscript or EV material rather than only in the response; otherwise, the statement should be made more restrained. Similarly, the PAM50 analysis is helpful, but it should be interpreted with care. Showing that A4 clusters can stratify outcome within PAM50-defined groups, particularly Luminal A, is not the same as demonstrating independent prognostic value. If the authors wish to retain claims that the 90-gene signature captures an additional prognostic dimension beyond established clinical and molecular predictors, this should be supported by an appropriate adjusted analysis, for example including subtype, proliferation, and relevant clinical variables. Otherwise, the signature should be described more conservatively as an A4-associated transcriptional classifier that may have prognostic relevance but still requires independent validation.

More generally, I would ask the authors to revisit the strength of the language throughout the manuscript. Several statements still imply that A4 has been demonstrated to be growth inhibitory in clinical breast cancer, that snRNA-seq "confirms" or provides "conclusive evidence" for this role, and that the 90-gene signature is already a robust clinical prognostic or diagnostic tool. In my view, the data support a more cautious conclusion: intratumoral A4 levels are associated with a transcriptional state linked to lower proliferation, more luminal features, and better outcome, with supportive in vitro evidence that A4 can inhibit growth of ER-positive/AR-positive breast cancer cell lines. They do not yet establish that A4 causally drives the clinical transcriptional state, prevents ER-negative/TNBC-like phenotypes, or can be used as a validated prognostic or diagnostic biomarker.

These revisions do not require reframing the entire manuscript, but they are important to ensure that the mechanistic and clinical conclusions remain proportionate to the evidence presented.

Reviewer: We thank the reviewer for the favorable opinion that this work is strong, novel and is a potentially important contribution to the field. We are grateful to the reviewer for a critical review (including the responses to other two reviewers) and providing several suggestions that significantly improved the quality and readability of the manuscript. We also had the manuscript reviewed by multiple individuals to ensure that the results are not depicted strongly. We hope we have responded appropriately to the reviewer's satisfaction.

1. *Even the revised framing remains somewhat stronger than warranted, because the manuscript still tends to present the intratumoral A4/transcriptome approach as defining the clinical role of androgens, whereas the data more conservatively support an association between A4-linked transcriptional states and tumour phenotype*.

   Response: We reviewed the entire manuscript and removed any reference to A4 as a tumor suppressor or one that defines the clinical role of androgens. We have now rephrased the manuscript to suggest that the data supports an association between A4-linked transcriptional states and tumor phenotype. We also had the manuscript reviewed by colleagues to ensure absence of over-interpretation.

2. *I also think Reviewer 3's concerns about intratumoral hormone measurement were not fully addressed: the authors clarify sample inclusion, but still do not sufficiently explain how tissue heterogeneity, matrix effects, pre-analytical variability, and robustness of A4 measurements across processing conditions were handled*.

   Response: We thank the reviewer for raising this important point regarding analytical validation of the steroid hormone measurements. We agree that since these measurements are central to several of the manuscript's conclusions, the methods section should provide greater detail on assay performance and quality control. The following details are now added to the manuscript method's section.

   > "*Intratumoral hormone measurement*
   >
   > Intratumoral hormone levels were quantified based on previously described and validated methods (Bhardwaj *et al*, 2023; Han *et al*, 2021). Gas chromatography-mass spectrometry (GC-MS)-based steroid profiling was performed using a Shimadzu GC 2010 Plus gas chromatograph interfaced with a triple-quadrupole Shimadzu GCMS-TQ8050 (Shimadzu Co., Kyoto, Japan). In brief, 5 mg of BC specimens were cut and added to 1 mL 0.2 M phosphate buffer (pH 7.2) and 20 µL of a mixture of internal standards (d8-17a-hydroxyprogesterone, 1 µg/mL; d6-DHEA and d9-progesterone, 0.5 µg/mL; d3-testosterone and d4-cortisol, 0.25 µg/mL; d4-17β-estradiol, 0.2 µg/mL). After pulverizing the tissues twice with TissueLyser II (Qiagen; Hilden, Germany) for 10 min at 25 Hz with three zirconia beads (3.0 mm I.D., Toray Industries, Tokyo, Japan), the samples were centrifuged for 5 min at 12,000 rpm, and the supernatant collected. The samples were then

loaded on a preconditioned Oasis HLB SPE cartridge. The cartridge was washed twice with 1 mL of water and eluted with 2 mL of methanol and 2 mL of 90% methanol. The combined eluates were evaporated under nitrogen at 40°C. Then, 1 mL of 0.2 M acetate buffer (pH 5.2) was added for additional extraction. After two extractions with 2.5 mL of methyl tert-butyl ether, the organic solvent was collected, evaporated under nitrogen at 35°C, and further dried in an amber vacuum desiccator with P2O5/KOH for at least 30 min. Finally, the dried residues were derivatized in 40 μL of MSTFA/NH4I/DTE (500:4:2, v/w/w) for 20 min at 60°C, and 2 μL of the resulting mixture was analyzed with GC-MS in the collision-induced dissociation mode.

Method validation and quantification limits. Method validation was performed according to the US Food and Drug Administration 2018 guideline for bioanalytical method validation (validation [US Food and Drug Administration. (2018) *Guidance for Industry: Bioanalytical method validation*, No. FDA-2013-D-1020, 1–41]). The following validation parameters were previously established using this assay (Han *et al.*, 2021) and were confirmed to be applicable to the current cohort by inclusion of quality-control samples at defined concentrations in each analytical run. The overall precision (%CV) and accuracy (%bias) across the analytical range were 0.8–19.9% and 69.9–111.1%, respectively. Per-analyte lower limits of quantification (LLOQ), defined as the lowest concentration quantifiable with acceptable accuracy was 0.1 ng/ml (Han et al) for all the analytes. Per-analyte lower limits of detection (LLOD), defined as the lowest concentration producing a signal-to-noise ratio of approximately 3, was 0.028 ng/ml for the analytes. As noted in the Results, DHT levels in a majority of specimens fell below the LLOQ and were therefore excluded from downstream analyses.

Matrix effects. Matrix effects were controlled using two complementary strategies. First, deuterated internal standards specific to each steroid class (listed above) were spiked into each tissue sample prior to homogenization and extraction; because internal standards are subject to the same ion suppression/enhancement as the corresponding native analyte, normalizing analyte signal to internal standard signal corrects for sample-to-sample differences in ionization efficiency. Second, calibration curves were prepared in matrix-matched surrogate tissue (rather than solvent-only standards) so that the calibration itself reflects the same matrix background present in patient specimens; each analyte was quantified against its corresponding isotope-labeled internal standard using these matrix-matched curves.

Tissue-processing variability. Several steps were taken to minimize and account for pre-analytical and processing variability across specimens. Tissue specimens were collected in the surgical suite, trimmed of adjacent fatty tissue, and snap-frozen within 30 minutes of excision to limit degradation prior to freezing. Homogenization was performed using a fixed protocol (TissueLyser II, 25 Hz, 10 min, two cycles) identical across all specimens to minimize inter-sample variability in extraction efficiency due to inconsistent tissue disruption. To account for differences in specimen size and cellular composition, hormone concentrations were normalized to tissue wet weight (ng/g tissue) rather than reported as absolute quantities. Internal standards, spiked prior to extraction, additionally correct for

sample-to-sample variability in extraction and derivatization efficiency downstream of tissue processing. Steroid concentrations were log-transformed and standardized to z-scores prior to downstream statistical analyses to reduce the influence of any residual technical variability on cluster assignment."

3. *Finally, the PAM50 analysis addresses subtype composition descriptively, but it does not establish that the A4 signature has independent prognostic value beyond subtype, proliferation, and standard clinical variables*.

   Response: We thank the reviewer for this important comment. We agree that established classifiers such as PAM50 and proliferation-based signatures capture important prognostic information in breast cancer. However, the primary goal of the present study was not to develop a competing prognostic assay. Rather, our objective was to determine whether intratumoral androstenedione (A4) levels, which are difficult to measure directly in routine clinical settings, could be inferred from tumor gene expression profiles and whether this androgen-associated biology is related to tumor characteristics and clinical outcome. Accordingly, the 90-gene signature should primarily be interpreted as a surrogate measure of intratumoral A4 activity rather than as a replacement for existing prognostic assays. Because intratumoral A4 is strongly associated with tumor differentiation and proliferation, it is not unexpected that the A4-associated signature also correlates with established prognostic features.

   To better understand the relationship between A4-associated biology and existing breast cancer classifiers, we performed additional analyses incorporating PAM50 intrinsic subtypes and PAM50-based proliferation scores. These analyses demonstrated that A4-associated groups are related to, but not equivalent to, intrinsic subtype classification. Importantly, A4-associated groups further stratified outcomes within Luminal A tumors, suggesting that androgen-associated biology captures clinically meaningful variation beyond conventional subtype assignment.

   In response to the reviewer's suggestion, we additionally performed multivariable Cox proportional hazards analyses adjusted for PAM50 subtype, age, tumor stage, and MKI67 expression (Response Table 3-1; Model 1). Because our experimental findings and prior studies suggest that steroid signaling exerts subtype-specific effects in breast cancer, we further incorporated an interaction term between A4-associated cluster and PAM50 subtype (Response Table 3-1; Model 2). Comparison of models with and without the interaction term using a likelihood ratio test demonstrated a significant interaction between A4-associated cluster and PAM50 subtype ($P = 0.017$; Response Table 3-2), indicating that the prognostic effect of A4-associated biology differs across intrinsic breast cancer subtypes.

   Given this subtype-specific effect, we next focused on Luminal A tumors, where androgen signaling is expected to play the most biologically relevant role. Within the Luminal A subgroup, A4-associated cluster remained significantly associated with survival after

adjustment for age, tumor stage, and MKI67 expression (Response Table 3-3). These findings suggest that A4-associated biology captures clinically relevant variation beyond conventional clinical variables and tumor proliferative status within Luminal A breast cancer.

Overall, these results suggest that A4-associated biology represents an additional dimension of tumor heterogeneity that may complement existing prognostic measures. Nevertheless, we agree with the reviewer that further validation in larger, clinically annotated cohorts will be necessary to determine whether A4-associated signatures provide clinically actionable prognostic information beyond existing assays.

These additional analyses have been incorporated into the revised manuscript (Results section and EV Tables). We have also revised the manuscript to clarify that the prognostic significance of A4-associated biology is subtype-dependent rather than universal across all breast cancers, and that the clinical analyses are intended to characterize the biological and clinical relevance of intratumoral A4-associated states rather than to establish the 90-gene signature as a standalone prognostic assay.

The following section has been incorporated in the results section:

> "To determine whether the prognostic effect of A4 clusters differed across intrinsic breast cancer subtypes, we performed multivariable Cox proportional hazards analyses. In the overall cohort, the prognostic association of A4 clusters was attenuated after adjustment for PAM50 subtype, age, tumor stage, and MKI67 expression (**Table EV2**; Model 1). However, given prior evidence that steroid signaling exerts subtype-specific effects in BC, we next evaluated an interaction between A4 clusters and PAM50 subtype (**Table EV2**; Model 2). Inclusion of the interaction term significantly improved model fit compared with the model containing only main effects (likelihood ratio test, $\chi^2 = 16.99$, df = 7, p = 0.017; **Table EV3**). These findings indicate that the association between A4-associated gene expression and survival is subtype-dependent.
>
> Given the significant interaction between A4 cluster and PAM50 subtype, we next evaluated the prognostic significance of A4 clusters specifically within Luminal A tumors. In multivariable Cox proportional hazards analyses adjusting for age, tumor stage, and MKI67 expression, A4 cluster remained significantly associated with overall survival (**Table EV4**). Compared with A4-low tumors, patients with A4-high tumors exhibited significantly improved survival (HR = 0.13, 95% CI: 0.02–0.77, $P = 0.025$), whereas A4-intermediate tumors showed a similar trend that did not reach statistical significance (HR = 0.25, 95% CI: 0.06–1.03, $P = 0.055$). These findings indicate that androgen-associated gene expression could capture biologically meaningful variation beyond conventional subtype classification and may contribute to additional clinically relevant information, particularly within Luminal A tumors."

	Model 1		Model 2	
**Variable**	**HR (95% CI)**	***P* value**	**HR (95% CI)**	***P* value**
**A4 cluster**				
A4-low (reference)				
A4-inter	1.175 (0.566-2.442)	0.6649	0.125 (0.035-0.448)	0.0014**
A4-high	0.593 (0.208-1.686)	0.3268	0.05 (0.009-0.29)	<0.001***
**PAM50 subtype**				
Luminal A (reference)				
Luminal B	1.557 (0.605-4.003)	0.3582	0.09 (0.013-0.612)	0.0138*
HER2-enriched	4.166 (1.629-10.655)	0.0029**	1.204 (0.11-13.137)	0.8791
Basal-like	3.848 (1.384-10.701)	0.0098**	0.28 (0.053-1.488)	0.1351
Normal-like	1.031 (0.283-3.756)	0.9626	2.636 (0.437-15.898)	0.2904
**Age**	1.053 (1.031-1.075)	<0.001***	1.052 (1.029-1.074)	<0.001***
**Tumor stage**				
Stage I (reference)				
Stage II	1.184 (0.497-2.821)	0.7027	1.386 (0.571-3.367)	0.4705
Stage III	4.459 (1.947-10.21)	<0.001***	5.581 (2.317-13.446)	<0.001***
**MKI67**	0.874 (0.66-1.157)	0.3457	0.958 (0.723-1.269)	0.7645
**Interaction**				
A4-inter:Luminal B			21.431 (3.027-151.754)	0.0021**
A4-high:Luminal B			-	-
A4-inter:HER2-enriched			3.11 (0.278-34.751)	0.3569
A4-high:HER2-enriched			7.461 (0.378-147.162)	0.1865
A4-inter:Basal-like			17.957 (3.422-94.23)	<0.001***
A4-high:Basal-like			12.864 (0.8-206.91)	0.0715
A4-inter:Normal-like			-	-
A4-high:Normal-like			-	-

Response Table 3-1. Multivariable Cox proportional hazards models.

Model 1 (main effects): A4 cluster + PAM50 subtype + age + tumor stage + MKI67

Model 2 (with interaction): A4 cluster × PAM50 subtype + age + tumor stage + MKI67

Hazard ratios (HRs) and 95% confidence intervals (CIs) were estimated using multivariable Cox proportional hazards models for overall survival. Model 1 included A4-associated cluster, PAM50 intrinsic

subtype, age, tumor stage, and MKI67 expression as main effects. Model 2 additionally included interaction terms between A4-associated cluster and PAM50 intrinsic subtype. The reference categories were A4-low cluster, Luminal A subtype, and Stage I disease. Age and MKI67 were modeled as continuous variables.

Model	Log-likelihood	df	χ^2	P value
Model 1 (main effects)	-325.79	-	-	-
Model 2 (with interaction)	-317.29	7	16.99	0.017 *

Response Table 3-2. Likelihood ratio test for interaction (Model 1 vs Model 2)

The significance of the interaction between A4-associated cluster and PAM50 subtype was assessed by comparing nested Cox proportional hazards models using a likelihood ratio test (LRT). A significant interaction indicates that the potential prognostic effect of A4-associated clusters differs according to intrinsic breast cancer subtype.

Variable	HR (95% CI)	*P* value
**A4 cluster**		
A4-low		
A4-inter	0.247 (0.059-1.029)	0.0547
A4-high	0.128 (0.021-0.769)	0.0247*
**Age**	1.149 (1.078-1.224)	<0.001***
**Tumor stage**		
Stage I		
Stage II	2.59 (0.683-9.813)	0.1615
Stage III	3.974 (0.844-18.71)	0.0809
**MKI67**	1.206 (0.659-2.206)	0.5431

Response Table 3-3. Multivariable Cox proportional hazards model (Luminal A only)

Hazard ratios (HRs) and 95% confidence intervals (CIs) were estimated using a multivariable Cox proportional hazards model restricted to Luminal A tumors. The model included A4-associated cluster, age, tumor stage, and MKI67 expression. Reference categories were A4-low cluster and Stage I disease. Age and MKI67 expression were modeled as continuous variables. Overall survival was used as the outcome measure.

4. *Their sPLS/90-gene analysis is useful as an internal feature-reduction exercise, but it is not a proper clinical prediction validation: the final model is fit in a small discovery set, the LOOCV is mainly used to argue gene-set stability, and the TCGA analysis validates inferred cluster behavior rather than measured intratumoral A4.*

   Response: We thank the reviewer for this important comment. We agree that formal external validation of the 90-gene predictor would ideally require an independent cohort with matched transcriptomic profiles and directly measured intratumoral A4 levels. However, to our knowledge, publicly available datasets containing both gene expression data and matched intratumoral A4 measurements are currently not available. Consequently, independent external validation of A4 prediction was not feasible in the present study.

   Importantly, the primary objective of the 90-gene classifier was not to develop or clinically validate a prognostic or diagnostic assay. Rather, the reduced gene set was developed to determine whether the major biological and clinical characteristics associated with intratumoral A4 could be reproducibly captured using a substantially smaller set of genes. Similarly, the TCGA analyses were not intended as external validation of measured intratumoral A4 prediction, but rather to evaluate whether the A4-associated states identified in our discovery cohort exhibit reproducible biological and clinical characteristics in an independent breast cancer cohort.

   We therefore agree that the TCGA analyses validate the biological and clinical characteristics associated with A4 groups rather than directly validating measured intratumoral A4 levels. We have revised the manuscript to clarify this distinction and to avoid overstatement regarding the clinical implementation of the predictor:

   Section title:

   “Prediction and validation with the 90-gene classifier” has been changed to “A reduced 90-gene classifier reproducibly captures A4 tumor states and their clinical relevance”

   In Results:

   “Using the reduced gene set, we developed a 90-gene classifier based on a modified centroid prediction method and evaluated whether the major biological and clinical characteristics identified using the full 1,388 gene signature could be reproducibly captured using a substantially smaller gene set (Methods).”

5. *The IncuCyte statistics are a separate concern: a two-way ANOVA on longitudinal confluence measurements is not adequate unless the repeated structure is handled correctly or each well is reduced to an independent summary metric such as AUC, slope, or endpoint confluence.*

Response: We have reanalyzed the incucyte using area under the curve (AUC) and found that the A4 treated samples' growth is statistically significant compared to the vehicle-treated cells. This information is now added to the methods section and in the legends.

6. *The methods describe the use of Kruskal-Wallis/Fisher tests for clinical associations, centroid prediction, sPLS with cross-validation, and two-way ANOVA for IncuCyte, which is precisely where the statistical limitations arise*.

   Response: We thank the reviewer for highlighting these important considerations. The statistical methods used in this study, including Kruskal-Wallis tests, Fisher's exact tests, nearest-centroid classification, sparse partial least squares (sPLS), and two-way ANOVA, were selected to address specific analytical objectives and represent widely used approaches for analyzing clinical associations, classification, high-dimensional transcriptomic data, and functional experiments.

   We agree that unadjusted association analyses alone do not establish independent prognostic value. The primary purpose of these analyses was to characterize the biological and clinical relationships between intratumoral A4 states and clinicopathological variables, rather than to develop a clinically validated prognostic model. Similarly, the sPLS analyses should be viewed as proof-of-concept demonstrating the feasibility of inferring intratumoral A4-associated biology from gene expression profiles rather than definitive clinical validation.

   To address the reviewer's concern, we have now performed additional multivariable Cox proportional hazards analyses incorporating established prognostic variables, including PAM50 subtype, age, tumor stage, and MKI67 expression (see response to Comment #3 for details). These analyses demonstrated a significant interaction between A4 clusters and PAM50 subtype, indicating that the prognostic effect of A4 biology is subtype-dependent. Furthermore, within Luminal A tumors, A4 clusters remained significantly associated with survival after adjustment for age, tumor stage, and MKI67 expression. These additional analyses have been incorporated into the revised manuscript, and the text has been revised accordingly as we described in response to Comment #3.

   We also have revised the manuscript to clarify the scope of the statistical analyses and to adopt a more conservative interpretation of the prognostic findings. (See response in Comment #12)

7. *The most important issue at this point is the AR ChIP-seq section, where the interpretation still goes beyond what the experiment can support. As presented, these data show differential AR ChIP-seq signal in a very small number of A4-high and A4-low tumors, together with enrichment of MEF-family motifs among gained regions and DLX-family motifs among lost regions. This is interesting, but it should not be interpreted as evidence*

that AR is recruited to MEF2 target genes, that MEF2 activity is altered, or that MEF2 represents a functional downstream mechanism of A4 in breast cancer. Motif enrichment identifies sequence features associated with differential peaks; it does not establish transcription-factor activity, dependency, or causal involvement in the A4-associated phenotype. This section should therefore be rewritten as exploratory and descriptive, and the authors should provide sufficient ChIP-seq quality and analysis information to allow the reader to assess the robustness of the result, including peak-level summaries, replicate consistency, enrichment metrics, control handling, normalization, and multiple-testing correction for differential binding. It would also be useful to indicate whether the differential AR peaks overlap known AR-binding sites, A4-correlated genes, or the 90-gene signature, as this would directly test whether the ChIP-seq data are connected to the main transcriptional claims. I would also ask the authors to report the genomic distribution of these peaks, since AR binding is often predominantly distal/enhancer-associated, and this would help interpret whether the identified sites are consistent with expected AR regulatory architecture.

Response: We thank the reviewer for this comment. We have revised this section providing all the information requested by the reviewer. The new results section reads as follows:

This figure and text referred to data that was removed from the final version of the manuscript.

8. *Relatedly, the FKBP5 ChIP-qPCR experiment supports that A4 can recruit AR and selected coactivators to a canonical AR-responsive locus in vitro, but it should not be used as evidence that the clinical A4-associated signature is AR-driven without more direct integration; the apparent inconsistency between ZR-75-1 and T47D in the description of this experiment should also be corrected*.

   Response: We thank the reviewer for this comment, especially for pointing out to the inconsistency between the figure and the legend. We have now corrected the discrepancy between ZR-75-1 and T47D cells in the legend. We have revised this section to ensure that the interpretation is focused on the DNA binding and not extrapolating to gene expression changes. The revised section reads:

This text referred to data that was removed from the final version of the manuscript.

9. *I also remain concerned that the functional growth assays are statistically under-described. The added T47D IncuCyte data are useful, but the ZR-75-1 time-course is visually less straightforward, with a non-monotonic growth pattern, substantial variability, and a late increase in the vehicle condition that should be explained. The authors report a strong p-value, but it is unclear whether this reflects a treatment main effect, a treatment-by-time interaction, an endpoint comparison, or an analysis of all time points without accounting for repeated measurements from the same wells. Since IncuCyte measurements are longitudinal, the authors should clarify the statistical unit, the number of biological and technical replicates, and whether the analysis was based on repeated-measures or mixed-effects modelling, or on independent per-well summaries such as AUC, endpoint confluence, or growth rate. Without this clarification, the statement that A4 dose-dependently inhibits growth in both cell lines remains somewhat stronger than the quantification supports*.

   Response: We thank the reviewer for requesting additional clarification on incucyte data analysis. We have provided additional details regarding incucyte data analysis. We have also added a new incucyte experiment that provides better inhibition by A4. The results section reads;

   > "A4 dose-dependently inhibited the growth of ZR-75-1 and T47D cells over a 14-day period, as measured by IncuCyte confluence (**Fig. 6C**). Using a repeated-measures two-way ANOVA that accounts for the longitudinal, same-well structure of the data, we observed a significant main effect of treatment × time interaction ($p=0.00006$). This result was corroborated by an independent, replicate-level AUC analysis, confirming that the effect was not dependent on the specific repeated-measures modeling approach.
   >
   > Since A4 is not a cytotoxic agent, the magnitude of growth inhibition observed (~40% relative to vehicle) is consistent with a cytostatic rather than cytotoxic mechanism, in line with effect sizes reported for other cytostatic compounds in comparable long-term proliferation assays.
   >
   > The ZR-75-1 vehicle-treated growth curve showed a non-monotonic pattern, including a late increase in confluence (occurring after the day-9 medium change, which we attribute to renewed nutrient availability following a confluence-associated growth plateau; this pattern was reproducible across independent experiments and was specific to the vehicle condition, likely reflecting medium exhaustion between the scheduled every-third-day medium changes in this cell line). This pattern did not alter the outcome of either the repeated-measures model or the AUC-based comparison, both of which incorporate the full time-course rather than a single endpoint measurement.".

The new incucyte ZR-75-1 data is provided below for the reviewer's convenience. Since A4 is not a cytotoxic drugs, in vitro proliferation and growth assays provide only modest growth inhibition. The inhibition we observed is approximately 40%, which is in line with several cytostatic drugs.

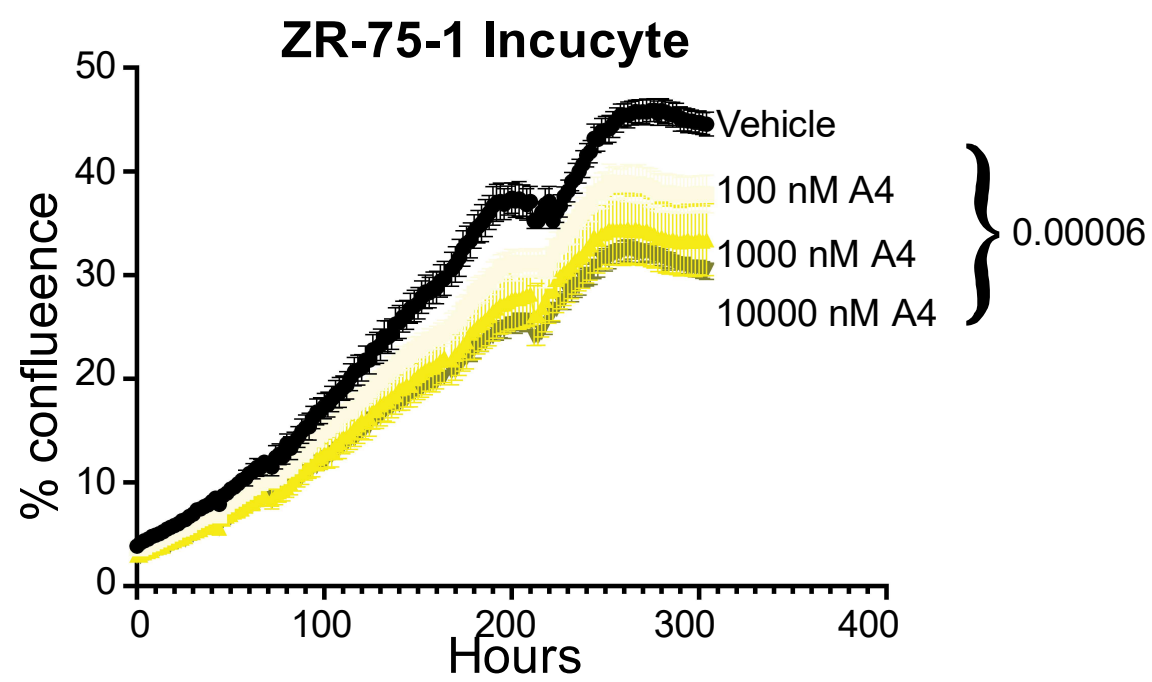

The methods section has also been changed. The new methods section is provided below:

> "Cells were plated in 96-well plates in growth medium at varying density, with n=4 replicate wells per condition within each experiment. Cells were treated with vehicle or A4 at the indicated concentrations. Plates were incubated in an IncuCyte live-cell imaging system and imaged every 4 hours for confluence. Growth medium containing the corresponding treatment was refreshed every third day for the duration of the assay. Each experiment was independently repeated at least two times (biological replicates). Well-level (technical replicate) confluence values were averaged within each biological replicate prior to statistical analysis.
>
> Longitudinal confluence data were analyzed using a repeated-measures two-way ANOVA (treatment × time, with biological replicate as the repeated unit), followed by post-hoc comparisons at each timepoint with correction for multiple comparisons. The p-value reported in the results corresponds to the treatment × time interaction. A secondary replicate-level analysis that does not depend on the repeated-measures correlation structure, area under the confluence curve (AUC) was calculated for each biological replicate using the trapezoidal method, and AUC values were compared between treatment and vehicle groups using Mann-Whitney U test. Statistical significance was set at $p<0.05$. Analyses were performed using GraphPard Prism."

10. *Beyond this, the hormone-measurement methods should be clarified by reporting LLOD as well as LLOQ and by explaining how matrix effects and tissue-processing variability were handled, since these measurements are central to the manuscript*?

Response: We thank the reviewer for raising this important point regarding analytical validation of the steroid hormone measurements. We agree that since these measurements are central to several of the manuscript's conclusions, the methods section should provide greater detail on assay performance and quality control. The following details are now added to the manuscript method's section.

"*Intratumoral hormone measurement*

Intratumoral hormone levels were quantified based on previously described and validated methods (Bhardwaj *et al*, 2023; Han *et al*, 2021). Gas chromatography-mass spectrometry (GC-MS)-based steroid profiling was performed using a Shimadzu GC 2010 Plus gas chromatograph interfaced with a triple-quadrupole Shimadzu GCMS-TQ8050 (Shimadzu Co., Kyoto, Japan). In brief, 5 mg of BC specimens were cut and added to 1 mL 0.2 M phosphate buffer (pH 7.2) and 20 µL of a mixture of internal standards (d8-17a-hydroxyprogesterone, 1 µg/mL; d6-DHEA and d9-progesterone, 0.5 µg/mL; d3-testosterone and d4-cortisol, 0.25 µg/mL; d4-17β-estradiol, 0.2 µg/mL). After pulverizing the tissues twice with TissueLyser II (Qiagen; Hilden, Germany) for 10 min at 25 Hz with three zirconia beads (3.0 mm I.D., Toray Industries, Tokyo, Japan), the samples were centrifuged for 5 min at 12,000 rpm, and the supernatant collected. The samples were then loaded on a preconditioned Oasis HLB SPE cartridge. The cartridge was washed twice with 1 mL of water and eluted with 2 mL of methanol and 2 mL of 90% methanol. The combined eluates were evaporated under nitrogen at 40°C. Then, 1 mL of 0.2 M acetate buffer (pH 5.2) was added for additional extraction. After two extractions with 2.5 mL of methyl tert-butyl ether, the organic solvent was collected, evaporated under nitrogen at 35°C, and further dried in an amber vacuum desiccator with P2O5/KOH for at least 30 min. Finally, the dried residues were derivatized in 40 µL of MSTFA/NH4I/DTE (500:4:2, v/w/w) for 20 min at 60°C, and 2 µL of the resulting mixture was analyzed with GC-MS in the collision-induced dissociation mode.

Method validation and quantification limits. Method validation was performed according to the US Food and Drug Administration 2018 guideline for bioanalytical method validation (validation [US Food and Drug Administration. (2018) *Guidance for Industry: Bioanalytical method validation*, No. FDA-2013-D-1020, 1–41]). The following validation parameters were previously established using this assay (Han *et al.*, 2021) and were confirmed to be applicable to the current cohort by inclusion of quality-control samples at defined concentrations in each analytical run. The overall precision (%CV) and accuracy (%bias) across the analytical range were 0.8–19.9% and 69.9–111.1%, respectively. Per-analyte lower limits of quantification (LLOQ), defined as the lowest concentration quantifiable with acceptable accuracy was 0.1 ng/ml (Han et al) for all the analytes. Per-analyte lower limits of detection (LLOD), defined as the lowest concentration producing a signal-to-noise ratio of approximately 3, was 0.028 ng/ml for the analytes. As noted in the Results, DHT levels in a majority of specimens fell below the LLOQ and were therefore excluded from downstream analyses.

Matrix effects. Matrix effects were controlled using two complementary strategies. First, deuterated internal standards specific to each steroid class (listed above) were spiked into each tissue sample prior to homogenization and extraction; because internal standards are subject to the same ion suppression/enhancement as the corresponding native analyte, normalizing analyte signal to internal standard signal corrects for sample-to-sample differences in ionization efficiency. Second, calibration curves were prepared in matrix-matched surrogate tissue (rather than solvent-only standards) so that the calibration itself reflects the same matrix background present in patient specimens; each analyte was quantified against its corresponding isotope-labeled internal standard using these matrix-matched curves.

Tissue-processing variability. Several steps were taken to minimize and account for pre-analytical and processing variability across specimens. Tissue specimens were collected in the surgical suite, trimmed of adjacent fatty tissue, and snap-frozen within 30 minutes of excision to limit degradation prior to freezing. Homogenization was performed using a fixed protocol (TissueLyser II, 25 Hz, 10 min, two cycles) identical across all specimens to minimize inter-sample variability in extraction efficiency due to inconsistent tissue disruption. To account for differences in specimen size and cellular composition, hormone concentrations were normalized to tissue wet weight (ng/g tissue) rather than reported as absolute quantities. Internal standards, spiked prior to extraction, additionally correct for sample-to-sample variability in extraction and derivatization efficiency downstream of tissue processing. Steroid concentrations were log-transformed and standardized to z-scores prior to downstream statistical analyses to reduce the influence of any residual technical variability on cluster assignment."

11. *Moreover, if the PAM50 proliferation analysis is used to justify retaining Ki67/MKI67 as the main proliferation readout, this comparison should be included in the manuscript or EV material rather than only in the response; otherwise, the statement should be made more restrained. Similarly, the PAM50 analysis is helpful, but it should be interpreted with care. Showing that A4 clusters can stratify outcome within PAM50-defined groups, particularly Luminal A, is not the same as demonstrating independent prognostic value. If the authors wish to retain claims that the 90-gene signature captures an additional prognostic dimension beyond established clinical and molecular predictors, this should be supported by an appropriate adjusted analysis, for example including subtype, proliferation, and relevant clinical variables. Otherwise, the signature should be described more conservatively as an A4-associated transcriptional classifier that may have prognostic relevance but still requires independent validation*.

Response: We thank the reviewer for this thoughtful comment and agree that claims regarding prognostic value should be interpreted with caution. As suggested, we have now included the comparison between MKI67 and the PAM50 proliferation score in the revised manuscript. Consistent with our original analyses, the PAM50 proliferation score was highly correlated with Ki67 and MKI67 expression and showed similar associations with A4-associated clusters, supporting the use of Ki67/MKI67 as representative proliferation

markers throughout the manuscript. We have added the following text to the Results section:

> "Since TCGA dataset lacks ki67 measurements, *MKI67* expression was used as a surrogate proliferation marker. In our clinical cohort, *MKI67* expression was highly correlated with both ki67 and the PAM50-based proliferation score, supporting its use as representative RNA-based proliferation readout."

We also agree that stratification of outcomes within PAM50-defined groups alone does not establish independent prognostic value. As described in our response above (Comment #3), we have now performed additional multivariable Cox proportional hazards analyses incorporating established prognostic variables, including PAM50 subtype, age, tumor stage, and MKI67 expression. These analyses demonstrated that the prognostic association of A4 clusters is subtype-dependent and that, within Luminal A tumors, A4 clusters remained significantly associated with survival after adjustment for age, tumor stage, and MKI67 expression (Response Tables 3-1, 3-2, 3-3).

Importantly, the primary objective of this study was not to develop a prognostic classifier competing with existing breast cancer signatures, but rather to characterize intratumoral A4-associated biology and evaluate whether these biological states are associated with clinically relevant phenotypes. Accordingly, we have revised the manuscript throughout to adopt a more conservative interpretation of the prognostic findings and now describe the 90-gene signature primarily as an A4-associated transcriptional classifier with potential prognostic relevance that warrants further independent validation.

Specifically, the Abstract, Results, and Discussion sections have been revised to avoid overstating clinical utility and to emphasize that additional validation in larger, independent cohorts will be required before clinical application can be considered.

The following section has been added to the Abstract:

> "…, a 90-gene A4-dependent signature that predicted tumor aggressiveness and survival outcomes …" → "…, we developed a 90-gene A4-associated transcriptional classifier that captures intratumoral A4-associated biology and may have potential prognostic relevance."

The following section has been added to the Results section:

> These findings indicate that androgen-associated gene expression could capture additional prognostic dimension beyond conventional subtype classification. → "These findings indicate that androgen-associated gene expression could capture biologically meaningful variation beyond conventional subtype classification and

may contribute additional clinically relevant information, particularly within Luminal A tumors."

The following section has been added to the Discussion:

Hence, the list of genes in A4 cluster high will have high predictive value of grade, stage, and survival, and this could potentially eliminate some of the bias in immunohistochemical staining and pathological evaluation. → Hence, the list of the identified genes for separating the A4-low and A4-high groups will have high predictive value of grade, stage, and survival, and this could potentially eliminate some of the bias in immunohistochemical staining and pathological evaluation.

On this front, the 90 gene signature that highly correlates with intratumoral A4 has the potential to serve as a reliable predictive biomarker that can be used to predict future recurrence, survival, and other characteristics. → On this front, the 90 gene signature that highly correlates with intratumoral A4 has the potential to serve as a reliable predictive biomarker that can be used to predict future recurrence, survival, and other characteristics. However, additional validation in independent cohorts will be necessary before its clinical utility can be established.

12. *More generally, I would ask the authors to revisit the strength of the language throughout the manuscript. Several statements still imply that A4 has been demonstrated to be growth inhibitory in clinical breast cancer, that snRNA-seq "confirms" or provides "conclusive evidence" for this role, and that the 90-gene signature is already a robust clinical prognostic or diagnostic tool. In my view, the data support a more cautious conclusion: intratumoral A4 levels are associated with a transcriptional state linked to lower proliferation, more luminal features, and better outcome, with supportive in vitro evidence that A4 can inhibit growth of ER-positive/AR-positive breast cancer cell lines. They do not yet establish that A4 causally drives the clinical transcriptional state, prevents ER-negative/TNBC-like phenotypes, or can be used as a validated prognostic or diagnostic biomarker.*

Response: We thank the reviewer for this comment. We have now revised the entire manuscript to ensure that the interpretation does not suggest that A4 inhibits growth or A4 has caused the clinical phenotype. As suggested by the reviewer, we have now described the entire dataset as an association between A4-dependent transcriptome and lower proliferative state, luminal features, and better clinical outcome. We have also indicated in the in vitro section that A4 can inhibit growth of ER-positive/AR-positive breast cancer cell lines.

2nd Aug 2026

Dear Dr. Narayanan,

Thank you for submitting your revised manuscript to EMBO Molecular Medicine. I have now received the report from referee 1, who is overall satisfied with your revisions. I will therefore be pleased to accept your manuscript once the remaining issue has been addressed.

As outlined below, the referee remains concerned about the AR ChIP-seq data and therefore recommends removing Figure EV4D, together with all other ChIP-seq- and ChIP-qPCR-based analyses and the associated conclusions, including the differential binding, heatmap and motif analyses, as well as the proposed AR-MEF2 regulatory mechanism.

I appreciate your continued efforts in revising the manuscript and thank you for your patience throughout the review process. Given the referee's overall positive assessment, I anticipate being able to proceed with acceptance once these changes have been addressed.

I look forward to seeing a revised form of your manuscript.

With kind regards,

Lise Roth

Lise Roth, Ph.D
Senior Editor
EMBO Molecular Medicine

***** Reviewer's comments *****

Referee #1 (Comments on Novelty/Model System for Author):

As I mentioned previously, this is an interesting study and I do think it contributes to the ongoing debates in the field.

Referee #1 (Remarks for Author):

The authors have made a substantial effort to address the previous comments, and the manuscript has improved considerably. However, their clarification regarding the AR ChIP-seq does not resolve my concern; it has actually raised further concerns over the quality of the ChIP data. The identification of approximately 500k-800k AR peaks in each sample is not biologically credible for a sequence-specific transcription factor and is more than an order of magnitude greater than established AR cistromes (2k on low end up to 40k or so on higher end). At this density, an apparent AR-binding site would occur approximately every 4-6 kb across the genome, placing a peak close to essentially every gene and most regulatory elements. This would make the dataset almost non-discriminatory and would imply an implausibly pervasive pattern of AR occupancy. Although this does not mean that every nearby gene is genuinely regulated by AR, it does indicate that the peak set cannot reliably distinguish specific AR binding from background or generally accessible chromatin.

The fact that many peaks have an extremely low reported FDR does not resolve this problem, because statistical confidence assigned by the peak caller is not equivalent to biochemical specificity. Similarly, restricting the downstream analyses to the approximately 20% of peaks shared between samples does not validate those regions when they were selected from an initial peak set in which the overwhelming majority of calls are questionable. The authors state that a background control was used, but they do not explain what this control was, whether it was matched to each sample, or how it was incorporated into the analysis.

Given the magnitude of this problem, my concern extends to all conclusions based on the AR ChIP experiments, including the targeted ChIP-qPCR. A targeted ChIP-qPCR measurement cannot be considered independent validation of the ChIP-seq when

it relies on the same experimental principle and may be testing loci selected from the same questionable dataset. Enrichment at one or several preselected loci does not establish that the immunoprecipitated material represents specific AR occupancy genome-wide. I therefore recommend removing Figure EV4D and all other ChIP-seq- or ChIP-qPCR-based analyses and conclusions, including the differential-binding, heatmap and motif analyses and the proposed AR-MEF2 regulatory mechanism. I would not request that the authors repeat these experiments at this stage. The central clinical and transcriptomic observations remain interesting, and removing the ChIP-based component would prevent technically unreliable data from weakening the otherwise potentially valuable message of the manuscript.

The authors addressed the editorial issues.

3rd Sep 2026

Dear Dr. Narayanan,

Thank you for submitting your revised manuscript to EMBO Molecular Medicine, and please accept my apologies for the delay in getting back to you during this busy time of the year. I am pleased to say that I will be able to accept your manuscript once the following minor editorial concerns are addressed:

1/ Manuscript text:
- Please accept the previous changes and only indicate any new modifications to the text using track changes.
- Methods:
o Please provide the full statement that the studies conformed to the principles set out in the WMA Declaration of Helsinki and the Department of Health and Human Services Belmont Report.
o Please provide additional methodological details for the Western blot experiments, including antibody dilutions and sufficient information to allow the experiments to be reproduced.
o Please add COS-7 cells to the Reagent Table.
o Please provide information on sample-size determination, inclusion and exclusion criteria, randomization and blinding, as applicable to the different experiments.
o Please ensure that the number and nature of experimental replicates are clearly reported throughout the Methods and figure legends, distinguishing biological from technical replicates where applicable. Please also clarify the apparent discrepancy between the statement that all in vitro experiments were performed at least three times independently and the Incucyte experiments, which are described as having been independently repeated at least twice.
o Please check and correct the reported RNA-seq sample numbers: the Methods indicate 61 samples in the clinical cohort and 77 in the validation cohort (total n=138), whereas the Gene expression pre-processing section reports a total of n=139.
- Data Availability section: Please provide URLs for the deposited datasets and note that the datasets must be publicly available before acceptance of the manuscript. Please also check the Data Availability section and GEO accession numbers for accuracy. The text gives accession number GSE349224, while the corresponding link points to GSE329224, so one of these appears to be incorrect. Please remove the accession numbers from the Reagent Table.
- Please address the remaining queries from our data editors in the figure legends:
o Please provide exact p values for Figure 4.
o Please indicate the statistical tests used for data analysis in the legends of Figs 4J and EV4.
o Please note that information related to n is missing from the legends of Figs 6A, EV3, and EV4C.
o Please note that the error bars are not defined in the legend of Fig. EV4.

2/ Author checklist:
Please fill in the "Experimental study design and statistics" section, subsection "Blinding".

3/ Source Data:
Please provide the completed Source Data checklist.

4/ The paper explained:
Thank you for providing a nice summary. Please carefully check the text for typos and grammar.

5/ Synopsis:
I introduced minor edits to your text; please let me know if you agree or amend as you see fit:

Preclinical studies and circulating androgen levels suggest that androgens have distinct effects in breast cancer, inhibiting tumor growth in estrogen receptor (ER)-positive breast cancer while promoting growth in triple-negative breast cancer (TNBC).
• An intratumoral androstenedione (A4)-dependent signature correlates with favorable outcomes in patients with breast cancer.
• The A4-dependent signature inversely correlates with Ki67, tumor stage, and grade, and positively correlates with patient survival.
• The association of the signature with outcome is subtype-dependent, with a strong correlation in ER-positive breast cancer and little to no correlation in TNBC.
• The effects of A4 in breast cancer are mediated by its receptor, the androgen receptor (AR).
Unfortunately, the resolution of the visual abstract is too low. Could you please provide a higher-resolution image with dimensions of 550 × 300 pixels? Please note that the use of BioRender should be disclosed.

6/ Review Process File:
As part of the EMBO Publications transparent editorial process initiative (see our Editorial at http://embomolmed.embopress.org/content/2/9/329), EMBO Molecular Medicine will publish online a Review Process File (RPF) to accompany accepted manuscripts.
This file will be published in conjunction with your paper and will include the anonymous referee reports, your point-by-point

response, and all pertinent correspondence relating to the manuscript. Please let us know whether you agree with the publication of the RPF and whether you would like any figures to be removed from it prior to publication.
Please note that the Author Checklist will be published at the end of the RPF.

I look forward to receiving your revised manuscript.

Yours sincerely,

Lise Roth

Lise Roth, PhD
Senior Editor
EMBO Molecular Medicine

The authors addressed the remaining editorial issues.

10th Sep 2026

Dear Dr. Narayanan,

Thank you for submitting your revised files. I am pleased to inform you that your manuscript is accepted for publication and is now being sent to our publisher to be included in the next available issue of EMBO Molecular Medicine.

Your manuscript will be processed for publication by EMBO Press. It will be copy edited and you will receive page proofs prior to publication. Please note that you will be contacted by Springer Nature Author Services to complete licensing and payment information.

You may qualify for financial assistance for your publication charges - either via a Springer Nature fully open access agreement or an EMBO initiative. Check your eligibility: https://link.springer.com/journal/44321/how-to-publish-with-us

Should you be planning a Press Release on your article, please notify the production team via this form in order to coordinate publication and release dates: https://forms.cloud.microsoft/e/eH4A8JVP6g

If you have any questions, please do not hesitate to contact the Editorial Office. Thank you for your contribution to EMBO Molecular Medicine.

Yours sincerely,

Lise Roth

Lise Roth, Ph.D
Senior Editor
EMBO Molecular Medicine

>>> Please note that it is EMBO Molecular Medicine policy for the transcript of the editorial process (containing referee reports and your response letter) to be published as an online supplement to each paper. If you do NOT want this, you will need to inform the Editorial Office via email immediately. More information is available here: https://link.springer.com/partners/embo-press/editorial-policies#Peer%20review

EMBO Press Author Checklist

Corresponding Author Name: Ramesh Narayanan and Hyo Young Choi	
Journal Submitted to: EMBO Molecular Medicine	
Manuscript Number: EMM-2025-23231	

USEFUL LINKS FOR COMPLETING THIS FORM
The EMBO Journal - Author Guidelines
EMBO Reports - Author Guidelines
Molecular Systems Biology - Author Guidelines
EMBO Molecular Medicine - Author Guidelines

Reporting Checklist for Life Science Articles (updated January
This checklist is adapted from Materials Design Analysis Reporting (MDAR) Checklist for Authors. MDAR establishes a minimum set of requirements in transparent reporting in the life sciences (see Statement of Task: 10.31222/osf.io/9sm4x). Please follow the journal's guidelines in preparing your
Please note that a copy of this checklist will be published alongside your article.

Abridged guidelines for figures
1. Data
The data shown in figures should satisfy the following conditions:
- the data were obtained and processed according to the field's best practice and are presented to reflect the results of the experiments in an accurate and unbiased manner.
- ideally, figure panels should include only measurements that are directly comparable to each other and obtained with the same assay.
- plots include clearly labeled error bars for independent experiments and sample sizes. Unless justified, error bars should not be shown for technical
- if n<5, the individual data points from each experiment should be plotted. Any statistical test employed should be justified.
- Source Data should be included to report the data underlying figures according to the guidelines set out in the authorship guidelines on Data

2. Captions
Each figure caption should contain the following information, for each panel where they are relevant:
- a specification of the experimental system investigated (eg cell line, species name).
- the assay(s) and method(s) used to carry out the reported observations and measurements.
- an explicit mention of the biological and chemical entity(ies) that are being measured.
- an explicit mention of the biological and chemical entity(ies) that are altered/varied/perturbed in a controlled manner.
- the exact sample size (n) for each experimental group/condition, given as a number, not a range;
- a description of the sample collection allowing the reader to understand whether the samples represent technical or biological replicates (including how many animals, litters, cultures, etc.).
- a statement of how many times the experiment shown was independently replicated in the laboratory.
- definitions of statistical methods and measures:
  - common tests, such as t-test (please specify whether paired vs. unpaired), simple χ2 tests, Wilcoxon and Mann-Whitney tests, can be unambiguously identified by name only, but more complex techniques should be described in the methods section;
  - are tests one-sided or two-sided?
  - are there adjustments for multiple comparisons?
  - exact statistical test results, e.g., P values = x but not P values < x;
  - definition of 'center values' as median or average;
  - definition of error bars as s.d. or s.e.m.

Please complete ALL of the questions below.
Select "Not Applicable" only when the requested information is not relevant for your study.

Materials

Newly Created Materials	Information included in the manuscript?	In which section is the information available? (Reagents and Tools Table, Materials and Methods, Figures, Data Availability Section)
New materials and reagents need to be available; do any restrictions apply?	Not Applicable	

Antibodies	Information included in the manuscript?	In which section is the information available? (Reagents and Tools Table, Materials and Methods, Figures, Data Availability Section)
For **antibodies** provide the following information:<br>- Commercial antibodies: RRID (if possible) or supplier name, catalogue number and or/clone number<br>- Non-commercial: RRID or citation	Yes	Reagents and tools table

DNA and RNA sequences	Information included in the manuscript?	In which section is the information available? (Reagents and Tools Table, Materials and Methods, Figures, Data Availability Section)
**Short novel DNA or RNA including primers, probes**: provide the sequences.	Not Applicable	

Cell materials	Information included in the manuscript?	In which section is the information available? (Reagents and Tools Table, Materials and Methods, Figures, Data Availability Section)
**Cell lines:** Provide species information, strain. Provide accession number in repository **OR** supplier name, catalog number, clone number, and/**OR** RRID.	Yes	Reagents and tools table and methods
**Primary cultures:** Provide species, strain, sex of origin, genetic modification status.	Not Applicable	
Report if the cell lines were recently **authenticated** (e.g., by STR profiling) and tested for mycoplasma contamination.	Yes	Methods

Experimental animals	Information included in the manuscript?	In which section is the information available? (Reagents and Tools Table, Materials and Methods, Figures, Data Availability Section)
**Laboratory animals or Model organisms:** Provide species, strain, sex, age, genetic modification status. Provide accession number in repository **OR** supplier name, catalog number, clone number, **OR** RRID.	Not Applicable	
**Animal observed in or captured from the field:** Provide species, sex, and age where possible.	Not Applicable	
Please detail **housing and husbandry conditions**.	Not Applicable	

Plants and microbes	Information included in the manuscript?	In which section is the information available? (Reagents and Tools Table, Materials and Methods, Figures, Data Availability Section)
**Plants:** provide species and strain, ecotype and cultivar where relevant, unique accession number if available, and source (including location for collected wild specimens).	Not Applicable	
**Microbes:** provide species and strain, unique accession number if available, and source.	Not Applicable	

Human research participants	Information included in the manuscript?	In which section is the information available? (Reagents and Tools Table, Materials and Methods, Figures, Data Availability Section)
If collected and within the bounds of privacy constraints report on age, sex and gender or ethnicity for all study participants.	Yes	Methods and results

Core facilities	Information included in the manuscript?	In which section is the information available? (Reagents and Tools Table, Materials and Methods, Figures, Data Availability Section)
If your work benefited from core facilities, was their service mentioned in the acknowledgments section?	Yes	Acknowledgements at the end of the manuscript

Design

Study protocol	Information included in the manuscript?	In which section is the information available? (Reagents and Tools Table, Materials and Methods, Figures, Data Availability Section)
If study protocol has been **pre-registered, provide DOI in the manuscript**. For clinical trials, provide the trial registration number **OR** cite DOI.	Not Applicable	
Report the **clinical trial registration number** (at ClinicalTrials.gov or equivalent), where applicable.	Not Applicable	

Laboratory protocol	Information included in the manuscript?	In which section is the information available? (Reagents and Tools Table, Materials and Methods, Figures, Data Availability Section)
Provide DOI OR other citation details if **external detailed step-by-step protocols** are available.	Not Applicable	

Experimental study design and statistics	Information included in the manuscript?	In which section is the information available? (Reagents and Tools Table, Materials and Methods, Figures, Data Availability Section)
Include a statement about **sample size** estimate even if no statistical methods were used.	Yes	Methods, figures, and figure legends
Were any steps taken to minimize the effects of subjective bias when allocating animals/samples to treatment (e.g. **randomization procedure**)? If yes, have they been described?	Yes	Methods
Include a statement about **blinding** even if no blinding was done.	Yes	Methods
Describe **inclusion/exclusion criteria** if samples or animals were excluded from the analysis. Were the criteria pre-established?<br><br>If sample or data points were omitted from analysis, report if this was due to attrition or intentional exclusion and provide justification.	Yes	Methods and results
For every figure, are **statistical tests** justified as appropriate? Do the data meet the assumptions of the tests (e.g., normal distribution)? Describe any methods used to assess it. Is there an estimate of variation within each group of data? Is the variance similar between the groups that are being statistically compared?	Yes	Methods and results

Sample definition and in-laboratory replication	Information included in the manuscript?	In which section is the information available? (Reagents and Tools Table, Materials and Methods, Figures, Data Availability Section)
In the figure legends: state number of times the experiment was **replicated** in laboratory.	Yes	Figure legends
In the figure legends: define whether data describe **technical or biological replicates**.	Yes	Figure legends

Ethics

Ethics	Information included in the manuscript?	In which section is the information available? (Reagents and Tools Table, Materials and Methods, Figures, Data Availability Section)
Studies involving **human participants**: State details of **authority granting ethics approval** (IRB or equivalent committee(s), provide reference number for approval.	Yes	Methods and ethics approval and consent section at the end of the manuscript
Studies involving **human participants**: Include a statement confirming that **informed consent** was obtained from all subjects and that the experiments conformed to the principles set out in the WMA Declaration of Helsinki and the Department of Health and Human Services Belmont Report.	Yes	Methods and ethics approval and consent section at the end of the manuscript
Studies involving **human participants: For** publication of **patient photos**, include a statement confirming that consent to publish was obtained.	Not Applicable	
Studies involving experimental **animals**: State details of **authority granting ethics approval** (IRB or equivalent committee(s), provide reference number for approval. Include a statement of compliance with ethical regulations.	Not Applicable	
Studies involving **specimen and field samples:** State if relevant **permits** obtained, provide details of authority approving study; if none were required, explain why.	Not Applicable	

Dual Use Research of Concern (DURC)	Information included in the manuscript?	In which section is the information available? (Reagents and Tools Table, Materials and Methods, Figures, Data Availability Section)
Could your study fall under dual use research restrictions? Please check biosecurity documents and list of **select agents and toxins** (CDC): https://www.selectagents.gov/sat/list.htm	Not Applicable	
If you used a select agent, is the security level of the lab appropriate and reported in the manuscript?	Not Applicable	
If a study is subject to dual use research of concern regulations, is the name of the **authority granting approval and reference number** for the regulatory approval provided in the manuscript?	Not Applicable	

Reporting

The MDAR framework recommends adoption of discipline-specific guidelines, established and endorsed through community initiatives. Journals have their own policy about requiring specific guidelines and recommendations to complement MDAR.

Adherence to community standards	Information included in the manuscript?	In which section is the information available? (Reagents and Tools Table, Materials and Methods, Figures, Data Availability Section)
State if relevant guidelines or checklists (e.g., **ICMJE, MIBBI, ARRIVE, PRISMA**) have been followed or provided.	Not Applicable	
For **tumor marker prognostic studies,** we recommend that you follow the **REMARK** reporting guidelines (see link list at top right). See author guidelines, under 'Reporting Guidelines'. Please confirm you have followed these guidelines.	Not Applicable	
For **phase II and III randomized controlled trials**, please refer to the **CONSORT** flow diagram (see link list at top right) and submit the CONSORT checklist (see link list at top right) with your submission. See author guidelines, under 'Reporting Guidelines'. Please confirm you have submitted this list.	Not Applicable	

Data Availability

Data availability	Information included in the manuscript?	In which section is the information available? (Reagents and Tools Table, Materials and Methods, Figures, Data Availability Section)
Have **primary datasets** been deposited according to the journal's guidelines (see 'Data Deposition' section) and the respective accession numbers provided in the Data Availability Section?	Yes	GEO database. Accession numbers provided in reagents and tools table
Were **human clinical and genomic datasets** deposited in a public access-controlled repository in accordance to ethical obligations to the patients and to the applicable consent agreement?	Yes	GEO database. Accession numbers provided in reagents and tools table
Are **computational models** that are central and integral to a study available without restrictions in a machine-readable form? Were the relevant accession numbers or links provided?	Yes	Methods
If publicly available data were reused, provide the respective **data citations in the reference list**.	Yes	Methods and results
