# Supplementary material for: Intratumoral androstene-3,17-dione defines breast cancer subtype and prognosis
Source: EMBO Mol Med. 2026 Oct 1;18(11):4468–93. doi: 10.1038/s44321-026-00530-5 (PMC13748591; doi:10.1038/s44321-026-00530-5)
Supplement: Supplementary file 3 [file 44321_2026_530_MOESM1_ESM.docx]

|  | **ER-ve (N=26)** | **ER+ve (N=47)** | **Overall (N=73)** |
| --- | --- | --- | --- |
| **PR** |  |  |  |
| - | 24 (92.3%) | 15 (31.9%) | 39 (53.4%) |
| + | 2 (7.7%) | 32 (68.1%) | 34 (46.6%) |
| Missing | 0 (0%) | 0 (0%) | 0 (0%) |
| **HER2** |  |  |  |
| - | 22 (84.6%) | 39 (83.0%) | 61 (83.6%) |
| + | 4 (15.4%) | 7 (14.9%) | 11 (15.0%) |
| Missing | 0 (0%) | 1 (2.1%) | 1 (1.4%) |
| **AR** |  |  |  |
| - | 8 (30.8%) | 9 (19.1%) | 17 (23.3%) |
| + | 15 (57.7%) | 36 (76.6%) | 51 (69.9%) |
| Missing | 3 (11.5%) | 2 (4.3%) | 5 (6.8%) |
| **Ki67** |  |  |  |
| Mean (SD) | 52.0 (34.9) | 32.2 (25.2) | 39.7 (30.6) |
| Median [Min, Max] | 66 [0.90,94.0] | 24.0 [1.00,91.0] | 31.0 [0.90,94.0] |
| Missing | 1 (3.8%) | 6 (12.8%) | 7 (9.6%) |
| **Race** |  |  |  |
| AA | 17 (65.4%) | 16 (34.0%) | 33 (45.2%) |
| WA | 9 (34.6%) | 29 (61.7%) | 38 (52.1%) |
| Hisp. | 0 (0%) | 2 (4.3%) | 2 (2.7%) |
| **Age** |  |  |  |
| Mean (SD) | 58.0 (14.7) | 60.8 (12.9) | 59.8 (13.5) |
| Median [Min, Max] | 57.0 [31.0,88.0] | 59.5 [33.0,91.0] | 58.0 [31.0,91.0] |
| Missing | 0 (0%) | 1 (2.1%) | 1 (1.4%) |
| **Age (Binary)** |  |  |  |
| Low | 15 (57.7%) | 23 (48.9%) | 38 (52.1%) |
| High | 11 (42.3%) | 23 (48.9%) | 34 (46.5%) |
| Missing | 0 (0%) | 1 (2.1%) | 1 (1.4%) |
| **Stage** |  |  |  |
| 1 | 6 (23.1%) | 19 (40.4%) | 25 (34.3%) |
| 2 | 14 (53.8%) | 21 (44.7%) | 35 (47.9%) |
| 3 | 6 (23.1%) | 6 (12.8%) | 12 (16.4%) |
| Missing | 0 (0%) | 1 (2.1%) | 1 (1.4%) |
| **Grade** |  |  |  |
| 1 | 1 (3.8%) | 8 (17.0%) | 9 (12.3%) |
| 2 | 1 (3.8%) | 16 (34.0%) | 17 (23.3%) |
| 3 | 24 (92.3%) | 22 (46.8%) | 46 (63.0%) |
| Missing | 0 (0%) | 1 (2.1%) | 1 (1.4%) |
| **Recurrence** |  |  |  |
| None | 14 (53.8%) | 29 (61.7%) | 43 (58.9%) |
| Yes | 11 (42.3%) | 7 (14.9%) | 18 (24.7%) |
| Missing | 1 (3.8%) | 11 (23.4%) | 12 (16.4%) |

**Table EV1: Patient demographics and histological and clinical profile**: Seventy three (73) breast cancer specimens were collected from surgical suites for intratumoral hormone and transcriptome measurements. ER, PR, AR, and HER-2 expression were determined by immunohistochemistry. Proliferation marker ki-67 expression is represented as mean and median. Age was defined as low (age under 50 years) or high (age over 50 years). Tumor stage and grade classifications were defined according to TNM system. ER-estrogen receptor; PR-progesterone receptor; HER2-human epidermal growth factor receptor 2; AR-androgen receptor; AA-African American; Hisp-Hispanic; CA-Caucasian American; TNM – Tumor, Nodes, Metastasis system
