# Supplementary material for: Intratumoral androstene-3,17-dione defines breast cancer subtype and prognosis
Source: EMBO Mol Med. 2026 Oct 1;18(11):4468–93. doi: 10.1038/s44321-026-00530-5 (PMC13748591; doi:10.1038/s44321-026-00530-5)
Supplement: Supplementary file 4 [file 44321_2026_530_MOESM2_ESM.docx]

|  | **Model 1** | | **Model 2** | |
| --- | --- | --- | --- | --- |
| **Variable** | **HR (95% CI)** | ***P* value** | **HR (95% CI)** | ***P* value** |
| **A4 cluster** |  |  |  |  |
| A4-low (reference) |  |  |  |  |
| A4-inter | 1.175 (0.566-2.442) | 0.6649 | 0.125 (0.035-0.448) | 0.0014** |
| A4-high | 0.593 (0.208-1.686) | 0.3268 | 0.05 (0.009-0.29) | <0.001*** |
| **PAM50 subtype** |  |  |  |  |
| Luminal A (reference) |  |  |  |  |
| Luminal B | 1.557 (0.605-4.003) | 0.3582 | 0.09 (0.013-0.612) | 0.0138* |
| HER2-enriched | 4.166 (1.629-10.655) | 0.0029** | 1.204 (0.11-13.137) | 0.8791 |
| Basal-like | 3.848 (1.384-10.701) | 0.0098** | 0.28 (0.053-1.488) | 0.1351 |
| Normal-like | 1.031 (0.283-3.756) | 0.9626 | 2.636 (0.437-15.898) | 0.2904 |
| **Age** | 1.053 (1.031-1.075) | <0.001*** | 1.052 (1.029-1.074) | <0.001*** |
| **Tumor stage** |  |  |  |  |
| Stage I (reference) |  |  |  |  |
| Stage II | 1.184 (0.497-2.821) | 0.7027 | 1.386 (0.571-3.367) | 0.4705 |
| Stage III | 4.459 (1.947-10.21) | <0.001*** | 5.581 (2.317-13.446) | <0.001*** |
| **MKI67** | 0.874 (0.66-1.157) | 0.3457 | 0.958 (0.723-1.269) | 0.7645 |
| **Interaction** |  |  |  |  |
| A4-inter:Luminal B |  |  | 21.431 (3.027-151.754) | 0.0021** |
| A4-high:Luminal B |  |  | - | - |
| A4-inter:HER2-enriched |  |  | 3.11 (0.278-34.751) | 0.3569 |
| A4-high:HER2-enriched |  |  | 7.461 (0.378-147.162) | 0.1865 |
| A4-inter:Basal-like |  |  | 17.957 (3.422-94.23) | <0.001*** |
| A4-high:Basal-like |  |  | 12.864 (0.8-206.91) | 0.0715 |
| A4-inter:Normal-like |  |  | - | - |
| A4-high:Normal-like |  |  | - | - |

Multivariable Cox proportional hazards models.

Model 1 (main effects): A4 cluster + PAM50 subtype + age + tumor stage + MKI67

Model 2 (with interaction): A4 cluster × PAM50 subtype + age + tumor stage + MKI67

**Table EV2. Multivariable Cox proportional hazards models.** Hazard ratios (HRs) and 95% confidence intervals (CIs) were estimated using multivariable Cox proportional hazards models for overall survival. Model 1 included A4-associated cluster, PAM50 intrinsic subtype, age, tumor stage, and MKI67 expression as main effects. Model 2 additionally included interaction terms between A4-associated cluster and PAM50 intrinsic subtype. The reference categories were A4-low cluster, Luminal A subtype, and Stage I disease. Age and MKI67 were modeled as continuous variables.
