# Supplementary material for: Intratumoral androstene-3,17-dione defines breast cancer subtype and prognosis
Source: EMBO Mol Med. 2026 Oct 1;18(11):4468–93. doi: 10.1038/s44321-026-00530-5 (PMC13748591; doi:10.1038/s44321-026-00530-5)
Supplement: Supplementary file 5 [file 44321_2026_530_MOESM3_ESM.docx]

| **Model** | **Log-likelihood** | **df** | $\boldsymbol{\chi}^{\boldsymbol{2}}$ | **P value** |
| --- | --- | --- | --- | --- |
| Model 1 (main effects) | -325.79 | - | - | - |
| Model 2 (with interaction) | -317.29 | 7 | 16.99 | 0.017 * |

**Likelihood ratio test for interaction (Model 1 vs Model 2)**

**Table EV3**: The significance of the interaction between A4-associated cluster and PAM50 subtype was assessed by comparing nested Cox proportional hazards models using a likelihood ratio test (LRT). A significant interaction indicates that the potential prognostic effect of A4-associated clusters differs according to intrinsic breast cancer subtype.
