# Supplementary material for: Intratumoral androstene-3,17-dione defines breast cancer subtype and prognosis
Source: EMBO Mol Med. 2026 Oct 1;18(11):4468–93. doi: 10.1038/s44321-026-00530-5 (PMC13748591; doi:10.1038/s44321-026-00530-5)
Supplement: Supplementary file 6 [file 44321_2026_530_MOESM4_ESM.docx]

| **Variable** | **HR (95% CI)** | ***P* value** |
| --- | --- | --- |
| **A4 cluster** |  |  |
| A4-low |  |  |
| A4-inter | 0.247 (0.059-1.029) | 0.0547 |
| A4-high | 0.128 (0.021-0.769) | 0.0247* |
| **Age** | 1.149 (1.078-1.224) | <0.001*** |
| **Tumor stage** |  |  |
| Stage I |  |  |
| Stage II | 2.59 (0.683-9.813) | 0.1615 |
| Stage III | 3.974 (0.844-18.71) | 0.0809 |
| **MKI67** | 1.206 (0.659-2.206) | 0.5431 |

**Multivariable Cox proportional hazards model (Luminal A only)**

**Table EV4**: Hazard ratios (HRs) and 95% confidence intervals (CIs) were estimated using a multivariable Cox proportional hazards model restricted to Luminal A tumors. The model included A4-associated cluster, age, tumor stage, and MKI67 expression. Reference categories were A4-low cluster and Stage I disease. Age and MKI67 expression were modeled as continuous variables. Overall survival was used as the outcome measure.
